# Global access to technologies to support safe and effective inguinal hernia surgery: prospective, international cohort study

**DOI:** 10.1093/bjs/znae164

**Published:** 2024-07-10

**Authors:** National Institute for Health and Care Research (NIHR) Global Health Research Unit on Global Surgery, M Picciochi, A O Ademuyiwa, A Adisa, A E Agbeko, J A Calvache, D Chaudhry, R Crawford, A C Dawson, M Elhadi, A Ghaffar, D Ghosh, J Glasbey, P Haque, E Harrison, A Isik, I Jakaityte, S K Kamarajah, O Kouli, I Lawani, S Lawani, V Ledda, E Li, J Martin, A Minaya Bravo, D Morton, D Nepogodiev, F Ntirenganya, O Omar, S Z Y Ooi, R Oppong, F Pata, A Ramos-De la Medina, M Sampaio-Alves, J F F Simoes, M Steinruecke, S Tabiri, A Bhangu, A A Elhars, Y A Omar, S Ababneh, R Abad, A Abaidalla, E Abate, H Abbadi, A M Abbas, A Abbas, A Abbas, A Abbas, M Abbas, S Abbas, W Abbas, J Abbasy, B Abboud, M Abd Al-Fattah, R Abd Alkareem, R Abd Elkareem, A Abd Elsattar, N S Abd Ghani, D Abdalaziz, A Abdalhadi, A Abdalla, I Abdalla, S Abdeewi, H Abdelazim, E Abdelbaset, M Abdelfattah, M Abdelhadi Suliman Adam, M Abdelhafez, A R Abdelhalim, A Abdelhamid, M Abdelkabir, M Abdelkarim, M Abdelmaboud, M Abdelmaksoud, A Abdelmalik, S Abdelmohsen, A S M Abdelrahman, S Abdelrasoul Elnour Ismail, M Abdelreheem, M E E A Abdelsalam, A Abdelshafi, B Abdennour, A Abdllah, H Abdoun, A Abdraba, E Abdu, M Abdu, M y Abdualqader, A A Abdul Rahim, A Abdul Rahman, S Abdul Rahman, I Abdullah, M Abdullah, U Abdullah, L B Abdullahi, M Abdullahi, M Abdullahi, S Abdullateef, T M A Abdulmola, A Abdulnabi, N T Abdulraheem, D Abdulrahman, K Abdulrahman, T Abdulrahman, M Abdulsalam, L Abdur-Rahman, J T Abebrese, M Abed, S Abed, R Abela, T Abelman, S Abeykoon, D Abeysirigunawardana, R Abhinaya, A Abidali, A Abiodun, H Abiyere, M Abo Abdo, A Abo Al Shamat, Z A Abo Alarous, I Abo Elhagag, K Abo Zaal, C Aboah, A G E Aboelnasr, M Abosedra, S Aboseif, A A A Aboshosha, A G M M Abouelnagah, G Abouelnagah, O Abouhiekal, A Aboumedian, H Abozied, S Abrayik, A Abreu, A Abreu Da Silva, N Absy, M Abu Al Amrain, M K Abu Albahrain, M Abu Daoud, M Abu Jayyab, S Abu Khousa, H Abu Obead, R Abu Salah, S Abu Salem, E Abu Siam, A Abu Tair, H Abu-Arish, M Abubakar, A Abubakar Abdulkarim, M Abudabbous, A Abuhammad, A Abuhantash, A AbuNemer, D Abunemer, H Abuobaida, E E Abuobaida Banaga Hag El Tayeb, I Aburumman, H Abusnina, A Abuthaher, J Abutu John, M Abuwarda, S Abuzahra, M Acar, J D Acevedo Parrales, R Acharya, S Achugatla, L Ackam, E Ada, L Adagrah Aniakwo, A A Adam, E Adam, M Adamina, P Adebayo, E Adebunmi, A Adekoya, A Adel, I Adel, R Adel Diab, E Adel Hamdoun Aziz, E Adel Mahmod Sultan, A Ademuyiwa, A Aderounmu, M A Adetoyi, A Adewumiya, A Adeyeye, I Adham, H Adi, M Adinku, E Adinolfi, D Adjei, H Adjei, J Adjei, Y Adofo-Asamoah, G A Adoro, C Adumah, G Aduroja, W Afedo, E Afeikhena, N Affram, F Afonso, H Afzal, S Agana, M Agapov, C Agarwal, E Agastra, M Agbadebo, A Agbaje, V Agbakwuru, K Agbedinu, F M Agbemafoh, D Y D Agbley, N Agboadoh, F D A Agbodo, C Agbonrofo, P Agbonrofo, E Agbowada, B Ağca, O Agcaoglu, M Aggarwal, M Aggelidou, O Agha, A Aghayeva, S Agordjor, M K Agrawal, H Aguado López, S V Agudelo Mendoza, C Aguero, N Agugua-Obianyo, J A Aguilar, F Aguilar Del Castillo, M Aguilera-Arevalo, M Agunloye, M M Agyapong, R Agyei Boakye, T Agyen, J L Ahale, A Ahmad, A Ahmad, F Ahmad, H Ahmad, O Ahmad, A A H Ahmad Zaidi, A Ahmayda, A S Ahmed, A Ahmed, H Ahmed, E A Ahmed, G M G Ahmed, I M G Ahmed, I Ahmed, I Ahmed, M Ahmed, M Ahmed, M Ahmed, R Ahmed, S Ahmed, S Ahmed, K Ahmed Ibrahim, M Ahmoud, S Ahuja, F Aigner, M Aikins, R Ait Ben Addi, A Ajayi, S A Aji, O Ajiboye, E B Akakpo, R Akankoatuesi Apatewen, G Akcakoca, A Akeem Aderogba, C Akgün, T Akharaekpanya, F Akhtar, A Akiba, J Akiely, N Akinbami, A Akinkuolie, A Akinmade, F Akinwande, M Akkawe, C Akli-Nartey, A Akmercan, R Akpaka, U Akram, M K Aktas, K Akter, T Akter, Y E Aktimur, S Akuffo, F Al Abbood, O Al Alyani, N Al Amri, H Al Atassi, S Al Athath, A M Al Balushi, Z Al Balushi, T Al Barhi, J Al Daradkah, O Al Hamdani, A Al Hammoud, K Al Hinai, M Al Hinai, A Al Kaddah, J Al Karmi, H Al Miskry, S Al Momani, A Al Mukhtar, H Al Qadhi, N Al Rabadi, M Al Sayed, O Al Shaqran, S Al Sharie, R Al Shehhi, H Al Sohabi, B Al Soudi, M Al Zebda, H Al-Aamri, H Al-Abdallat, M Al-Attraqchi, Z Al-Azher El-Hamel, A Al-Bahla, Z Al-Balushi, L Al-Boukhari, H Al-Derume, M Al-Dhaheri, S Al-Doghme, H Al-Fahel, O Al-Fahel, A Al-Fandi, M Al-Fraijat, A B A Al-Hajjaj, N Al-Hroub, M A Al-Juaifari, Y Al-Junaidi, W Al-Khyatt, A Al-Mallah, M Al-Masri, A Al-Mouahhed, M Al-Qannas, B Al-Sarireh, K Al-Shami, M Al-Shehari, Z Al-Sheikh Ali, S Al-Tahayneh, M A Al-Yusuf, H Al-Zoubi, A Al-Zubeidy, O Alaba, E Alabed, A Aladl, A Alagha, F Alahmad, A Alahmad Alismael, A Alailesh, J Alaji, F Alakaloko, Y Alalawi, A Alamin, S Alananzeh, M Alansary, A Alaqtash, N Alasbahi, S Alashhab, F Alassani, R Alatawi, Y Alawneh, A Alayed, B Alazabi, M Alazabi, A Albager, N Albahloul, B Albaihani, A Albalawi, H Albalawi, J Albalushi, M E H Albanna, A Albatanony, A Alberico, M J I Albert, D Alberti, S Alberti, M Albertsmeier, A Albhaisi, T A Albushary, H Aldare, Y Aldebasi, A N Aldirani, T Aldirawi, A Aldurssi, A Alecci, G Alemayehu, P Alexander, D Alexandrou, M Alfaid, H Alfakeer, F Alfarsi, A Alfarwan, B Algettawi, G Alhadwah, A Alhaj, T Alhaj Hasan, A Alhaj Zain, A Alhamadi, A Alhammali, Y Alhammoud, M Alharthi, A Alhebshi, M Alhimyar, W Alhroub, H Alhusaini, T Alhusban, A Ali, A Ali, A Ali, A Ali, B Ali, B Ali, E Ali, F Ali, F Ali, I Ali, I Ali, M A Ali, M Ali, M Ali, N Ali, N Ali, M Ali-Hassanzadeh, E Alić, D Alifoe, s Alijla, R Alinde, T Alinger, S Aliozor, F Aliskander, M S Aliyu, G Alıcı, I Aljada, A Aljahdali, Z Aljalabi, A Aljamoudi, M Aljarawn, S Alkadi, A Alkaseek, Z Alkhaier, E Alkhalifa, J Alkharish, A Alkharusi, H Alkhatib, R Alkhatib, Z Alkhuzaie, A M Allam, S Allam, L Allan, Y Allaoua, B Allbakosh, J C Allen, C Allmer, R Alloni, A Alloush, W J N Almadhoun, R K Z Almahadin, T Almahdi, M Almahjoub, M Almaletti, M Almaqrahi, M M Almihashhish, G Almogy, O Almomani, A A Y Almugaddami, A Almutairi, Z Alnajjar, N Alnamari, F Alnazawi, M Alneasan, S Alneihuom, A Alniemi, I Alnimer, F Alnimri, M Alnuwayli, L Alokshi, V Alonso, S Alonso Marcos, T Aloqaili, M Aloulou, F Alowjaly, C Alphonse, M Alqadasi, B Alqahtani, R Alqahtani, M Alqedrh, A Alqerem, S Alqurashi, A Alrababah, A Alragheai, M Alrantisi, B Alrayes, G Alrayyes, A Alrifaee, L Alriyami, D S Alrokh, A Alroobi, O Alruwaili, W Alsado, S Alsaeiti, B Alsaid, A A Alsalahat, M Alsalawi, M Alshaar, S Alshab, S Alshagrawi, A Alshahrani, B Alshaikh, M Alshami, M Alshamikh, M Alshanwani, B Alsharari, M Alsharayri, A H ALSharqi, T Alshawabkeh, M Alshehri, A Alsheikh, G Alsheikh, A Alshiteewi, F Alshreef, A Alshukre, S Alsibai, S Alsmadi, M Alsori, I Alsoubie, A Alsultan, S Alsuwiyah, H Altabbaa, S Altaf, M Altajouri, A A Altawaiha, H Altawati, R Altayargh, M Althomali, Y Altinel, O Altobaishat, S Altoume, H Altounsi, A Alusef, R Alvarado Hurtado, A Alvarez Cuiñas, J M Alvarez Hernandez, D G Alves, N Alwaer, A Alwali, A Alwali, S Alwardi, N K Aly, A Alzadjali, A Alzahrani, N Alzerwi, B Alzoubi, A Alzu'Bi, R Alzu'Bi, R Alzughayyar, H Alzuhd, M Amadu, C Amah, S Amahmid, A Amaigl, D Amangaliyev, I Amankwaa, G Ambriz González, M O Ameen, A Amendola, E Amer, E Ametefe, A Amin, S Amin Omar Alsiddig, H Amin-Tai, B Aminu, F N Amir, H Amir, M F Amisano, A S Ammar, F Amoako, M Amoako-Boateng, L Amosu, H Amoudi, S Amoza Pais, J Ampadu, K Anaam, A Anadani, A Anand, A Anand, H Anand, M Anandan, P Anandan, A Anastasi, M Anati, S Anaya Sanchez, D Anbu, A D Andani, E Andolfi, N Andrade, S Andrade, P Andrejevic, R Andreou, L Andreski, J Andreuccetti, E Anestiadou, A Ang, M Angelini, M Angelucci, S Anim, R Anjum T Siddeek, J Annan, P Anoldo, C Ansah Larbi, G Ansong, A Antinori, P Antonakis, E Antoniou, A A A Anuar, L Anyanwu, J N Anyorigiya, F Anzinger, H Aouagbe Behanzin, H Aouagbe Behanzin, D Aparicio-López, E Apostolopoulos, A Y Appiah-Kubi, A Appukuttan, G Aprea, S Arafa, M Arafat, M Araydah, H Arbogast, A B Aregawi, A Arekhandia, I I Aremu, R Arena, N Arkadopoulos, E A Arkoh, R Armah, V E Armenta Tapia, A Y Arnaout, I Arnaout, M Arnaout, A P Arnaud, O Arsalan, P C Arteaga Asensio, S J Arthur, P Artigot, N Aruldas, P Arunachalam, A Arustamyan, B Arya, P K Arya, H Asaad, M Asaad, Z Asaad, Y Asano, Y Asar, M Asarbakhsh, A Asare Twumasi, F Ascari, K V Ascencio Diaz, A Asekun, J Aseme, L Asensio Gomez, A A Asharaf, S Ashini, A Ashiru, D Ashitey, T Ashkar, M E Ashong, Y Ashour, F Ashraf, F Ashraf, S Ashry, M Asimakidou, S Asiri, R Askari, H Askarpour, A E Askin, A Asla, A Aslan, M Assalhi, C Assis, P Assouto, J R Asturias Luna, M I Ateş, A Athanasiou, E Athanasopoulou, K Athanassiou, K Atherton, D Atouguia, R Attaf, O Attawodi, D Attia, A Attili, S A A Atupra, G Augustin, M Aulicino, Y L Aung, A Autiak Ayii Chol, J Avakoudjo, M A Avci, K Avgerinos, A Awad, A K Awad, S Awad, T Awad, G Awais, A Awidan, A Awwad, N A Awwad, E Ay, K Ayad, E Ayala, O Ayandipo, F Ayasra, E Aybar, A Ayeni, H Ayesh, A Ayoade, B A Ayoade, A Ayodele, K Ayyoub, A Azam, B Azhar, Z Azhar, R Azimov, M Aziz, C E Azmat, A Azzinnaro, M M R Azzuz, S A M Ba-Shammakh, C A Baars, I Babajimi-Joseph, O Babatunde, S Babu, B Baca, E A Baccalini, I Bacic, F G Bader, M Badkur, Y Badr, R Badra, D Badran, B Badwan, Z Bady, I W Bahikoro, M Bahnacy, O Bahsas-Zaky, A Baiden Amissah, E Baili, L Bains, M Bajwa, A Bakalis, I N Bakaweri, E Baker, A Bakri, V Bakshi, A Baksi, B Balagobi, D Balalis, B Balaravi Pillai, Z Balciscueta, A Balconi, R Baldan, E Baldini, E Balik, O Balogun, Y L Balogun, A Balta, C Banal, K Bananis, F Banchini, S Bandyopadhyay, N Banerjee, A Bang, A Bangar, S Bani Amer, M Bani Hani, C Banka, E Bankart, D Baños Méndez, E Bara, A Barakat, M Barakat, M M Barakat, M Barbarawi, A Barberis, F J Barbosa Camacho, S Barbosa Castelo Branco, M D Barcelona, A Barcin, D Bardsley, J Barklimore, E Barkolias, A Barlas, L Barnard, L Barni, R Barone, G Baronio, R Barradas, A G Barranquero, N G Barrera Lopez, M T Barrio Renteria, J Barrutia Leonardo, A Bartalini Cinughi De Pazzi, A Basalim, H Basharat, M Bashir, M A Bashir, G Bashour, E Basile, R Basile, R Bassam, A Bassma, S M M Basso, S Basu, S Basu, S Bataineh, R Batir, S Batista, M Battili, M Bautista, A Bavaharan, A Baydoun, N S Bayleyegn, H Baysal, S Beavis, G Bechara, O Bedak, A Bediako Bowan, A Bedzhanyan, E Bedzhanyan, E Begunić, N Beharry, M Bei, M Bejarano Serrano, G Bekakos, K Bekiaridou, S Bektas, A Belaid, A Belarmino, D Belekar, M Beletachew, A Belkhair, A Belli, M I Bellini, G Bellio, F Bello, M Bello, O Bellou, P Belotti, R Belouz, J Beltrán De Heredia, N Ben Hasan, R Ben Hmida, M Benamrouche, Z R Benamrouche, B Bengaly, F Benghalbon, M Benghazi, A Benmansour, S Bennett, S Bensalem, K Bensmain, M A Bequis, B Bereket Araya, G Berger, M Berlet, R Bernardino, M Bernardo, P Bernardo, M Berrakkouch, F Berrevoet, M Berselli, C L Bertoglio, S Bertone, M Bertrand, R Bethune, E Betz, L Beukes, M Bevanda, C Bezede, A Bhadani, A Bhargava, G A Bhat, M Bhat, N Bhat, R Bhatia, A Bhatt, A Bhatti, H W Bhatti, K Bhatti, W Bhatti, K Bhutia, A Biancafarina, M Biancu, A Bibi, N S Bibila, E Bičakčić, M Bickford, M Biebl, H Bileid Bakeer, M Bilfaqirah, I A Bilgin, C Bilir, V Bill, M Billis, L N Bin Aizan, M M Bin Khalid, B Binda, M Binda, A Binder, J Binder, M Binks, N A Binti Yusri, F Biolchini, G Birikorang, S L Birolo, G Birqeeq, E Bisogno, S Bitsianis, M Blanco, J L Blas Laina, N Blencowe, N S Blencowe, B Boakye, K Boakye-Acheampong, P A Boateng, P K Boateng, C Bode, M Bogdan, B Bogdan-Gabriel, M Bohlala, A Bojazyah, I Bokos, C Bokossa, L I Bolaños, J Bolanowski, H Bolukbasi, G Bond-Smith, A Bonelli, L Bonello, L D Bonomo, K Booyse, M Boras, G Borda-Luque, A Borello, E Borges, I Borges Da Costa, V Borgogni, N Börner, G Boroni, S Borrego Canovaca, S Botaitis, A Botha, C Bôto, K Bouchagier, I E Boudis, M N Bouhafs, A Bouhuwaish, O Boujidi, K Bouliaris, I E Boumakhlouf, N Boumas, C Boven, M Bowles, S Bowman, S Boyes, A Boynes, K Bozada-Gutiérrez, E Bozdağ, A Ç Bozkurt, E Bozkurt, A E Boztaş Demir, L Bracciano, G Brachini, R Bradley, H Bradly, C J Bradshaw, S Bradulskis, H Braham, K Bramis, T Branco, C Brandi, R Branquinho, A Brar, C Brasset, L Bravo, L D Bray, Z Brekalo, S G Brenu, S Brincat, M Brito, M Brizzolari, V Brocco, A Broglia, E Brolese, L Bromley, A Brosin, G D Brown, F Brucchi, A Brun-Peressut, M Brunner, J Bryan, F Brzeszczyński, S S Budhwani, V Budyakova, N Bugdayci, S Bugren, F M Bujalance Cabrera, G C Bulbuloglu, J Bundred, S Burger, M Busch, M F Butt, A Butyrskii, F Buyuker, K J Bwala, G Cabezudo, L R Cabezudo, A Cabral, N N Cabrel, I Cabrera, P A Cabrera Rivera, M Caccetta, R Cachia, E P Cagigal Ortega, S Cai, F Caiquo, I Caitens, I Çakır, A Çakmak, B Cakpo, B Calcaño, M Calderon, B Calik, V Çalik, G Calini, P G Calò, A Caltagirone, F Camacho, O Camilleri, F Cammelli, M Cammelli, M Campanelli, A Campbell, R Campbell, F Campus, M Camuera, M Canals Sin, O Čančar, M D Cancelas, G Canonico, G L Canu, B Capdevila Vilaró, L Capello, H Capote, F Cappellacci, M Cappiello, M Capuano, L J Caram, M M Caramantin Obando, L Carbone, M Carbonell Pradas, S Cardelli, G Cárdenas Rivera, L Cardinali, D C Cardona Gomez, C Cardoso, A Cardoso Almeida, G Carganico, J M Carlos, P Carmignani, G Carollo, O Carpineto Samorani, Y Carpio Colmenares, S Carrabetta, J M Carranza Rosales, A L Carreira-Marques, C Carrillo Sarango, J Carrizo, A Carta, D Caruso, B Carvalho, M Carvalho, L Casalduero, F Casas J, M Casati, J R Casella Mariolo, L Casingena, R Casma Bustamante, J Cassiano Neves, A Castaldi, F Casti, F Castro, L Castro, R J Castro Lara, M Castrovillari, L Catozzi, M Catterall, M D P Cebollero, C Cecconi, C Celano, N Celestine, B Celik, A Cembellin, D C Centonze, M Ceolin, M Ceresoli, A Cerovac, I Cervera, A F Cetişli, M Chaccour, P Chadha, F Chahrour, S Chaifouroosh Mamagany, D Chakarov, Y Chamara, G Chamoso Mialdea, A Chamzin, E Chan, J Chan, M Chandrasegaram, G Chang, L Chang, L Chantada, G Charalambous, L Chardalias, S Charitonos, S Chase, D Chatterjee, P Chatzikomnitsa, C Chatzinikolaou, I Chatzis, A Chaudhary, R Chaudhary, S Chaudhary, A Chaudhry, N K Chaudhry, H Chauhan, M Chauhan, C Chavarría Noya, N Chavarrias, D Chavez Fernandez, M Che Yaacob, H K Cheema, J M Chejfec-Ciociano, A J Chekfa, J J Q Chen, N Chen, L P Cheng, C Cheong, A C K Cheung, M Chevallay, J Chew, A Chhabra, S Y D Chia, P Chiacchio, C Chiam, A Chiappini, N Chidumije, A Chied, L R Chieng, S P Chigblo, S Chilaka, G Chillarge, B G Chiman, P Chimezie Andrew, D Chimkaomasiri, E Chin, Z Chin, M H Chishti, M Chisthi, A Chitul, B Chkir, P Chloropoulou, I Cholah, K Cholah, J Chóliz, L Chong, S Chopra, T Chouari, A M Choudhary, A Choudhrie, A Choudhry, C Chouliaras, S A Chowdhury, S Chowdhury, N A Christian, N Christian, O Christianah, G Christodoulidis, P Christodoulou, S Christodoulou, M Christou, K Chu, E Cianci, P Cianci, D Cianflocca, F Ciccarone, E Ciccioli, P M Cicerchia, L Cidade Costa, A B Ciftci, V Cijan, N Cillara, C Cini, E Ciofic, B Cirillo, B Cismasiu, B Citgez, C Ciubotaru, D Clarke, I Clementi, L Cobellis, N Cobeño Tamayo, G Cocorullo, L Codina Corrons, O Cohen-Arazi, I Colaço, E Colak, R Colbran, K Collins, E Colton, A Comandatore, A Comella, S Condron, L Conti, I Contis, I Conversano, F Coratti, M Corcos, B Cordeiro, D Córdova García, C Cornwell, M Coronas Soucheiron, Z M Correa López, P Correia, F Corronca, S Corso, F Corvatta, C Costa, S Costa, A Costas-Chavarri, C Coutinho, G Cox, O Coyoy-Gaitán, I Ćoza, M Cricrì, D Cristian, A Crowe, M Cruz, O M Cuenca Torres, M Cuesta Argos, M Çuhadar, J Cui, V Cuk, B Cuneo, A Curado Soriano, D A Sathiafrey, F d'Acapito, F D'Agostino, A D'Amore, M R D'Anna, A D'Ignazio, B D'Souza, C Da Lio, H Daaboul, D Daary, E Dabbagh, H Dąbrowski, N Dadoush, N Dafnios, M Daher, A Dahiru, E Dainius, G Dajti, I Dajti, E Daketsey, H Dalati, J Daleku, E Daleva, C Dally, C Damaskos, A Damigella, A Damola-Okesiji, R Damseh, M Daniyan, R A Dar, R Daradkeh, A Darkwa Boateng, A Das, K Das, M Dason, U Daspal, V Dassah, I Datji, A Dauksa, Z Dauksa, E Daukšaitė, M Dauphin, S Davakis, P David Santos, B Davies, A Davis, S Davis, A Davolio, A Davor, S Dawo, A Dawoud, B Dawud, B De Andrés-Asenjo, B De Andrés-Asenjo, G De Angeli, G De Carlo, J De Deken, V U De Donato, J M De Francisco Rios, I De Haro Jorge, Á De Jesús Gil, S De La Cruz Ahufinger, G De La Peña González, E De Leo, C De Martino, P De Nardi, G D De Palma, M De Prizio, N De Santis, W D D De Silva, Y De Silva, G De Wee, B De Zolt Ponte, Y Dean, S Debrah, J Deeb, L Degrate, J Dei-Asamoa, G Del Corpo, P Del Val Ruiz, H Delacave, G Delgado Hernandez, S Delibegovic, D Dellaportas, A Dembele, K S Dembele, A Demessie, Z Demetrashvili, A Demetriou, F Demiral, C Demiri, I Demiris, M T Demirpolat, J A Demma, C Dempers, B Demurtas, M Denini, A Denz, M Derattani, M Dery, A Deserra, S Deshpande, E Desiato, M Despotidis, T Devabalan Koil, A Develioğlu, H Devesa, L Devi, G Devidze, B Devkaran, P Devlin, E Dexter, K E Dey, P Dhamija, D Dhawan, J Dhillon, J Dhima, J Dhiman, A S P Dhinakar, S Dhole, G Di Franco, E Di Marco, F Di Marco, D Di Pietrantonio, S Di Saverio, M Diab, J Dias-Ferreira, F Diaz, R Diaz, R Díaz Pedrero, R Díaz-Ruiz, O A A Dicko, I Didier, B Dieck, U Dietz, M Diez Alonso, E Dijan, U Dilibe, N Dimitriou, N Dimitrokallis, D Dimitrov, C Dina, S Dindyal, V P Dinuzzi, N Diomede, C Distefano, Z Djama, S M Djote, J E Do, R Doamba, M Á Dobón Rascón, A Doghaim, S Dognia, R Doherty, C Doku, M A Dokurugu, Q K Dolatzay, H Dolo, T Doma Bhutia, A Domenech Plana, J C Domingues, J Dominguez, R Donchev, I Dong, J Dongmo, M A Doniquian, A E Dönmez, E Donnarumma, C Donohoe, E A Dontoh, P Dorovinis, S Dos Santos, S Dos Santos Rocha Ferreira, M Doss, F Dossou, C Doudakmanis, B Down, E Downes, E Downing, V Dragisic, R Drasovean, M Drogouti, L Dubs, J A Duffield, D Dugar, M Dugo, M Dum, B Duman, P Dummala, A Dupont, V Duque Mallén, M E Duran, I I Durán Sánchez, G Duranti, Z Durna, A Duro, M Durrani, N Duru, A Dusabimana, R Duvenage, E Dvivedi, B Dweik, M F Dwikat, L Dyab, M Dyer, E Dylja, S Eam, N Eardley, B East, D Eaton, H Ebied, S Ebrahim, W Ebrahim, J Ede, M Edeeb, S Edem, M Edena, M Ediale, R Ediru, U Edith, A Edmundson, E Efremidou, A Efstathiou, E Efstathiou, E Efthymiou, A Egdeer, R A Eghonghon, S Egreara, O A Egwuonwu, A Ehab, A Eisa, L Eisner, M Eissa, H Ejaz, E Ekaladze, M T Ekanayake, G Eke, A Ekerin, A Ekpeti, N Ekwo, H Ekwuazi, O H Ekwunife, M M El Ghoul Miliotou, G N El Hunjul, A E S El Kady, Y El Okazy, A El Shamarka, A El-Bastwesy, A El-Sherbiney, R Elaifia, R Elazary, A Elbalal, S Eldirdiri, O Elebute, P Elemile, M Elesseily, S Elfallah, H Elfeki, M ElFiky, S Elfurdag, M A Elgak, L Elgebaly, H Elghadban, M A M Elghriani, A Elghrieb, F Elhabishi, H Elhallaq, O A Eljizoly, D Elkafrawy, F Elkhafeefi, A Elkhalifa, A S Elkhodary, A M Elkhouly, R Ellawala, C Elliot-Wilson, S Ellouzy, M Elmesalmi, M Elmiesiry, A Elmosalamy, M Elmousili, M Elmujtaba, S Elnoamany, M Elnour, R Eloka, A M Elsayed, M Elsayed Metwally, A Elshazli Mahmoud, E Elsheikh, E Elshennawy, R Elshennawy, A Eltahir, A Eltayeb, A Elzanaty, M Elzayat, A E Elzoubi, O Emadeldeen, H Embarek, S Emile, G Emiliani, A Emran, O Emuze, J Enaholo, O Enciu, R E Enejo, V Enemuo, S Engel, D Enjuto, N Ensor, M Enßlin, L Epis, G Ercolani, M Erfan, M Ergenç, E Erginöz, M Erkaya, S Errami, C Ersavas, J Escartin, N Eshak, F J Eshun, I Esparza Estrada, B Esposito, H Essalim, A Essamei, M Estaire Gómez, C Esteo Verdu, B Estraviz, V Etwire, M Eugene, P Evangelou, A Evans, D Evripidou, M Ewedah, K Ewool, B Eyduran, J Ezeh, U Ezidiegwu, U Ezomike, N Fabbri, S Faber, O Faboya, M V Facchino, A Fadipe, H A Fadlalmola, F Fadzlullah, M Fagbayimu, I Fagiri, J Fahed, B Fahmy, M Fahmy, D Fairbrass, A Faisal, M Faisal, R Faisal, A Fajardo, S Fakhouri, I Fakhradiyev, A Fakoya, M Faletar, A Fani, Y Farag, H Farhat, M A Farho, M Farooq, M R Farooqui, A Farrag, Z Farraj, G Farris, M Farrugia, A Farsi, O K Fasiku, O Fasoro, A Fathi, A Fatima, L M Fatucchi, O Fatudimu, A Faustino, M Favoriti, P Favoriti, K Fayed, J M Faylona, N Fazili, J Febré, L Fedele, H Fehrer, N Feijoo, S Fennelly, D Fenner, C V Feo, R L Ferlini, J Fernandes, U Fernandes, Á Fernández Camuñas, M A Fernandez Romero, M A Fernández Zurita, F Ferraina, F Ferrara, A Ferreira, M Ferreira, R Ferreira Acosta, C Ferreras García, C I Ferrero, E Ferri, G Fialho, I Fidoshev, C Figueiredo, J Figueiredo, R Figueroa, M Filardo, T Finlay, N Finocchiaro, A Finocchio, M Fiogbe, Y Fishman, M Fitri, M Flint, L A Flores Chávez, L M Flores Chávez, J A Flores Prado, C M Florián Villa, F Floris, C Floros, M Florou, H Foad, H Foda, G B Fonsi, J Fontaínhas, L Fortuna, S Fortuna Martins, I Fortune, B Fosh, L I Fountarlis, L Fountoulis, A Fox, Z A Fozo, D Fra Corral, E Fradelos, G Fragulidis, A Fraile, A Francia, E Francis, S Francis, A Franco, N F Franco, P Francúz, A Frankel, R Franz, E Fraser, R Fratarcangeli, J Frazão, B Freire, N M Freitas Oliveira, K Fretwell, E Frimpong-Manso, F Frongia, D Fry, J H Fu, C Fuentes Orozco, L Fuentes Rivera Lau, A N Fuertes Muñoz, R Függer, Y Fulgence, C Fumagalli, N Furbetta, D Fusario, L Gabellini, O Gabom, T Gach, K Gaikwad, M Galasyuk, S Galati, G Galatioto, J Galea, F Galley, E Gallo, G Gallo, A Galvan, Y Galvañ Félix, B Gama, M D C Gama Caldeira, S L Gamba, M Gambelli, S Gananadha, L Gantner, D S Garcés Palacios, M Garcia, J S García, A García Domínguez, J L Garcia Galocha, S García López, D Garcia López De Goicoechea, A García Marín, E P Garcia Santos, M Á García Ureña, J C Garcia Vera, P Garcia-Dubus, J García-Quijada, C García-Salas, G García-Santos, E Y García-Villegas, L García-Zambrano, L Garciandia, F Gareb, S Gargiulo, M Garino, N Garmpis, I U Garzali, C Gas, M Á Gascón Domínguez, I Gascon Ferrer, É Gáspár, S Gaspar Reis, J Gass, D Gavit, E Gavriil, A Gbeadese, G Gbessi, A Gegúndez Simón, K Geiger-Timm, M S Genç, V Gentilino, D George, G George, M George, P Y George, F Georgiades, D Georgiev, V Georgilaki, R Geow, S Gerdes, D Gero, I Gerogiannis, A Gerundo, E Gessa, G Getachew, R Ghaleb, Y S S M Ghaleb, A Ghanbari, A Gharib, S Ghattas, A Ghazal, A Ghazal, F Ghazali, A Ghummied, M Ghunaim, A Giakoustidis, D Giakoustidis, E Gialamas, K S Giannakopoulos, S Gilbert, A Gill, S Gill, J Gillingham, T Gimenez Maurel, E Gimson, C Ginesta, R Gioco, V Giordano, S Giovampietro, G Giraudo, S Giridaran, T Girma, J E Giubi Bobeda, S Giudici, M Giuffrida, A Giuliani, M Giuliano, D Giulitti, M Gjoka, S Gkogkos, A Gkouniaroudi, A Gkoutoula, P Glushkov, D Gobatti, S Gobishangar, J Goedeke, M Goga, I Gogoulis, N Gokhare Viswanath, A Goliath, S Göller, C Golliez, I Gomatos, F Gomes, M Gomes, J Gomez, S Gomez, D Gómez, E Gómez Mejía, T Gómez Sanz, H Gomez-Fernandez, D M Gonçalves Múrias Gomes, J G Goncalves-Nobre, V E Gonzabay, D S Gonzalez, M D C Gonzalez, C González, E González, J E Gonzalez Aboytes, C González Baez, M González De Miguel, J Gonzalez Garcia, E González Marín, A Gonzalez Ojeda, F Y González Ponce, M E Gonzalez-Gonzalez, G R Goodwin, F Gool, S Gopaul, A Gori, M Gosselink, A G Goswami, I Gouazar, R Goudou, N Gouvas, S Govender, U Govindan, R Govindaraju, V Govindasamy, C Gracia-Roche, F Grama, M Gramellini, R Granata, V Granata, G Grande, M Grande, L Granero, G Grassi, G Grava, G Gravante, A Gray, G Graziano, L Green, K Griffin, S Grimaldi, M Gritti, P Grivas, P Grondona, J Grosek, U Grossi, R Grützmann, S Guadagni, S Guarriello, G Guercio, R Guercio, B Guerra, M Guerrero, J Guevara, C Guijarro Moreno, I Guirguis, R R Gujjuri, O B Gülcicek, C E Guldogan, A Gulla, T Gülşen, S Gumede, M Gumsheimer, M Gün, V Gunasaegaram, A Gupta, A Gupta, A Gupta, H Gupta, M Gupta, N Gupta, P Gupta, R Gupta, M Gureh, M Gurung, J Gusa, J B Gusa, A Gusibat, A E Gut, E Gutierrez, C E Gutierrez De La Rosa, J A Gutiérrez Gómez, J A Guzman Barba, R Guzmán Lambert, F E Gyamfi, A Gyedu, M H. Oweidat, R Habib, H Habiba, B Habrat, A D Hacioglu, C Hacıalioğlu, D Hackner, A Hada, F Haddad, F Haddad, R Haddad, D Hadzhiev, E Hadzhieva, E Haege, I Hagbevor, M Haghighat Ghahfarokhi, A Hai, A Haidar, E Haiden, M A Haider, Y Haido, M Haimona, H Haj Freej, M Haj Hussein, M Hajalamin, A Hajali, A Hajela, M Hajhamad, I Hajmohammed, S Halabi, E Halilović, K Hall, R Hall, M Hamada Takrouney, G Hamdan, H Hamdar, I H S Hamdun, E Hamed, M Hamed, A Hameed, B H Hameed, S Hammad, A R Hammadieh, A Hammed, F Hammett, M Hammoda, M Hamoud Almahly, I Hamzaoglu, R Handa, S Handa, S Hanna, D Hannedige, Z A Hannouneh, O Hany, B Z Hao, P W Haque, S Hariharan, C Harmston, M Harris, T Harris, N Harrison, G Hart, A Hasan, L Hasan, L Hasan, M Hasan, Y Hasan, A Hashmi, S Hassan, A Hassanin, F R H Hassooni, E Hatangimana, A Haty, R Hauwa Sani, L Havranek, A Hawarah, K Hayat, S Hayat, L Heeren, E Hegab, R Hegy, B Helou, C Henriques, S Henriques, D Herappe, M O Herdan, A Herewini, A Hernández, J R Hernández, B Y Hernandez Cervantes, C Hernandez Diaz, R A Hernandez Rodriguez, D Herrera, R Herrera, J O M Herrera Batres, I Herrero, T K Hessou, T K Hessou, A Hewari, R Hiary, C Hidalgo Salinas, N Hielscher, L Hijazein, M Hijazi, A Hilder, D Hili, A Hiller, S S Hlaing, R Hleyhel, G Hneino, R Hodgson, A M Hodonou, E Hodžić, E Hodžić, K Hofmann, P Holt, J Hong, C Hoogerboord, E Hopping, M Horga, A Horiguchi, P Horvath, C Hossu, D Houmran, F Hounde, S F A Houndji, F Hourri, I Hoxhaj, E Hristova, A Hrora, Y Huang, C F Huaroto Landeo, F Huda, P Hudáč, T J Hugh, S Hügli, A Huremovic, M Husain, S Husanov, N Hüser, R Hussam Yacoub Hattar, I Hussein, A Huws, J Hwang, A Iacomino, T Iahmo, M Ibadin, F Ibañez Ortiz, O Ibarra, S A Ibarra Camargo, T R Ibarra-Hurtado, G P Ibero Casadiego, B Ibi, T T Ibiyeye, B Ibrahim, D Ibrahim, H Ibrahim, M Ibrahim, O Ibrahim, S Ibrahim, A Ibrahim Hassan, S Ibrahim Tour Harakan, S Ideh, H Idheiraj, P Idjerhe, D Idowu, G Ietto, N Ildephonse, K Iles, K Iliakopoulos, I Ilieva, C Ilo, S Imam, L Imchen, I Imihteev, M Imirski, C Infante, M Infantes Ormad, S Ingley, S G Intini, A Ioannidis, O Ioannidis, V Ioannou, A Iodice, A Iqbal, M Iqreewi, F F Irakiza, H Irfan Khan, O Irowa, S Irrinki, A Irshad, E Irungu, M Ishak, M Ishak, A Ishola, H Islam, S Islam, A Isler, M A Ismael Alamin, N Ismaiel, M I S Ismail, S Ismail, M Issa, M I Issa, R Issa, A Issaka, H Iswariah, T Ivanov, B O Izedomi, A Izgiş, A Izzo, F Izzo, J Arya, J B Kavitha, R Jaba'Teh, M Jabal, D Jabbar, S Jabeen, A Jaber, H Jaber, W Jabłoński, H Jafarkhan, K Jaffry, J Jahjah, A Jain, D Jain, G Jalal, H Jalilehvand, C Jamieson-Grigg, S Jamil, M Janda, A Janjua, A Jarimba, S Jarmusch, K Jasaitis, K Jasso García, R Jayakrishnan, A Jayapalan, U Jayarajah, S Jayasekara, S Jayatilleke, M Jean Claude, N Jeannette, Y Jedidi, T Jemal, M R Jesudason, C Jezieniecki, A K Jha, V S Jha, N Jiagge, L Jiménez, J A Jimenez Flores, A Jimoh, A Jindal, A John, P John, A Johnson, B Johnson, Z Johnson, L Johnstone, M Jokubauskas, O Jolayemi, S Jomaa, J Jones, J J Jordaan, T W Jorgensen, E Jose, A Joseph, S Joseph, D Joshi, D Joshiba, R Joumaa, L Jovcheski, N Jović, A Jowharji, S Jp, L C Juan Carlos, R Jubran, J Juloski, M Jurado Román, R Jurdon, O Jurić, K B Manojkumar, K S D Manoj Kishan Lal, A K. Ali, A Z Kaan, K Kabulov, M J Kacem, S Kache, A Kachi, A Kadamani Abiyomaa, B Kadir, G Kafa, E Kafui Ayodeji, J Kahn, J Kaippally, M Kajic, V Kakotkin, M Kalabić, N Kalampokis, M C Kalaydjian, M Kalender, S Kalenga, A A Kalidis, M Kalisvaart, V Kalles, A Kalogeropoulou, R Kalouk, U A Kalu, J A Kalyanapu, A Kamal, H Kamal, M Kamal, M Kamal Matter, V Kamalathevan, M Kamar, S Kamari, K Kambouri, P Kamenova, E Kamer, C Kamphues, S Kanaan, P Kanavidis, I Kandil, L Kang, M Kang, V Kanna D, N Kansakar, U Kanyan Kassim, E Kapasakis, S Kapiris, M Kaple, G Kapogiannatos, P Kapsampelis, N Kapur, M E Kara, M A Kara, Y Kara, E C Karabulut, G Karadeniz Cakmak, C Karagianni, S N Karahan, T Karahasanoğlu, G Karakaidos, E Karakeke, M Karakeke, A KARAKOSTA, M Karamanliev, A Karamarkovic, Z R Karampotaki, S Karandikar, M Karanikas, Y Karataş, I Karatsolis, M Kariem, S Karim, N Karimbocus, S Karmarkar, N Karthikeyan, N Karunaratne, S Karuppusamy Krishnasamy, L Karydakis, D Kasasbeh, C Kaselas, P Kasetsermwiriya, W Kasetsermwiriya, M Kashif, S Kashyap, S Kassad, M Katif, H Kato, J Katogiritis, S Katorkin, S Katragadda, I Katsaros, L Katsiaras, L Katsiaras, A Katsiou, R Kattini, Z Katusic, G Kaur, H Kaur, H Kaur, H Kaur, M Kaur, P Kaur, R Kaur, R Kaur, S Kaur, M Kausar, D Kaushal, N Kavak, A Kavalakat, G Kavalieratos, A A Kayali, G Kazobinka, T Kebede, A Kedwany, D Kelgiorgi, M Kelly, A Kelzia, G Kenchadze, S Kenworthy, M D Keramida, I M Kereet, Y Kerolous, S Kessab, A Kezze, A Khair Etareig, A Khairy, A Khaity, M Khaled, K Khalid, M Khalid, M S Khalid, R Khalid, L Khalifa, M Khalifa, A Khalil, K Khalil, M Khalil, M Khalil, O Khalil, R Khalil, A Khalleefah, A Khamees, A Khan, B Khan, M F Khan, S Khan, S Khan, Z Khan, Z Khan, A Khanduri, S Khare, B Kharga, A Khatib, B Khattab, D H Khattab, S Khattak, A A Kheirkhah Vakilabad, T Khewater, N Kheyrbek, A Khoneisser, A Khouli, J Khoury, Z Khraim, S Khurana, K Khutsishvili, R Kibuuka, M Kießler, B Kigwe, C Killoran, A Kirschniak, M Kisielewski, R Kitamura, A Kiyabayev, M C Kizilkaya, N Kiziltoprak, N Kiziltoprak, N Klammer, J Kleeff, M Klib, Z Klib, C Kloppers, N Kłos, C Knee, S R Knight, M A Koc, M A Koç, A F Kocaay, P Koggoh, D Koike, G Kokkinos, P Kokoropoulos, C Kolla, V V Kollengode, M Kołomańska, L C Kolongi, D O Komolafe, K Komorowska, S Kondpan, D Kone, A Königsrainer, I Königsrainer, D Konkin, E D F Konlan, M K Konstantinidis, E Kontis, A Kontokostopoulos, K Kontzoglou, N Kopanakis, K C Kordeni, D Korkolis, C Korzin, M L Koschke, J Kosir, J A Košir, T Košir Božič, S Kosna, P Kostoglou, G Kostoulas, M Kotb, N Koter, P Kothari, D Kotsaris, R Kottayasamy Seenivasagam, G Koukoulis, M N Kouliou, K Koumarelas, G Kouraklis, C Kourouniotis, M Kouta, N Kouzakos, P Kowalewski, Y O Koyluoglu, A Kozadinos, I Kozadinos, T Kozonis, R Kpangkpari, F Krämer, C Krautz, W Krawczyk, S Kreid, A Krishna, R Krishna Raj, N Krishnappa, K Kröning, M Kruščica, S Kshirsagar, E Kubiliute, G O Kucuk, V Kudoh, Y Kudryavcev, M Kulimbet, D Kulkarni, S Kulkarni, N Kullab, B Kum, A Kumar, A Kumar, A Kumar, M Kumar, N Kumar, P Kumar, R Kumar, S Kumar, V Kumar, S Kumar Venkatappa, S Kumaran, P Kumassah, U Kumbhar, Ö Küpçüoğlu, D Kurochka, G K Kurtoglu, G M Kurtoglu, G K Kurtoglugk, M Kusiński, J Kutkevičius, A Kutma, F Kuubetersob B. N, T Y Kwan, M Kwon, N Kydonakis, I Kyei, M Kyereh, D Kyeremeh, S Kykalos, H Kynaston, F Kyramargios, A Kyriakidis, M Kyriazi, L Bandareesh, A L'Hostis, D K K Labadah, O Ladlow, L Laface, N Lahmer, N Lahnaoui, A Lai, W K H Lai, M Lainez Escribano, Y Lakdawala, S Lakew, N Lakhanov, P Lal, A K Lala, R Lalanda, N Lalovic, Y H Lam, K Lambri, A Landaluce-Olavarria, I Laopeamthong, A Lapergola, P Lapolla, N Laquatra, P Laranjo, A Lareida, C Larotonda, R Laskowski, C Lategan, S G Laura, S Laura, S Lauricella, A O Lawal, A Lawal, A Lawal, T A Lawal, S Lawday, M Lazo Ramírez, H Le Roux, C Leal Ferrandis, H Lederhuber, D N Lee, G Lee, K Lee, L D Lee, S Lee, K Lehmann, N Lekiashvili, L Lely, A Leon-Del-Angel, F J León Frutos, C León-Espinoza, N N Leonelle Lore, J Leong, R Lertnamvongwan, C Leung, P Leungon, P Levíček, A Li, C Li, M R Li Valencia, R Liang, S C Liapis, A Liaquat, L Licari, A Licciardello, H Lidbetter, M Lie, L Liepa, M Lill, A Lim, A Lim, C K Lim, J H Lim, O Lima Azurdia, P Limani, C Limas, A Lin, V Lin, E P Lincango, J Lindert, N Lindi, J Linker, C Lionel, O Lisin, A Litchinko, D Liu, V Liu, V Lizzi, T Lo, L B Lo Piccolo, E Locci, M Lodha, N Lodhi, M W Löffler, A Loizou, S N Loke, A Lombardero, T Longkumer, R Lopatriello, B Lopes Patrício, N E López Bernal, E López-Negrete Cueto, Z Lorenc, E Lori, M Lotfy, A R Lourenço, I M Lourenço, B Louro, A Lovi, D Lowen, E E Lozada Hernandez, Y Lu, M Lucchini, F Lucero, S Luciano, G Luglio, A Lukáč, H Lule, B Lulham-Robinson, E Lun, E W Y Lun, R Lunevicius, C I Lupercio Figueroa, A Luther, K K Luthra, M Luthra, A Luzzi, P Lykoudis, D Lytras, M Srikanth, L M Mheidat, M S Yashas, T M. Abubasheer, D M. Awad, B M. J. Alhaj, M M. Nathan, S M. Udwan, M Maak, D Maan, B Maanikuu, T Mabogoane, W Mabood, C Macedo Cardoso De Oliveira, N Machairas, A Mačiulaitytė, R Mackay, J Mackenzie, S Mackenzie, M Maclean, M E M Madany, R Madi, Y Maestre González, F Maffei, F Maffeis, R Magarini, A Maghrabi, S Magrhi, B Maguire, M Mahadi, M Mahafdah, O R Mahafdah, H Mahajna, C Mahakalkar, K Maharaj, I Maharem, N Maharjan, S Mahdi, S N Mahendra, A Mahendran, V Mahendravarman, A Maher, S Mahfoud, N Mahmood, F Mahmoud, F Mahnic, M Mahran, J V Maida, T Majed, L Majerčák, J G Makama, D Makary, S T Makkai-Popa, Y Maktabi, R Malatesti, S Malek, S S Malhi, A A Malik, N A Malik, H Malkawi, Y Malki, D Mallik, G Maltinti, S Mamduh, G Mammolo, M Manangi, F Manasci, D K Manatakis, V Manchev, A Manetti, M Manfredini, T Manickam, Y Manickchund, M Manigrasso, P Manikis, P Manjunath, L Manoj Joshua, M Mansoor Iqbal, A Mansour, A A Mansour, E Mansour, L Mansour, M Mansour, O Mansour, S Mansour, S V Manzoor, B Mao, S Maqbool, S Maqsood, A Maqsood-Shah, C Marafante, A Marano, A Maraqa, S Marawu, M K Marawy, M Marcianò, N Marcos, M M A Marei, A Marello, R Mares País, I Margaris, E Margelis, C Margiani, M Mariani, F A N Marin, G M F Marini, A Marinis, V Mariottini, J Maritz, C Markin, B Markowska, R Marlin, G Marom, P Marongiu, A Marouf, I Maroulis, J Marques Antunes, M Marqueta De Salas, L Marquez, E Marra, J Martellucci, E Martí Cuñat, M d M Martí-Ejarque, B Martin, J Martin Fernandez, M P Martin Gimenez, O Martin Sole, L Martinek, L Martinez, M J Martínez, M N Martínez Bareiro, R Martínez Díaz, A Martinez German, G Martínez Izquierdo, J F Martinez Martin Del Campo, M Martorana, J N Marx, S Marzorati, N Marzouqa, M Masaaod, H Masalma, A Masciandaro, Q Mashlah, M Masood, M Masoud, R Masri, S T Massa, D Massaras, D Massaro, G Massarra, P Masseria, I M Matache, O Matar, M Mateu, A R Mateus Loureiro, A Mathew, S Mathew, N N Mathioudakis, Z Matkovic, G Matroud, S Matthiess, M Mattis, M Matyja, N Maulenov, J Mavrek, M Mavri, E Mavrodimitraki, A Maxwell, C Maya, M Maybury, W Mayo, E Mazumdar, R Mazurkiewicz, F Mazzola, A Mazzoni, E Mazzotta, E Mbanzabugabo, J C Mbonicura, R Mcclen, S Mcclintock, B Mcdermott, R McGee, M McGuinness, B Mckay, K McKenzie, J Mcnab-Hand, A Md Yunos, F Medas, M J Medina, S P Meena, S Megdiche, M E A Meghaizerou, S Megna, M Mehanny, M Mehmood, A Mehta, Y Melkamu, M P Mellado Tellez, M Melouane, N Memos, J Mendes, G Mendiola, S Meneghini, B Mengesha, D Mengiste, P Mensah, S Mensah, C Meola, F Mercado Sanchez, M Mercurio, S Meriç, D Merlicco, D Merlini, M Merrakos, M Mesfin, Y Y Metaferia, J Metzger, G Mevognon, S Mewa Kinoo, H Mftah, R Michael, V Michael, N Michalopoulos, J Michel, S Midya, A Miele, M Mietła, A Migdanis, J Mihanovic, S Mikalauskas, D Mikami, I Mikulic, M Milentijević, P J Milewski, L Milic, J Miller, M Milone, H A Mimouni, E Mina, A M Minaya Bravo, A Mingoli, Y Mintz, R Miranda Pera, M B Mirza, D Misca, A Mishra, K K Mishra, T S Mishra, J Miskovic, D Mitchell, A Mitsala, R Mittal, H Miyasika, M Mobolaji-Ojibara, V Modekwe, M M Modolo, I Mogárrio, T G Moges, E Moggia, M Mogiatti, T Mogne, S F Moh Pauzi, B Mohamad, M Mohamad Amin, E Mohamed, O Mohamed, Y Mohamed, Y Mohamed, A Mohamed Ibrahim Mohamed, O Mohamed Mokbel, M Mohamedshafee, M Mohammad, J Mohammad Mohammad, H Mohammadi Sardoo, A Mohammed, A Mohammed, E Mohammed, K M G Mohammed, L Mohammed, M M H Mohammed, S Mohammed, T Mohammed, U Mohammed Bello, F A Mohammed Daoud, M MohammedAli, D Mohanty, M S Mohd Shah, A Mohsen, G Moitzi, M Mokhtar, Á Molero, C Molewa, S Molfino, J D Molina Marin, M Molteni, M Momoh, E Monati, A Moncy, I Mondi, J Mondino, F A Monib, L Moniz, S Monkhouse, M Montagna, E Montalbán Martínez, E Montanari, O Montaño Angeles, A G Montaser, L Monteiro, J Montero García, S Montisci, D Montwedi, P Moodley, L Moolla, A Morad, J Morales, S Morales, J Moravik, L Moreira, L Morelli, A Morello, M Moreno Gijon, M A Moreno Gonzales, C Moreno-Licea, O Morgan, K Mori, V Morinelli, D Moris, M Morjan, M T Morna, D Moro-Valdezate, N Morricone, N Mosbah, C Mosca, V Mosca, S Moschella, N Mosleh, M S Mosquera Paz, A Mostafa, M Mostafa, M Mostafa, S Mostafa Yassin, T Motiwala, A Mouffokes, R Moukarzel, M B Mourato, A Mourtzouni, V Mousafeiris, H B Moya- Ambriz, M Mozel, C Mpirimbanyi, I Mrazkova, M R Mslmani, M Mubarak, I Mubezi, A B Muhammad, S Muhammad, D A Muhie, J Mühlhäusser, M Muir, L Mukamazera, F Mulita, S Mungo, D Muñoz, M E Muñoz Fernández, N Muñoz Montes, A M Muntaka, R Munyaneza, G Munzi, M Muragi, V Muralidharan, M Muresan, M E Muriel, E M A Murphy, I Murshed, A Murtada, V Murzi, N Musawa, K Muscat, D Muschitiello, Z Musilová, A Mustafa, A Mustafa, B Mustapha, A B Muthunayagam, D Mutter, D Muyenzi, A Mwanjoka, A M Myintmo, Y Myla, I C Mylona, N Naabo, M H Nabhan, A Nabil, E A Nachelleh, A Nada, T Nadeem, I Nadi, T N Nagwamutse, G Nagy, K Nahar, E Naidoo, R Naidoo, U Naidoo, S Naidu, A Nair, S Nair, V V Nair, J Najajra, P Nambala, K Nanayakkara, M Nandasena, M A Ñañez, J Napoli, N Narain, J R Naranjo Fernández, C Narayan, M Narayanan, B Nardo, A E Narin, M Nasani, P R Nashidengo, I Nasser, K Nassim, C Nastos, F Natali, R Nataraja, G Natchagande, M Natey, R Nathani, A Natili, V Naunova, A M Nava Franco, A Navarrete-Peón, Ł Nawacki, A Nawawi, S Nayak, F Nazareth, E Ndizeye, O Ndizeye, B Neall, I Negoi, V M Negoita, C N Nelly Rosine, S Neogi, Y Nerabani, I Neri, R Nesco, L C Nespoli, A Neves, V Neykov, J Ng, S Ng, S H M Ng, V Ng, J Ng-Kamstra, A Nguyen, A Niazi, G D Nibogora, C Nicholson, A Nidali, M Nieto Galvan, K Nieto Yrigoin, K Nieuwenhuys, N S Nik Burhanuddin, N Nikam, M Nikberg, A Nikitaras, A I Nikolaou, C Nikolaou, V Nikolaou, B Nimako, C Nimbona, A Ningi, G E Nita, A F Nixon Fulli, L Niyidukunda, I Njere, M Nkogatse, M Nkoronko, G Nkunguzi, M Nnabagulanyi, J Nnoli, M Nofal, S Nofal, K Noguchi, M Noguez Castillo, R Noor, I Norman, M Nortey, O Nouhail, H Novák, S Novello, C Ntagkas, N Ntanzi, K Ntatsis, P Ntem, C K Ntow-Boahen, G Ntwari, O G Nubi, E G Nubi Mohamed, M Nunes, M Nunes Luís, H Nuñez Del Barrio, A Nuñez Venzor, C M Nuño-Guzmán, S E Nwabuoku, C Nwachukwu, N Nwafulume, E I Nwangwu, C Nwanmah, N Nwanne, C Nwegbu, C Nwokoro, C Nwosu, C D Nwosu, J Nyamekye-Baidoo, C Nyampinga, F Nyarko, M Nycz, M Nyirenda, R Nyirenda, P C Nze Obiang, I C Nzenwa, E O'Connell, E O'Neill, M Obaid, A Obbeng, I Obianyo, E A Obiesie, N K A Obuobi, H O Odah Bashi, Z Odeh, K E Oderoha, V Odigie, K B Oduro-Boateng, E O Ofori, J Ofori, D Ofosuhene, I Ogundele, H Ogundipe, S Ogunlade, O Ogunsua, K Oğur, S Ohri, A Ojewuyi, O Ojewuyi, A Ojo, M Ojo, O Ojo, A Oke, M Okechukwu, J Okei, I Okoro, O A Okoye, O Okrah, M Okudero, A I Okunlola, A Olabode, O Olajide, O Olajumoke, F Olaniru, J Olaogun, O Olasehinde, R Olatunji, S Oliveira, M Olivos, M D M Olmedo Reinoso, T Olobatoke, N Oloko, S Olori, J Olorunfunmi, A Olsen, K Oluchukwu, A M Olugbami, O Oluseye, O Oluyemi, D Omar, M Omar, M Omar, S Omar, U Omara, M Omer, N Omer, D Ommi, O Omoike, L Omomeji, P Omwansa, M Oncel, F Ondago, H M A Oneizah, C S Ong, A Ongaro, V Ongil Rodríguez, F Oni, C Onuoha, C Onwuzu, L Onyebulu, C Ónyeka, K Onyekachi, K Ooi, R Ooi, A N Oommen, A Oosterkamp, F Opoku Twene, A Opoku-Agyapong, A Oppong, J Oppong, R Oppong, R Oppong-Amoah, Y Orabi, I Orji, G Orlando, J E Orozco Navarro, J Orozco-Perez, M Ortega Escudero, L Ortega Lechuga, Z Orzeszko, O Osagie, Z Osama, E Osaze, N Oscar, D B Osei, E O Osei, D Osifo, C Osime, F Osman, I Osman, M Osman, O Oso, V Osoka, M Osso, P Ostruszka, C Osuigwe, O Osunlusi, S Otho, J Otote, A Ottaiano, L Ottaviani, A Ouachhou, N Ouachou, Y Ouadi, S Oum, A Ousseini, P Owens, O Owolanke, F Owusu, G Owusu, B Owusu Ansah, A Oyedele, A A A Oyelekan, N Oyelowo, T Oyeyemi, H Oyinlola, O Oyinloye, M A Oyortey, D Ozal, İ H Özata, A Özcan, B Özcan, M F Ozcelik, K Özdoğan, O F Ozkan, C Özkan, A Ozkomec, E Ozoran, H Ozsahin, M S Ozsoy, A M Öztürk, S Öztürk, G Ozyuksel, P Sagar, P S Induchoodan, M Pacilli, M Pacilli, T Padioti, N Paez, M Pagani, E Page-Taylor, D Paglione, N Pahuja, H S Pahwa, D Pais, R Pajtak, A K Pal, R M Palacios Huatuco, N Palamara, M Palas, P Palazon Bellver, D A Palma Portillo, M Palmeri, F Palmieri, G Palmieri, D Palmisano, G Palomba, F Palomino Escalante, P Palumbo, A Panagakis, A Panagidis, A Panagopoulos, D Panagopoulos, P Panahi, L Pánči, V Panduro-Correa, S Pandya, H Panga, C Panis, M Pannullo, D A Pantoja Pachajoa, I Papaconstantinou, N Papadogianni, M Papadoliopoulou, A Papadopoulos, V Papadopoulos, D Papageorgouli, E Papamattheou, M Papamichail, C Papazacharias, X Papazarkadas, G Pappas Gogos, P Paraskeva, J Paredes, L Paredes, M Park, N Parker, R Parker, T Parker, C Parmar, F Q Parray, A Parseliunas, I Parwaiz, G Pascarella, F R Pascual, A Paspala, N Pasqua, C Pastás, A Patel, P Patel, P Patel, P Patel, S Paterna-Lopez, L Paterson, M Pathak, S R Pathan, D C Patiño García, S Patrocínio, R Patrone, D Patterson, B Paul, N Paul Ambrose, N P Paul Sigamony, K Pavlopoulos, G Pavone, S Payyanur Thotan, A Peckham-Cooper, F Pederiva, H Pehlivan, M Pejović, L Á Pelayo Orozco, J B Pellecer Cano, M Pellen, G A Pellicano', G Pellino, L G Peña Balboa, M J Peña Soria, J Pereira-Macedo, M Perera, R Perera, M Perez, R Perez, Y Perez, C J Pérez-Padrón, H D J Pérez Baca, D A Pérez Muñoz, C J Perez Rivera, R H Perez-Soto, M R Peris, K Perivoliotis, A Perja, M Perkins, G Peros, S Perretta, D Pertile, A L Pesce, A Pesce, L Petashvili, C T Petcu, B Peter, M Peter, F Peters, L Peters, N Petrarota, K Petrenko, J Petric, T Petropoulou, A Petrungaro, W Petrushnko, B Phakathi, M Pham, T V Pham, O Pheiffer, R Philip Sridhar, M Philipp, F Philips, D Phom, M G Piacentini, M Piccino, S A Piccioni, M C Pignanelli, G Pignata, D Pignatelli, A Pikarsky, A Pilavas, A Pilavas, T Pilia, A Pillai, G Pillai, V Pillay, S Pimentel Morais, R Pinheiro Duque, A M Pinheiro Pereira, J Pinho, A Pinto, B Pinto, I Pipia, C Piras, D Pironi, M Pisano, M Pisano, A Pisanu, G Pisarevi, A Pita, M Pitiakoudis, I Pius Ogolekwu, J Pizarro Lozano, A Plastiras, H Pleass, X Y Po, M Podda, L Poggi, L Poggi, G Poillucci, S Polat, A Polatbekov, A Police, D Politis, R Polo, K Polychronopoulos, E Pontecorvi, O Popoola, F Porcelli, M Portelli, E Potenza, G E Poto, G Pou, E Poulios, M Pourfridoni, D Powell, H K Prabhakar, P S Prabhu, L Pramod, S V Arun Prasath, J Prat-Ortells, N Pratas, A L Preto Barreira, D Prevezanos, F Prieto La Noire, P Probst, G Procaccini, M P Proclamà, D Proud, M Prudhomme, O Pryer, M Pryt, J Psaila, I Psilopatis, S Puddu, C Pun, A Punzengruber, A Qasem, H Qayum, J Quansah, M Quante, S Quartarone, M Quaunine, F W Quayson, A Quddus, C Quintela, K Quispe De La Roca, S Quoy, A U Qureshi, Z Qureshi, S Qwyder, B R Budihal, I R Fakhradiyev, R Rabah, N Rabai, D Rabaia, R Raddad, B Radja, R Radojković, G Rados, R Radwan, S Rafiq, M N Rafique, A Ragab, L Ragazzini, A Ragheb, R Raghunath, K Raghuwanshi, C F Rahantasoa Finaritra, M Raheem, R Raheem Attallah Al_obaidy, A Raheja, A Rahman, G A Rahman, K R Rahman, D Rahme, L Rai, N Raiq, N Rajab, K Rajaratnam, S Rajendra, D Rajput, M J Rakotonaivo, A Rakotondrainibe, I Rakshit, I Rakvin, F Ramalho De Almeida, M Ramazanov, R Ramchandani, A Ramírez Beas, R A Ramirez Calas, S Ramjit, N N Ramli, A N Ramly, A Ramos Bonilla, L Rampersad, R R Ranadive, B Ranjous, M Ransome, C Raphael, A Rashad Temerik, A Rashid, T Rashid, F Rasoaherinomenjanahary, N Rasool, V Ratheesh, J Rathod, K J Rathod, D Rattray, F Rauf, S Raul, N Rawal, T Rawther, O Ray, M Rayzah, A Raza, Y M Razafimandimby, J B Razafindrahita, M Razaq, S Razaq, A Razzore, F Ré, L A Rea Bocanegra, M Reda, K M R Reddy, S M Reddy, R Redkar, E Redondo, F J Redondo Calvo, M Redynk, R Refaie, D Rega, P Rego Ponte, W U Rehman, B Rehmani, M Reia, T Reichelt, E Reid, H Reilly, J Reilly, D Reim, M Reis, E Reitano, K Rekouna, A Rencuzogullari, L Rende, S Rennie, M Rennis, N Renovat, L Resca, B Révérien, D E Reyes Rodríguez, D H Riazhussain, M Riba Martínez, J Ribeiro, J Ribeiro, R Ribeiro, R Ribeiro Dias, D Ribero, C Riboni, J Ricardo, V Ricchiuti, G Riccioli, H E Rice, K Richetti, K Richter, D Ridder, P Riedl, C Righetti, J Ringers, C Rio Ferreira, L Ripamonti, R Ripoll I Palmés, F Ris, T Risteski, M Rius, P Riva, R Rivas, U Rivolta, R Roberts, Z Robertson, B Robertson-Jones, A Robin Valle De Lersundi, L Robine-Durnell, S J A Robinson, S Robinson, R J Robitsek, M S Rodha, J L Rodicio Miravalles, C S Rodrigues, L Rodrigues Madeira, L Rodríguez Gómez, A Rodriguez Gonzalez, M Rodriguez-Lopez, M Rodríguez-Ordoñez, R Rodríguez-Uria, A Rojas, J Rolinger, C Rolo Santos, E Romairone, J Roman, C F Roman Ortega, S Romano, A Romero De Diego, G Romero Reyna, E V Romo Ascencio, F D Romo Rosales, U Ronellenfitsch, F Roodt, B Roopavathana. S, D Rosado, A Roslani, L Rossi, M Rottoli, N Roukounakis, A Roumieh, E Roussos, M Roussos, F Roviello, P Royo Dachary, L Rubin, R Ruccella, F Rudisch, R Rüdrich, M J Rueda Medécigo, F Ruescas, M Ruffoli, J P Rugambwa, V A Ruiz López, M Ruiz Soriano, E Ruiz-Daum, L Ruiz-Villa, D Rukavina, C Rukundo, I S Russo, N S.Bayleyegn, M M Saad, A Saba, A Sabbah, Q Sabbah, S Sabbatini, M Sabboh, H Sabir, T Sabra, M Sabri Massadi, A Sabry, M D Sacdalan, M Sadek, I Sadiq, M A Sadiq, M H Sadiq, H Sagar, R Sagoe, L Sahan, H Said, M Said, S Said, M Saidani, M Saifi, N Saini, A Sainz Lete, E Saitoglou, Y Sakaray, P Sakarellos, A Sakr, M Salah, M Salah, O Salako, A Salam, S Salama, G Salamone, P Salas Núñez, A Salazar, T A Salazar-Lorenzana, M Saleem, A Saleh, M Saleh, M Saleh Khatab, G Saletta, N Salgadoe, M Salibašić, S Salido, R Salim, S Salim, A A Salinas Barragan, S Salindera, D Salinovic, E Salloum, S Samadi, E Samara, D Samardali, S Samardali, Y Samer Morsy, L Samison, M Sammut, M Sammut, C Samojeden, D Sampanis, R Sampietro, M San, A Sanad, M A Sánchez Audelo, N Sánchez Fuentes, A Sanchez Gallego, A Sánchez Gollarte, M Sánchez Ramirez, M Sánchez-Rubio, A I Sánchez-Terán, P Sancho Pardo, S A Sani, A N Sanli, A S T Sanon, A F Sanon, A Sanou, R Santana Ortiz, M Santarelli, L Sante Serna, M Santillan, G Santos, I Santos, M Santos, R Santos Pereira, W Santucci, S Sanz, A Sapmaz, M W Saqib, A Sarafi, A Sarakatsanos, B Sarang, M Sarda, O Sarhan, A Saridaki, A C Sarı, M A K Sarker, G Sarp, Y Sarpong, S H Sarwary, D Sasia, R Sato, S Saudí-Moro, J Saunders, C Saviello, K A Sawaftah, M A Sawaftah, M A M Sawaftah, M N Sawas, G Saxena, R Saxena, R Sayad, A Scacchi, L Scaravilli, M Scheiterle, A Scheiwiller, J Schembri Higgans, N Schiavon, L Schiavone, S Schimpke, D Schippers, V Schirinzi, S Schirone, D Schizas, E Schmidt, R Schmidt-Branden, M A Schneider, M Schön, M Schüler, G Scialandrone, R Scola, F Scolari, M Scopelliti, M Scortechini, B Scotto, D Scotto Di Carlo, M Scricciolo, Y Seada, A Sebai, M Sebastián-Mendoza, J Secchi, N Seeger, C Seet, S Segnitome, B Sehgal, A S Seidu, C Seiler, M I Seixo, M E Seker, S Selim, S Sellahewa, F Selvaggi, C Semeraro, Y K Şen, K J Senanayake, J Senavirathna, C Seneza, R Senin, Z Şenol, I Separovic, E Sepúlveda, L Sequi, A Serao, W Serednicki, I Serfontein, M Şermet, V Serna-Alarcon, I Serrano, P Serrano Méndez, M Sersarah, B K Seshie, M K Sethi, R Sethi, M Seto, E Setsoafia, S Severi, K Sevnaran, G Seyfu, J Seyi-Olajide, G Sgarzini, Z Shabello, A Shabkah, A Shah, Z A Shah, I Shahbaz, A Shaheen, H H Shahid, M Shahid, M Shakhshir, M Shalaby, M Shalaby, M Shalkamy, H Shames, K A Shamiyah, L Shammas, M Shams, G Shamsi, A Shamsutdinova, A Shanker, S Shanker, A Shanmugalingam, B Sharaf Eldin, I Shariff, D Sharl Ajami, A Sharma, A Sharma, K Sharma, M Sharma, N Sharma, N Sharma, R D Sharma, R Sharma, V Sharma, A Sharp, A A Sharq, L Shawesh, I Shehadeh, N Shehata, C Shelton, K Shemyatovsky, D Shen, R Shen, H Sherif Farouk Ahmed Hassan, M Sheriff, A A Sheshe, E Shestakov, P Shimbulu, C Shiraishi Zapata, S Shittu, P Shivashankar, S Shivashankar Chikkanayakanahalli, K Shodunke, A Shoker, T T Sholadoye, M D Shoshkova, K Shreyas, P Shukla, N Shulman, K Shwail, A Shweiki, I Siannis, S Sibiya, J Siby, G Sica, M Siddique, T Sidiropoulos, A Sidorova, J Sidorovskaia, V Siepaal, S Sierra, E Signaroli, E Silanos, A Silva, C Silva, D Silva, R Silva Borges, F J Silva Rivera, D W Silva-Cano, S Silvestre, V Silvestri, K K Sim, B Sime, S Simeonidis, J Simoes, L Simonelli, G Singer, A Singh, D Singh, G Singh, G Singh, I Singh, K Singh, M Singh, M Singh, M Singh, M P Singh, S K Singh, S Singh, S Singh, T P Singh, V Singh, A Sinha, A Sinha, R Sinha, A Sinjab, L Siragusa, D Siriwardena, A C Sironi, J Siu, J Skaff, A Skarpas, H Škiljo, A Skreka, G Skroubis, M Slavchev, C Smart, G Smith, K Smith, S Smith, R L Smolinski Kurek, A Smyrnis, C Soares-Aquino, C Soddu, S Soelling, F Soggiu, R Soglonou, M Sohn, R Sokratous, L Solaini, N Soldo, R Solecki, F Solimene, R Soliva Domínguez, M Solkar, N Solomon, E Somuncu, A A Sonkar, R E Sönmez, R E Sönmez, T Soric, D Soriero, P Sornoza, S Sorrenti, C Sorrentino, G Sorrentino, E D Sosa Ferreira, D A Sosa Méndez, M A Sotiriou, M Sotiropoulou, S Soto Schütte, Ó A Soublett Rivas, I Sougkas, A Soumpasis, R Souto, E Soyer Güldoğan, E Spalice, F Spanos, E Spanoudakis, A Sparke, E Spartalis, M Spartalis, K Spassov, A Spaziani, R Spence, B Sperotto, N Spiteri, I Spyridakis, N Srinivas, S Srishankar, M Srour, S Staight, P Stamopoulos, N Stamos, H Stark, F Stavratis, G A Stavrou, G Steenkamp, C Stefanou, M Steffani, F Stefou, T Stefura, L Stella, C Stennard, N Steve, S Stevens, M Stjepanovic, s Stock, D Stoian, A Stollberg, S Stonelake, S Strachan, A Strumia, A A Suárez Álvarez, D Subasinghe, S Subbiah Nagaraj, A Subocius, R Sucher, S Sucu, R Sugair, A Sukhdev Jadhav, P Sukhvibul, A Sukumar, R Sulce, M Sulciner, F Suldrup, I Suleiman, I E Suleiman, R Suleiman, J Suleiman Hadidi, M Suleman, J Suliman, M A Suliman, F Sulo, A Sultana, A Sumbaev, A Sundaram, G Sundaram Venkatesan, S Sundaramurthy, S Sundararajan, S Surendran, P Suresh, S Suresh, A L Surkitt, A Suroy, J Sutcliffe, V Sutharshan, B Sv, L Sweidan, M Święch, A N Syed, A Syllaios, E Synekidou, A Szpytko, M Szura, G Szydlo Shein, D T. Enti, P Taah-Amoako, M Taamreh, N Tabatabaian, M Tabaza, A J Tabiim, J Tabora-Zepeda, M Taeme, L Tafesh, M Tageldin, K Taghavi, L Taglietti, I Tagreda, H Taher, A Tahir, M A Tahir, W Tahir, F Tahir Lwdie, J Taiwo, A Talabi, N Talat, L Talé-Rosales, L Tallon-Aguilar, D P Talreja, K Tambudze, N Tamini, Z F Tamou, E C Tampaki, A Tampakis, E Tan, S Tan, M Tanal, Y Tanas, I Tanase, M Tanashat, Y I Tandoğan, C Tandup, P Tang, A Tansawet, L Tapia Moral, G Tarantino, M F Tarar, A Tariq, X Tarrado, N Tartaglia, N Tasis, K Tata, F Tauheed, D Tavares, J A Tavares-Ortega, S Tawfik, M Tayyab, N Techapongsatorn, S Techapongsatorn, T Techapongsatorn, J S T Tefay, P S Tekam Wadje, H Telci, M Tello Jimenez, V Ten, R A Tenfen Carneiro, F Terefe, M Teressa, E D Terzi, I Tesfahun, A Teshome Sahilemariam, H Tetley, S P B Thalgaspitiya, P Thambi, A Thanasa, T Theivendrampillai, S A Theivendran, V Themelidi, A Theochari, M Theodoridou, K Theodoropoulou, T Theodosopoulos, N Theophile, L Theuil, R S Thind, A Thomas, P A Thomas, C Thrasyvoulou, S I Thuraisamy Sarma, A Tibude, F Tierenye, I Tierris, S Timmalah, S Tingle, B Tinoco, F Tirelli, A Tirkey, R Titcombe, J Tk, S R Tobome, F Tofani, B Togay, A C S Toi, E Tokidis, M Tokocin, M A Tolani, E A Toma, A Tomazic, M Tomlinson, S Tontus, T Tony V, S Toprak, M M S Tora, A Torbey, L V Torres Bavestrello, E M Torres De Anda, S T Torres Rodríguez, A Torroella, Y Tosun, Z Toutounji, N Trabulsi, P Trakosari, N Tran, G Travaglini, S L Trejo Ramos, M Trejo-Avila, T D B Tridip, A Trif, A Trigos Díaz, M Trindade, P Trinity, R Tripathi, F P Tropeano, S A Trujillo Ponce, I Tsakiridis, N Tsakiridis, C Tsalikidis, N Tsantikos, V Tsaousis, S Tsatsos, S Tsatsu, P Tschann, A Tsechpenakis, V Tselepidis, A Tsiaka, D Tsiardas, F Tsige, T Tsirlis, M Tsopozidi, S M Tsoti, G Tsoulfas, M Tsuruta, L Tulinsky, H Tümer, R Tummon, R Tumolo, E Tunçcan, C Tung, K Y Türker, S Turkmani, T B Türkmen, A Tursunovic, R Tutino, M Tutton, E Tuzuner, A Tvaladze, A Twumasi, H Tyagi, E Tyurina, P Ubiali, R Ubiñas, C Uche, J Ugwu, I Ugwueke, C Ugwunne, T Uiyapat, J J Ulloa Robles, H Ulman, S S Uludağ, N Umar, C W Un, U Una, E Unal, H Unwin, T K Uprak, K Urbańska, G Urdang, M Usama, M Usman Malik, N Uttam, C Uwakunda, M S Uyanik, D S Uymaz, J Uys, A M Uysal, M Y Uzunoglu, C Vacca, X Vagena, M Vailas, J Vaitekūnas, R Vakil, F Valente Costa Pinto, J I Valenzuela, Z Valera Sanchez, S Valeri, T Valizadeh Elizeh, L Van Den Hil, M Van Der Colf, J Van Niekerk, B Van Zyl, D Vardakostas, A Varga, C Varghese, R Varshney, M Vasconcelos, Y Vashishth, S Vasquez, P Vásquez, C Vasquez Maya, F Vassallo, P Vassiliu, J Vaz, D Vaz Acosta, R Vaz Pereira, A Vazquez Fernandez, A Vazquez Melero, S J Vázquez-Sánchez, S Vederaki, A Veira, J Velkoski, F Velluti, S Vembar, L Venclauskas, D Venskutonis, P Venturelli, C Vera Mansilla, D Verdi, S S Verhage, S Vernadakis, M Veroux, G Verras, L Verre, S Vertaldi, N A Ververidis, A Vezakis, A Viacava, M Vicario Bravo, A Victor, I Vidić, B Vieira, J P Vieira De Sousa, A Vig, A Vilar, R Villalobos Mori, S A Villeda, J F Viñas, G R Viscido, D Visconti, C Vitiello, N Vlachakos, P Vladova, E Volpin, K Voon, V Vougas, F Vovola, A Vricheva, A Vu, J Vu, D Vukosav, K Waddell, P Waghchoure, H Wain, G Waiyaki, M Walędziak, D Waleed, A Walfor, D S Walia, P Walker, E Wallner, J Walshaw, B Wang, J Wang, J Wang, R Wani, S H Waqar, S Ward, M Waseem, I Wasiu, A Wassouf, M Watson, C Weadick, G F Weber, M Weber, O Webster, M West, O Whitehurst, M Wichmann, D Wickramarathna, D Wickramasinghe, F Wiese, C Wiesner, W Wijenayake, K Wijesinghe, M Wikar, A Wilkins, A Williams, B Williams, O M Williams, H Willy-Chidire, M Wilson, P Wilson, M Windsouri, U Wirth, T Wojewoda, J Woleský, V Wolfschluckner, E Wong, J Wong, Z Y Wong, S T Workineh, M Worku, S Wrenn, D Wright, W Wysocki, H Xiao, B Yacoub, H Yaghmour, A Yahaya, M Yahaya, M Yahmad, A Yakubu, O Yalkın, K Yalley, W Yang, C Yanowsky-Gonzalez, G Yanowsky-Reyes, G Yarovenko, A C Yaşar, M Yashar, L Yasin, M M Yassin, S Yassin, E Yavuz, I E Yavuz, P Yeboah Owusu, E M T Yenli, E Yeshialem, A Yeshitila, A B Yevide, W T Yew, E Yhoshu, A Yiallourou, M Yigah, B Yigit, D Yigit, D Yiğit, H Yigitbas, S Yigman, E O Yildirim, A Yildiz, S Yilmaz, A Yingess, M Yinusa, S Yılmaz, T E Yılmaz, G Yohanna Abrak, H Yonekura, J Yorke, I Yotsov, T Yotsov, J You, E Younes, A Younis, H Younus, R Yousef Yassin, N Yousefzadeh Kandevani, M M T Youssef, E Yousuf, K K A Yu, P Yuide, S Yumurtacilar, H Yunes, M A Yunus, B Yunusa, S A Yunusa, C Yurttas, S Yussif, M Zaazou, B Żaczek, A Zafar, M Zahed Abdalla, Z A Zaher, M Zahran, H Zaigham, M Zaigham, H H Z Zaini, J K Zajac, A D Zakaria, K Zakkas, A Zamareh, M Zambon, M Zammit Vincenti, N Zampitis, Ö P Zanbak Mutlu, C Zandonella, G Zanni, G Zanus, F Zapata, C Zapata Syro, K Zapsalis, A Zarafidou, M Á Zaragoza Mendieta, M F Zarate Casas, M Zawadzki, G Zaza, L Zeng, A Zerbinati, D Zerbo, W A Zerefa, G Zeringa, R Zerna Encalada, I Zerrouq, J C Zevallos-Quiroz, E Zhang, S Zhang, J Zhu, E Zidan, P Zimmermann, M Zounon, S Zourntou, A Zubillaga-Mares, V Zucchini, V Žufić, D Zuikyte, D Zulian, Y Zwain, H Zwaraa

## Introduction

Technological advancement is important to improve healthcare quality and safety, especially in surgery^1^. For patients with an inguinal hernia, mesh and minimally invasive surgery are the two main technologies that have improved healthcare quality and safety^2,3^. The use of mesh is proven to reduce recurrence^4,5^. This avoids the need for further repairs, which are technically more challenging and have a higher risk for patients^6^. The use of minimally invasive surgery has proven advantages in bilateral hernias and in female patients^2,3^ and is recommended in unilateral repair where appropriate expertise is available^2,3^.

Access to these technologies and the expertise required are not widely or equitably distributed at a global level. As it is the case for other technologies, countries in the Global South have more limited access^1^. At the same time, in this part of the globe, there is a higher prevalence and a higher burden of disease associated with inguinal hernias^7^. Several barriers to implementation in the Global South have been identified previously, including costs, distribution, and training^8,9^. To overcome these, studies reporting the use of mesh based on mosquito net mesh and evaluating training programmes have been conducted^10,11^. With these efforts and with global investment in new technologies and the expansion of existing technologies, it was expected that there would be an increase in their use in low–middle-income countries. Data assessing this variability have not been collected in a standardized way and are usually reported from single-country or single-region studies^5,12^. Therefore, identification of areas where improvement is most needed will be key to better inform policymakers.

The overarching aim of this study was to evaluate access to technologies that are relevant to the treatment of inguinal hernia patients to identify the areas where improvement is needed. Therefore, the primary aim of this study was to evaluate the use of mesh and predictors of mesh use in elective inguinal hernia repairs and the secondary aims of this study were to evaluate the use of minimally invasive surgery and predictors of minimally invasive surgery use and to evaluate the safety associated with the use of mesh and the use of minimally invasive surgery.

## Methods

### Study design

This was a pre-planned analysis of an international, multicentre, prospective cohort study of patients undergoing inguinal hernia surgery. Routine and anonymized data were collected and no changes in patient care were made. The study protocol is publicly available (globalsurgeryunit.org/clinical-trials-holding-page/hippo) and was registered in ClinicalTrials.gov (NCT05748886). Approvals were obtained by local principal investigators in each hospital taking part, according to local and national regulations. This study is reported in line with STROBE guidelines^13^.

### Inclusion and exclusion criteria

Any hospital performing inguinal hernia repair was eligible to take part. Each participating hospital identified consecutive patients undergoing primary inguinal hernia repair as the main procedure during a 4-week inclusion window between 30 January and 21 May 2023. Adult patients, defined as older than 16 years, undergoing elective primary inguinal repair were included. Patients operated on via midline incision or converted to midline incision were excluded, considering the complexity inherent to this approach.

### Outcome definitions

The use of mesh in open surgery was defined as the primary outcome and was compared across the different income groups, as defined by the World Bank. The use of minimally invasive surgery and complications at 30 days were secondary outcomes. Minimally invasive surgery included both laparoendoscopic and robotic approaches and was defined as per intention-to-treat, therefore converted surgeries to open were included in this group. Postoperative complications were defined according to the Clavien–Dindo classification and these data were collected at 30 days after surgery^14^. To comprehensively evaluate postoperative complications, surgical-site infection rates and reoperation rates (mapped to surgical approach and use of mesh) were also collected at 30 days after surgery.

### Data management

Data were collected and stored online using a secure server running the Research Electronic Data Capture (REDCap) web application^15^. The service was managed by the Global Surgery REDCap system hosted at the University of Birmingham, Birmingham, UK. Its security was governed by the policies of the University of Birmingham. Each collaborator involved in data collection was identified and registered by the hospital lead and received personal login details. This allowed secure data entry and storage in REDCap.

### Data validation

The data collection methodology was validated previously, in terms of case ascertainment and data accuracy^16,17^. The hospital lead had access to the data entered by their team. They were responsible for data accuracy and data completeness collected and uploaded from their site. The data were checked centrally and when there were missing data or invalid data, the hospital lead was contacted to complete and correct the data entered. After this, participating hospitals with data completeness less than 95% were excluded.

### Sample size

There was no formal sample size calculation for the analysis proposed and all eligible patients were included. To ensure global generalizability of the results and to justify the resources put into the study, a minimum number of 300 centres contributing patient-level data from 70 countries was estimated, based on previous cohort studies (that is GlobalSurg and COVIDSurg studies)^16,17^. Assuming an average of 30 patients per centre, a minimum sample size of 10 000 patients was predicted. Assuming that the prevalence of mesh use ranges between 70% and 95%, sample size considerations for building a prediction model showed that approximately 2500 subjects would be required to build a model with 7 predictor variables, a prevalence of 95%, and a C-statistic of 0.7 (see *Table S1* for full details)^18^. Sample sizes were estimated using the *pmsampsize* command in Stata, version 18.0 (StataCorp).

### Statistical analysis

Data were mapped to country income groups, defined according to the World Bank (low-income countries, lower-middle-income countries, upper-middle income countries, and high-income countries), as their importance in relation to healthcare access, safety, and quality has been widely recognized^6^.

Continuous non-normally distributed hospital-, patient-, and intraoperative-related variables are presented as median (interquartile range (i.q.r.)) values, whereas categorical variables are presented as frequencies and percentages. The use of mesh and minimally invasive surgery are presented as frequencies and rates across income groups. Postoperative complications, surgical-site infection, and reoperation are presented as frequencies and rates across surgical approach and mesh use groups.

Multilevel logistic regression models were used to test factors that could be associated with higher mesh use in open surgery and the use of minimally invasive surgery. Plausible hospital and clinical factors agreed by the Study Management Group were considered and hospital was included as a random effect. For the above analyses, appropriate model fit diagnostics were checked to confirm that validity and model assumptions were maintained for the data. Categories were collapsed when category event rates were too low to be efficiently included in the model, as was the case for income groups. All statistical analyses were performed using R (R Foundation for Statistical Computing, Vienna, Austria; version 4.0.2). *P* < 0.050 was considered statistically significant.

## Results

### Included patients

**Fig. 1:**
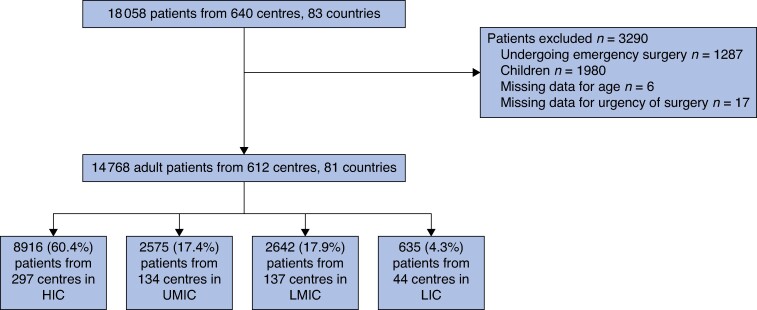
Flow chart of included patients HIC, high-income country; UMIC, upper-middle-income country; LMIC, lower-middle-income country; LIC, low-income country.

Data were collected from 18 058 patients across 640 centres located in 83 countries. For this study, 14 768 adults undergoing elective primary inguinal hernia repair in 612 centres located in 81 countries were included, as shown in *Fig. 1* and *Fig. S1*. Most of them were operated on in high-income countries (60.4%, 8916 of 14 768).

Included patients had a median age of 60 (i.q.r. 47.0–70.0) years (*Table 1*), with an absolute median difference of 10 years between patients operated on in high- and low-income countries. Most patients were male (91.7%, 13 539 of 14 768). Regarding their perioperative risk, most were ASA grade I–II (85.9%, 12 691 of 14 768) and without co-morbidities, which was observed across all income groups. The majority of patients presented with symptomatic hernias (86.2%, 12 729 of 14 768) that were unilateral and with an extension limited to the inguinal region (79.4%, 11 733 of 14 768). Intraoperatively, greater than 90.0% of the operations were classified as clean (14 419 of 14 768). Hernia defect size was variable in all income groups, with defects of 1.5–3 cm being the most reported (40.8%, 6031 of 14 768). Hospitals where these patients were operated on were mostly tertiary-level centres (62.4%, 9126 of 14 768) and their funding was mainly provided by the public sector (86.6%, 12 152 of 14 768) (*Table S2*).

**Table 1:** Preoperative and intraoperative characteristics of adults undergoing elective inguinal hernia repair

	HIC (*n* = 8916)	UMIC (*n* = 2575)	LMIC (*n* = 2642)	LIC (*n* = 635)	Total (*n* = 14 768)
**Age (years)**					
Median (interquartile range)	63.0 (52.0–73.0)	57.0 (45.0–67.0)	52.0 (37.0–63.0)	53.0 (36.0–63.5)	60.0 (47.0–70.0)
**Sex**					
Male	8094 (90.8)	2356 (91.5)	2500 (94.6)	589 (92.8)	13 539 (91.7)
Female	821 (9.2)	219 (8.5)	142 (5.4)	46 (7.2)	1228 (8.3)
Missing, *n*	1	0	0	0	1
**ASA grade**					
I–II	7211 (80.9)	2337 (90.8)	2536 (96.0)	607 (95.6)	12 691 (85.9)
III–V	1645 (18.4)	234 (9.1)	105 (4.0)	21 (3.3)	2005 (13.6)
Not recorded	60 (0.7)	4 (0.2)	1 (0.0)	7 (1.1)	72 (0.5)
Missing, *n*	0	0	0	0	0
**Co-morbidities**					
None	6613 (74.2)	1961 (76.2)	2195 (83.1)	526 (82.8)	11 295 (76.5)
One	1713 (19.2)	491 (19.1)	367 (13.9)	100 (15.7)	2671 (18.1)
Two	456 (5.1)	93 (3.6)	72 (2.7)	9 (1.4)	630 (4.3)
Three or more	130 (1.5)	26 (1.0)	5 (0.2)		161 (1.1)
Missing, *n*	4	3	3	0	10
**Symptoms**					
Asymptomatic	1106 (12.4)	293 (11.4)	580 (22.0)	60 (9.4)	2039 (13.8)
Symptomatic	7810 (87.6)	2282 (88.6)	2062 (78.0)	575 (90.6)	12 729 (86.2)
Missing, *n*	0	0	0	0	0
**Hernia size**					
Limited to inguinal region	7576 (85.0)	1977 (76.8)	1698 (64.3)	482 (75.9)	11 733 (79.4)
Limited to scrotum	1263 (14.2)	569 (22.1)	903 (34.2)	147 (23.1)	2882 (19.5)
Extend to mid-thigh or beyond	77 (0.9)	29 (1.1)	41 (1.6)	6 (0.9)	153 (1.0)
Missing, *n*	0	0	0	0	0
**Hernia site**					
Bilateral	1362 (15.3)	347 (13.5)	386 (14.6)	59 (9.3)	2154 (14.6)
Unilateral	7554 (84.7)	2228 (86.5)	2256 (85.4)	576 (90.7)	12 614 (85.4)
Missing, *n*	0	0	0	0	0
**Hernia defect size (cm)**					
<1.5	1866 (20.9)	456 (17.7)	425 (16.1)	138 (21.7)	2885 (19.5)
1.5–3	3654 (41.0)	1018 (39.5)	1063 (40.2)	296 (46.6)	6031 (40.8)
>3	1804 (20.2)	820 (31.8)	920 (34.8)	175 (27.6)	3719 (25.2)
Not known	1590 (17.8)	281 (10.9)	234 (8.9)	26 (4.1)	2131 (14.4)
Missing, *n*	2	0	0	0	2
**Contamination**					
Clean	8858 (99.3)	2383 (92.5)	2546 (96.4)	632 (99.5)	14 419 (97.6)
Clean-contaminated	53 (0.6)	190	93	3 (0.5)	339 (2.3)
Contaminated	4 (0.0)	2 (0.1)	2 (0.1)	0 (0.0)	8 (0.1)
Dirty	1 (0.0)	0 (0.0)	1 (0.0)	0 (0.0)	2 (0.0)
Missing, *n*	0	0	0	0	0

Values are *n* (%) unless otherwise indicated. HIC, high-income country; UMIC, upper-middle-income country; LMIC, lower-middle-income country; LIC, low-income country.

### Surgical variation and intraoperative outcomes

**Fig. 2:**
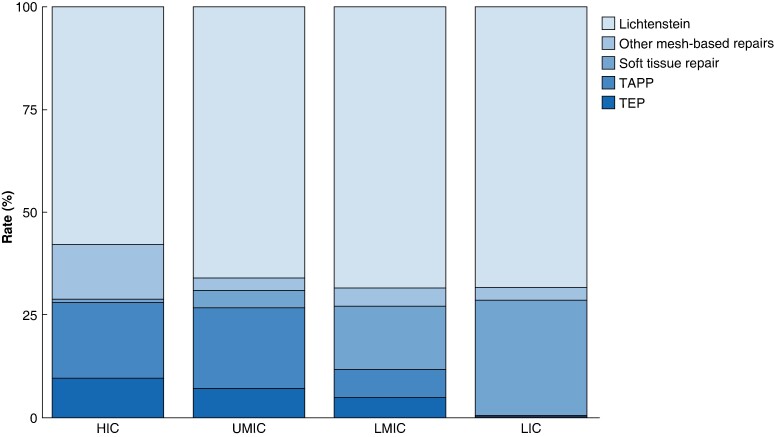
Variation in surgical technique across income groups TAPP, transabdominal preperitoneal repair; TEP, totally extraperitoneal repair; HIC, high-income country; UMIC, upper-middle-income country; LMIC, lower-middle-income country; LIC, low-income country.

In all income groups, patients were more commonly operated on by a senior surgeon (71.0%, 10 487 of 14 768) (*Table 2*). Most had previous experience of greater than 200 inguinal hernia repairs (53.1%, 7833 of 14 768). There was heterogeneity in the surgical technique chosen to repair inguinal hernia, as shown in *Fig. 2*. The Lichtenstein technique was used for greater than half of patients in all groups (61.7%, 9117 of 14 768). Of the techniques using a minimally invasive approach, transabdominal preperitoneal repair was twice as commonly used as totally extraperitoneal repair (7.8%, 1155 of 14 768). The use of both decreased from high- to low-income countries. The use of soft tissue repair was more commonly used in low-income countries (28.0%, 178 of 635). More details regarding surgical technique variation are available in *Table S3*.

**Table 2:** Surgical variation and outcomes across income groups

	HIC (*n* = 8916)	UMIC (*n* = 2575)	LMIC (*n* = 2642)	LIC (*n* = 635)	Total (*n* = 14 768)
**Primary operator**					
Senior surgeon	6568 (73.7)	1868 (72.5)	1653 (62.6)	398 (62.7)	10 487 (71.0)
Trainee surgeon	2338 (26.2)	665 (25.8)	951 (36.0)	232 (36.5)	4186 (28.3)
Non-surgeon	10 (0.1)	42 (1.6)	38 (1.4)	5 (0.8)	95 (0.6)
Missing, *n*	0	0	0	0	0
**Previous experience (number of repaired hernias)**					
0–50	1472 (16.5)	523 (20.3)	534 (20.2)	223 (35.1)	2752 (18.7)
51–200	2400 (27.0)	591 (23.0)	1004 (38.0)	172 (27.1)	4167 (28.2)
>200	5030 (56.5)	1461 (56.7)	1102 (41.7)	240 (37.8)	7833 (53.1)
Missing, *n*	14	0	2	0	16
**Surgical approach**					
Open	6303 (70.7)	1865 (72.4)	2311 (87.5)	628 (98.9)	11 107 (75.2)
Laparoendoscopic	2397 (26.9)	699 (27.1)	316 (12.0)	6 (0.9)	3418 (23.1)
Robotic	144 (1.6)	4 (0.2)	4 (0.2)	0 (0.0)	152 (1.0)
Converted	72 (0.8)	7 (0.3)	11 (0.4)	1 (0.2)	91 (0.6)
Missing, *n*	0	0	0	0	0
**Use of mesh**					
Yes	8842 (99.2)	2465 (95.7)	2231 (84.4)	457 (72.0)	13 995 (94.8)
No	74 (0.8)	110 (4.3)	411 (15.6)	178 (28.0)	773 (5.2)
Missing, *n*	0	0	0	0	0
Type of mesh used*					
Permanent synthetic	7964 (90.1)	2184 (88.6)	2055 (92.2)	417 (91.2)	12 620 (90.2)
Absorbable synthetic	608 (6.9)	193 (7.8)	155 (7.0)	39 (8.5)	995 (7.1)
Biological	7 (0.1)	9 (0.4)	1 (0.0)	1 (0.2)	18 (0.1)
Composite	261 (3.0)	79 (3.2)	18 (0.8)	0 (0.0)	358 (2.6)
Missing, *n*	2	0	2	0	4
Suture used to fix the mesh*					
Absorbable	2112 (23.9)	542 (22.0)	373 (16.7)	49 (10.7)	3076 (22.0)
Non-absorbable	3870 (43.8)	1449 (58.8)	1617 (72.5)	407 (89.1)	7343 (52.5)
Glue	641 (7.3)	6 (0.2)	3 (0.1)	0 (0.0)	650 (4.6)
Tackers	872 (9.9)	305 (12.4)	186 (8.3)	0 (0.0)	1363 (9.7)
Not fixed	1345 (15.2)	163 (6.6)	50 (2.2)	1 (0.2)	1559 (11.1)
Missing, *n*	2	0	2	0	4

Values are *n* (%) unless otherwise indicated. *Only evaluated in patients undergoing inguinal hernia repair with mesh (*n*=13 995). HIC, high-income country; UMIC, upper-middle-income country; LMIC, lower-middle-income country; LIC, low-income country.

**Fig. 3:**
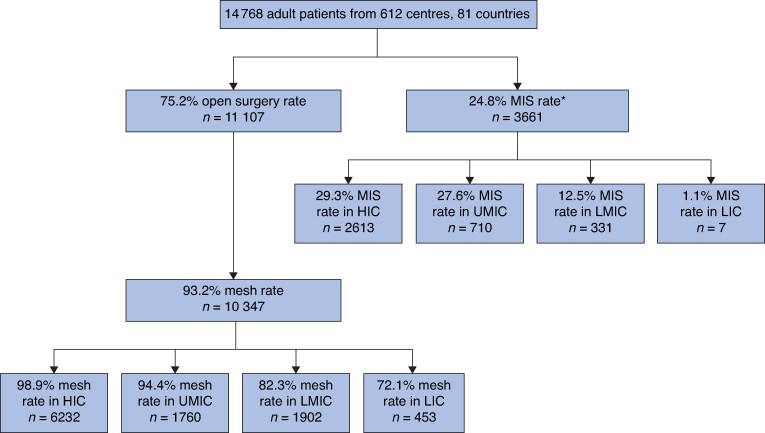
Use of technologies across income groups *Of the patients undergoing minimally invasive surgery, 99.6% (3648 of 3661) had mesh repair. There were no missing data for surgical approach and mesh use. MIS, minimally invasive surgery; HIC, high-income country; UMIC, upper-middle-income country; LMIC, lower-middle-income country; LIC, low-income country.

Overall, 94.8% of the patients had mesh placed to repair the inguinal hernia (13 995 of 14 768) (*Table 2*). When the approach was open, mesh was used in 93.2% of the repairs (13 995 of 14 768) (*Fig. 3*). There was a reduction in mesh use from high-income countries (98.9%) to low-income countries (72.1%). In the group of patients where mesh was used, the most frequent type of mesh was permanent synthetic (90.2%, 12 620 of 13 995) and the most common suture used to fix the mesh was non-absorbable (52.5%, 7343 of 13 995).

Less than a quarter of patients underwent minimally invasive surgery (24.8%, 3661 of 14 768) (*Fig. 3*). Laparoendoscopic surgery accounted for most of the minimally invasive surgery across all income groups, as shown in *Table 2*. In general, there was a higher proportion of patients operated on by senior surgeons in laparoendoscopic surgery (87.6%, 3074 of 3508) with a higher previous experience, as shown in *Table S4*.

### Predictors of access to technologies

**Fig. 4:**
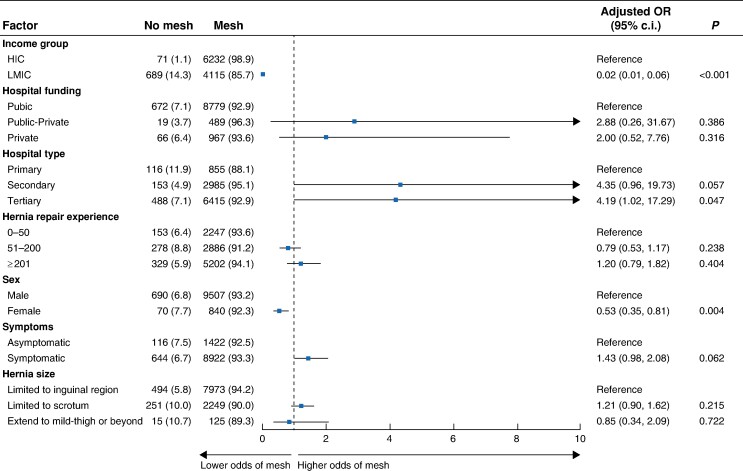
Predictors of mesh use in open surgery Only patients undergoing open surgery were included in the model (*n*=11 107). HIC, high-income country; LMIC, low–middle income countries, includinng upper-middle-, lower-middle-, and low-income countries.

Patients undergoing open surgery where mesh was not used were younger (median age of 49.0 years *versus* 61.0 years), had fewer co-morbidities, and had larger hernias, as shown in *Table S5*. However, in the adjusted analysis, being operated on in a low–middle-income country was associated with lower mesh use (adjusted OR 0.02 (95% c.i. 0.01 to 0.06); *P* < 0.001) (*Fig. 4*). Being female was the only other factor that was associated with lower use of mesh in open surgery (adjusted OR 0.53 (95% c.i. 0.35 to 0.81); *P* = 0.004). Of the other factors tested, none had a significant association with use of mesh.

**Fig. 5:**
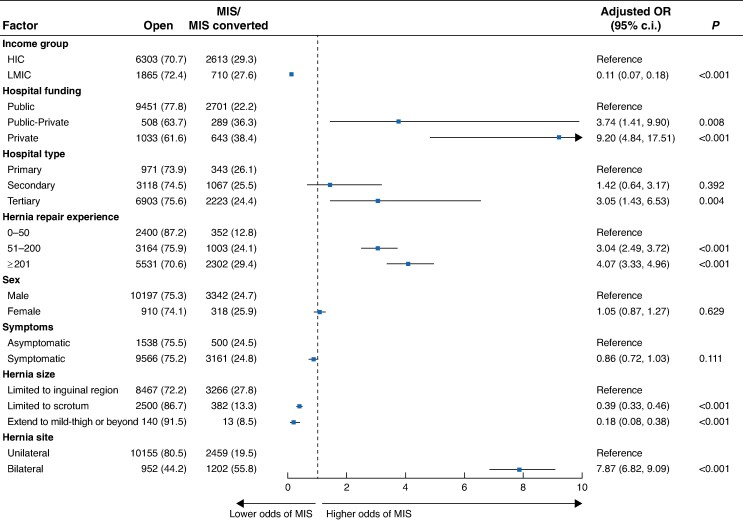
Predictors of use of minimally invasive surgery MIS, minimally invasive surgery; HIC, high-income country; LMIC, low–middle income countries, including upper-middle-, lower-middle-, and low-income countries.

Patients undergoing hernia repair in low–middle-income countries was associated with lower odds of minimally invasive surgery use (adjusted OR 0.11 (95% c.i. 0.07 to 0.18); *P* < 0.001) (*Fig. 5*). Having an inguinal hernia limited to the scrotum or that extended to the mid-thigh or beyond was associated with lower odds of minimally invasive surgery. Of the hospital factors tested, being operated on in a private hospital (adjusted OR 9.20 (95% c.i. 4.84 to 17.51); *P* < 0.001) and in a tertiary-level hospital (adjusted OR 3.05 (95% c.i. 1.43 to 6.53); *P* = 0.004) were both associated with higher odds of use of minimally invasive surgery. Of the patient factors tested, having a bilateral hernia repair was associated with higher use of minimally invasive surgery (adjusted OR 7.87 (95% c.i. 6.82 to 9.09); *P* < 0.001).

### Postoperative complications

The postoperative complication rate was 13.7% and most of the postoperative complications were minor, i.e. Clavien–Dindo grade I–II (93.5%, 1808 of 1933) (*Table S6*). Patients undergoing minimally invasive surgery had a postoperative complication rate of 11.3% (415 of 3661) (*Table 3*). Patients where mesh was used had a postoperative complication rate of 13.1% (1828 of 13 995). Overall, at 30 days, the surgical-site infection rate was 3.0% (445 of 14 768) and the reoperation rate was 0.5% (75 of 14 768).

**Table 3:** Variation in complications, surgical-site infection, and reoperation at 30 days with regard to surgical approach and use of mesh

	Complications at 30 days	Surgical-site infection at 30 days	Reoperation at 30 days	Total
Surgical approach*				
Open surgery	1518 (13.7)	397 (3.6)	45 (0.4)	11 107
Minimally invasive surgery	415 (11.3)	48 (1.3)	30 (0.8)	3661
Missing, *n*	0	0	0	0
**Use of mesh**				
Yes	1828 (13.1)	408 (2.9)	67 (0.5)	13 995
No	105 (13.6)	37 (4.8)	8 (1.0)	773
Missing, *n*	0	0	0	0

Values are *n* (%) unless otherwise indicated. *Surgical approach classified as intention-to-treat. Minimally invasive surgery includes laparoendoscopic, robotic, and converted surgeries.

## Discussion

The prinicipal finding of this study is the lack of access to mesh observed in low- and middle-income countries. This was shown in open repair, as well as in all hernia repairs included, regardless of the approach. With regard to open surgery, having the hernia repair in low–middle-income countries was the most important factor found to be associated with lower use of mesh. Lower use of mesh not only has a direct impact on patients, who will have a higher risk of hernia recurrence^4^, but also demonstrates that access to mesh technology is limited.

This study also shows low use of minimally invasive surgery across all income groups. Overall, less than a quarter of patients were operated on using minimally invasive surgery and, when used, laparoendoscopic-based techniques were preferred. This was even lower in low–middle-income countries. However, having the repair in a private or tertiary-level hospital and having a bilateral hernia were all associated with higher use of minimally invasive surgery.

The data from this study are relevant for developing plans to expand the use of mesh and minimally invasive surgery.

There is a global need to increase access to and training programmes for mesh inguinal hernia repair in low–middle-income countries^19^. Mesh is a simple device that has been recommended by international guidelines for the treatment of inguinal hernias^2^ and is recognized as standard practice by several hernia societies globally^20–22^. Using mesh reduces recurrence rates, avoids further operations, and has been shown to be cost-effective^4,23^. Therefore, upscaling mesh use in inguinal hernia patients should be a first priority in providing access to more advanced technologies^20^. Supply chains, training surgical teams, and reducing costs of mesh for patients are all factors that have been identified previously as barriers to access to mesh technology and that could be targeted^1^.

Expansion of minimally invasive surgery in well-resourced settings will require expansion of dedicated training programmes and a focus on patients who will benefit most. In settings where expertise is available, minimally invasive surgery is the recommended approach for inguinal hernia repair, according to international guidelines^2^. However, the low use of minimally invasive surgery in this study, even in high-income countries, leads to concerns regarding inherent training challenges and slower learning curves^24,25^. There is also the potential lack of agreement in the wider general surgical community regarding the clinical benefit outside of selected groups of patients, such as patients with bilateral hernias, patients with recurrent hernias, and female patients^26^.

There are limitations associated with this study. Representation by low–middle-income countries in this setting was low. Countries with better access to technologies might not have been captured. Also, in the high-income group, there was a lack of representation by centres from countries that are reported to have higher rates of minimally invasive surgery (for example Sweden and Denmark)^27^ and this might have resulted in a low estimation of minimally invasive surgery use amongst this group. Complication data were only collected at 30 days after surgery, which limits the evaluation of recurrence, which is an important longer-term outcome of hernia repair. However, there is already good evidence showing higher recurrence rates when mesh is not placed, even in low-income settings^5,11^.

Future research is still needed. Full understanding of the payment mechanisms available in different countries will help to identify economic barriers to access to mesh technology. National evaluation of payment options and avoidance of out-of-pocket expenses might improve access to mesh and other technologies, by protecting patients from catastrophic expenditure.

This study provides relevant information to policymakers on potential targets to improve access to simple technologies, such as mesh. To achieve medium- to long-term improvement, it will be essential to train surgical teams on site. Partnerships between high-income countries and low–middle-income countries could be useful to co-develop a recognized global training package. Involving hernia societies and national surgical colleges based in low–middle-income countries could expand the training programme, while monitoring its quality and safety. Expanding mesh use could be a first step, before expanding the use of more advanced technologies, for which training is more demanding, supply chains are more complex, and the costs are higher.

## Supplementary Material

znae164_Supplementary_Data

## Data Availability

Anonymized data are available upon request from the writing group and successful completion of a data sharing agreement through an Application Programming Interface linked to the REDCap data server hosted at the University of Birmingham, Birmingham, UK.

## Collaborators

**NIHR Global Research Health Unit on Global Surgery:**

M Picciochi, AO Ademuyiwa, A Adisa, AE Agbeko, JA Calvache, D Chaudhry, R Crawford, AC Dawson, M Elhadi, A Ghaffar, D Ghosh, J Glasbey, P Haque, E Harrison, A Isik, I Jakaityte, SK Kamarajah, O Kouli, I Lawani, S Lawani, V Ledda, E Li, J Martin, A Minaya Bravo, D Morton, D Nepogodiev, F Ntirenganya, O Omar, SZY Ooi, R Oppong, F Pata, A Ramos-De la Medina, M Sampaio-Alves, JFF Simoes, M Steinruecke, S Tabiri, A Bhangu.

A A Elhars, Y A. Omar, S Ababneh, R Abad, A Abaidalla, E Abate, H Abbadi, AM Abbas, A Abbas, A Abbas, A Abbas, M Abbas, S Abbas, W Abbas, J Abbasy, B Abboud, M Abd Al-Fattah, R Abd Alkareem, R Abd Elkareem, A Abd Elsattar, NS Abd Ghani, D Abdalaziz, A Abdalhadi, A Abdalla, I Abdalla, S Abdeewi, H Abdelazim, E Abdelbaset, M Abdelfattah, M Abdelhadi Suliman Adam, M Abdelhafez, AR Abdelhalim, A Abdelhamid, M Abdelkabir, M Abdelkarim, M Abdelmaboud, M Abdelmaksoud, A Abdelmalik, S Abdelmohsen, ASM Abdelrahman, S Abdelrasoul Elnour Ismail, M Abdelreheem, MEEA Abdelsalam, A Abdelshafi, B Abdennour, A Abdllah, H Abdoun, A Abdraba, E Abdu, M Abdu, My Abdualqader, AA Abdul Rahim, A Abdul Rahman, S Abdul Rahman, I Abdullah, M Abdullah, U Abdullah, LB Abdullahi, M Abdullahi, M Abdullahi, S Abdullateef, TMA Abdulmola, A Abdulnabi, NT Abdulraheem, D Abdulrahman, K Abdulrahman, T Abdulrahman, M Abdulsalam, L Abdur-Rahman, JT Abebrese, M Abed, S Abed, R Abela, T Abelman, S Abeykoon, D Abeysirigunawardana, R Abhinaya, A Abidali, A Abiodun, H Abiyere, M Abo Abdo, A Abo Al Shamat, ZA Abo Alarous, I Abo Elhagag, K Abo Zaal, C Aboah, AGE Aboelnasr, M Abosedra, S Aboseif, AAA Aboshosha, AGMM Abouelnagah, G Abouelnagah, O Abouhiekal, A Aboumedian, H Abozied, S Abrayik, A Abreu, A Abreu Da Silva, N Absy, M Abu Al Amrain, MK Abu Albahrain, M Abu Daoud, M Abu Jayyab, S Abu Khousa, H Abu Obead, R Abu Salah, S Abu Salem, E Abu Siam, A Abu Tair, H Abu-Arish, M Abubakar, A Abubakar Abdulkarim, M Abudabbous, A Abuhammad, A Abuhantash, A AbuNemer, D Abunemer, H Abuobaida, EE Abuobaida Banaga Hag El Tayeb, I Aburumman, H Abusnina, A Abuthaher, J Abutu John, M Abuwarda, S Abuzahra, M Acar, JD Acevedo Parrales, R Acharya, S Achugatla, L Ackam, E Ada, L Adagrah Aniakwo, AA Adam, E Adam, M Adamina, P Adebayo, E Adebunmi, A Adekoya, A Adel, I Adel, R Adel Diab, E Adel Hamdoun Aziz, E Adel Mahmod Sultan, A Ademuyiwa, A Aderounmu, MA Adetoyi, A Adewumiya, A Adeyeye, I Adham, H Adi, M Adinku, E Adinolfi, D Adjei, H Adjei, J Adjei, Y Adofo-Asamoah, GA Adoro, C Adumah, G Aduroja, W Afedo, E Afeikhena, N Affram, F Afonso, H Afzal, S Agana, M Agapov, C Agarwal, E Agastra, M Agbadebo, A Agbaje, V Agbakwuru, K Agbedinu, FM Agbemafoh, DYD Agbley, N Agboadoh, FDA Agbodo, C Agbonrofo, P Agbonrofo, E Agbowada, B Ağca, O Agcaoglu, M Aggarwal, M Aggelidou, O Agha, A Aghayeva, S Agordjor, MK Agrawal, H Aguado López, SV Agudelo Mendoza, C Aguero, N Agugua-Obianyo, JA Aguilar, F Aguilar Del Castillo, M Aguilera-Arevalo, M Agunloye, MM Agyapong, R Agyei Boakye, T Agyen, JL Ahale, A Ahmad, A Ahmad, F Ahmad, H Ahmad, O Ahmad, AAH Ahmad Zaidi, A Ahmayda, AS Ahmed, A Ahmed, H Ahmed, EA Ahmed, GMG Ahmed, IMG Ahmed, I Ahmed, I Ahmed, M Ahmed, M Ahmed, M Ahmed, R Ahmed, S Ahmed, S Ahmed, K Ahmed Ibrahim, M Ahmoud, S Ahuja, F Aigner, M Aikins, R Ait Ben Addi, A Ajayi, SA Aji, O Ajiboye, EB Akakpo, R Akankoatuesi Apatewen, G Akcakoca, A Akeem Aderogba, C Akgün, T Akharaekpanya, F Akhtar, A Akiba, J Akiely, N Akinbami, A Akinkuolie, A Akinmade, F Akinwande, M Akkawe, C Akli-Nartey, A Akmercan, R Akpaka, U Akram, MK Aktas, K Akter, T Akter, YE Aktimur, S Akuffo, F Al Abbood, O Al Alyani, N Al Amri, H Al Atassi, S Al Athath, AM Al Balushi, Z Al Balushi, T Al Barhi, J Al Daradkah, O Al Hamdani, A Al Hammoud, K Al Hinai, M Al Hinai, A Al Kaddah, J Al Karmi, H Al Miskry, S Al Momani, A Al Mukhtar, H Al Qadhi, N Al Rabadi, M Al Sayed, O Al Shaqran, S Al Sharie, R Al Shehhi, H Al Sohabi, B Al Soudi, M Al Zebda, H Al-Aamri, H Al-Abdallat, M Al-Attraqchi, Z Al-Azher El-Hamel, A Al-Bahla, Z Al-Balushi, L Al-Boukhari, H Al-Derume, M Al-Dhaheri, S Al-Doghme, H Al-Fahel, O Al-Fahel, A Al-Fandi, M Al-Fraijat, ABA Al-Hajjaj, N Al-Hroub, MA Al-Juaifari, Y Al-Junaidi, W Al-Khyatt, A Al-Mallah, M Al-Masri, A Al-Mouahhed, M Al-Qannas, B Al-Sarireh, K Al-Shami, M Al-Shehari, Z Al-Sheikh Ali, S Al-Tahayneh, MA Al-Yusuf, H Al-Zoubi, A Al-Zubeidy, O Alaba, E Alabed, A Aladl, A Alagha, F Alahmad, A Alahmad Alismael, A Alailesh, J Alaji, F Alakaloko, Y Alalawi, A Alamin, S Alananzeh, M Alansary, A Alaqtash, N Alasbahi, S Alashhab, F Alassani, R Alatawi, Y Alawneh, A Alayed, B Alazabi, M Alazabi, A Albager, N Albahloul, B Albaihani, A Albalawi, H Albalawi, J Albalushi, MEH Albanna, A Albatanony, A Alberico, MJI Albert, D Alberti, S Alberti, M Albertsmeier, A Albhaisi, TA Albushary, H Aldare, Y Aldebasi, AN Aldirani, T Aldirawi, A Aldurssi, A Alecci, G Alemayehu, P Alexander, D Alexandrou, M Alfaid, H Alfakeer, F Alfarsi, A Alfarwan, B Algettawi, G Alhadwah, A Alhaj, T Alhaj Hasan, A Alhaj Zain, A Alhamadi, A Alhammali, Y Alhammoud, M Alharthi, A Alhebshi, M Alhimyar, W Alhroub, H Alhusaini, T Alhusban, A Ali, A Ali, A Ali, A Ali, B Ali, B Ali, E Ali, F Ali, F Ali, I Ali, I Ali, MA Ali, M Ali, M Ali, N Ali, N Ali, M Ali-Hassanzadeh, E Alić, D Alifoe, s Alijla, R Alinde, T Alinger, S Aliozor, F Aliskander, MS Aliyu, G Alıcı, I Aljada, A Aljahdali, Z Aljalabi , A Aljamoudi, M Aljarawn, S Alkadi, A Alkaseek, Z Alkhaier, E Alkhalifa, J Alkharish, A Alkharusi, H Alkhatib, R Alkhatib, Z Alkhuzaie, AM Allam, S Allam, L Allan, Y Allaoua, B Allbakosh, JC Allen, C Allmer, R Alloni, A Alloush, WJN Almadhoun, RKZ Almahadin, T Almahdi, M Almahjoub, M Almaletti, M Almaqrahi, MM Almihashhish, G Almogy, O Almomani, AAY Almugaddami, A Almutairi, Z Alnajjar, N Alnamari, F Alnazawi, M Alneasan, S Alneihuom, A Alniemi, I Alnimer, F Alnimri, M Alnuwayli, L Alokshi, V Alonso, S Alonso Marcos, T Aloqaili, M Aloulou, F Alowjaly, C Alphonse, M Alqadasi, B Alqahtani, R Alqahtani, M Alqedrh, A Alqerem, S Alqurashi, A Alrababah, A Alragheai, M Alrantisi, B Alrayes, G Alrayyes, A Alrifaee, L Alriyami, DS Alrokh, A Alroobi, O Alruwaili, W Alsado, S Alsaeiti, B Alsaid, AA Alsalahat, M Alsalawi, M Alshaar, S Alshab, S Alshagrawi, A Alshahrani, B Alshaikh, M Alshami, M Alshamikh, M Alshanwani, B Alsharari, M Alsharayri, AH ALSharqi, T Alshawabkeh, M Alshehri, A Alsheikh, G Alsheikh, A Alshiteewi, F Alshreef, A Alshukre, S Alsibai, S Alsmadi, M Alsori, I Alsoubie, A Alsultan, S Alsuwiyah, H Altabbaa, S Altaf, M Altajouri, AA Altawaiha, H Altawati, R Altayargh, M Althomali, Y Altinel, O Altobaishat, S Altoume, H Altounsi, A Alusef, R Alvarado Hurtado, A Alvarez Cuiñas, JM Alvarez Hernandez, DG Alves, N Alwaer, A Alwali, A Alwali, S Alwardi, NK Aly, A Alzadjali, A Alzahrani, N Alzerwi, B Alzoubi, A Alzu'Bi, R Alzu'Bi, R Alzughayyar, H Alzuhd, M Amadu, C Amah, S Amahmid, A Amaigl, D Amangaliyev, I Amankwaa, G Ambriz González, MO Ameen, A Amendola, E Amer, E Ametefe, A Amin, S Amin Omar Alsiddig, H Amin-Tai, B Aminu, FN Amir, H Amir, MF Amisano, AS Ammar, F Amoako, M Amoako-Boateng, L Amosu, H Amoudi, S Amoza Pais, J Ampadu, K Anaam, A Anadani, A Anand, A Anand, H Anand, M Anandan, P Anandan, A Anastasi, M Anati, S Anaya Sanchez, D Anbu, AD Andani, E Andolfi, N Andrade, S Andrade, P Andrejevic, R Andreou, L Andreski, J Andreuccetti, E Anestiadou, A Ang, M Angelini, M Angelucci, S Anim, R Anjum T Siddeek, J Annan, P Anoldo, C Ansah Larbi, G Ansong, A Antinori, P Antonakis, E Antoniou, AAA Anuar, L Anyanwu, JN Anyorigiya, F Anzinger, H Aouagbe Behanzin, H Aouagbe Behanzin , D Aparicio-López, E Apostolopoulos, AY Appiah-Kubi, A Appukuttan, G Aprea, S Arafa, M Arafat, M Araydah, H Arbogast, AB Aregawi, A Arekhandia, II Aremu, R Arena, N Arkadopoulos, EA Arkoh, R Armah, VE Armenta Tapia, AY Arnaout, I Arnaout, M Arnaout, AP Arnaud, O Arsalan, PC Arteaga Asensio, SJ Arthur, P Artigot, N Aruldas, P Arunachalam, A Arustamyan, B Arya, PK Arya, H Asaad, M Asaad, Z Asaad, Y Asano, Y Asar, M Asarbakhsh, A Asare Twumasi, F Ascari, KV Ascencio Diaz, A Asekun, J Aseme, L Asensio Gomez, AA Asharaf, S Ashini, A Ashiru, D Ashitey, T Ashkar, ME Ashong, Y Ashour, F Ashraf, F Ashraf, S Ashry, M Asimakidou, S Asiri, R Askari, H Askarpour, AE Askin, A Asla, A Aslan, M Assalhi, C Assis, P Assouto, JR Asturias Luna, MI Ateş, A Athanasiou, E Athanasopoulou, K Athanassiou, K Atherton, D Atouguia, R Attaf, O Attawodi, D Attia, A Attili, SAA Atupra, G Augustin, M Aulicino, YL Aung, A Autiak Ayii Chol, J Avakoudjo, MA Avci, K Avgerinos, A Awad, AK Awad, S Awad, T Awad, G Awais, A Awidan, A Awwad, NA Awwad, E Ay, K Ayad, E Ayala, O Ayandipo, F Ayasra, E Aybar, A Ayeni, H Ayesh, A Ayoade, BA Ayoade, A Ayodele, K Ayyoub, A Azam, B Azhar, Z Azhar, R Azimov, M Aziz, CE Azmat, A Azzinnaro, MMR Azzuz, SAM Ba-Shammakh, CA Baars, I Babajimi-Joseph, O Babatunde, S Babu, B Baca, EA Baccalini, I Bacic, FG Bader, M Badkur, Y Badr, R Badra, D Badran, B Badwan, Z Bady, IW Bahikoro, M Bahnacy, O Bahsas-Zaky, A Baiden Amissah, E Baili, L Bains, M Bajwa, A Bakalis, IN Bakaweri, E Baker, A Bakri, V Bakshi, A Baksi, B Balagobi, D Balalis, B Balaravi Pillai, Z Balciscueta, A Balconi, R Baldan, E Baldini, E Balik, O Balogun, YL Balogun, A Balta, C Banal, K Bananis, F Banchini, S Bandyopadhyay, N Banerjee, A Bang, A Bangar, S Bani Amer, M Bani Hani, C Banka, E Bankart, D Baños Méndez, E Bara, A Barakat, M Barakat, MM Barakat, M Barbarawi, A Barberis, FJ Barbosa Camacho, S Barbosa Castelo Branco, MD Barcelona, A Barcin, D Bardsley, J Barklimore, E Barkolias, A Barlas, L Barnard, L Barni, R Barone, G Baronio, R Barradas, AG Barranquero, NG Barrera Lopez, MT Barrio Renteria, J Barrutia Leonardo, A Bartalini Cinughi De Pazzi, A Basalim, H Basharat, M Bashir, MA Bashir, G Bashour, E Basile, R Basile, R Bassam, A Bassma, SMM Basso, S Basu, S Basu, S Bataineh, R Batir, S Batista, M Battili, M Bautista, A Bavaharan, A Baydoun, NS Bayleyegn, H Baysal, S Beavis, G Bechara, O Bedak, A Bediako Bowan, A Bedzhanyan, E Bedzhanyan, E Begunić, N Beharry, M Bei, M Bejarano Serrano, G Bekakos, K Bekiaridou, S Bektas, A Belaid, A Belarmino, D Belekar, M Beletachew, A Belkhair, A Belli, MI Bellini, G Bellio, F Bello, M Bello, O Bellou, P Belotti, R Belouz, J Beltrán De Heredia, N Ben Hasan, R Ben Hmida, M Benamrouche, ZR Benamrouche, B Bengaly, F Benghalbon, M Benghazi, A Benmansour, S Bennett, S Bensalem, K Bensmain, MA Bequis, B Bereket Araya, G Berger, M Berlet, R Bernardino, M Bernardo, P Bernardo, M Berrakkouch, F Berrevoet , M Berselli, CL Bertoglio, S Bertone, M Bertrand, R Bethune, E Betz, L Beukes, M Bevanda, C Bezede, A Bhadani, A Bhargava, GA Bhat, M Bhat, N Bhat, R Bhatia, A Bhatt, A Bhatti, HW Bhatti, K Bhatti, W Bhatti, K Bhutia, A Biancafarina, M Biancu, A Bibi, NS Bibila, E Bičakčić, M Bickford, M Biebl, H Bileid Bakeer, M Bilfaqirah, IA Bilgin, C Bilir, V Bill, M Billis, LN Bin Aizan, MM Bin Khalid, B Binda, M Binda, A Binder, J Binder, M Binks, NA Binti Yusri, F Biolchini, G Birikorang, SL Birolo, G Birqeeq, E Bisogno, S Bitsianis, M Blanco, JL Blas Laina, N Blencowe, NS Blencowe, B Boakye, K Boakye-Acheampong, PA Boateng, PK Boateng, C Bode, M Bogdan, B Bogdan-Gabriel, M Bohlala, A Bojazyah, I Bokos, C Bokossa, LI Bolaños, J Bolanowski, H Bolukbasi, G Bond-Smith, A Bonelli, L Bonello, LD Bonomo, K Booyse, M Boras, G Borda-Luque, A Borello, E Borges, I Borges Da Costa, V Borgogni, N Börner, G Boroni, S Borrego Canovaca, S Botaitis, A Botha, C Bôto, K Bouchagier, IE Boudis, MN Bouhafs, A Bouhuwaish, O Boujidi, K Bouliaris, IE Boumakhlouf, N Boumas, C Boven, M Bowles, S Bowman, S Boyes, A Boynes, K Bozada-Gutiérrez, E Bozdağ, AÇ Bozkurt, E Bozkurt, AE Boztaş Demir, L Bracciano, G Brachini, R Bradley, H Bradly, CJ Bradshaw, S Bradulskis, H Braham, K Bramis, T Branco, C Brandi, R Branquinho, A Brar, C Brasset, L Bravo, LD Bray, Z Brekalo, SG Brenu, S Brincat, M Brito, M Brizzolari, V Brocco, A Broglia, E Brolese, L Bromley, A Brosin, GD Brown, F Brucchi, A Brun-Peressut, M Brunner, J Bryan, F Brzeszczyński, SS Budhwani, V Budyakova, N Bugdayci, S Bugren, FM Bujalance Cabrera, GC Bulbuloglu, J Bundred, S Burger, M Busch, MF Butt, A Butyrskii, F Buyuker, KJ Bwala, G Cabezudo, LR Cabezudo, A Cabral, NN Cabrel, I Cabrera, PA Cabrera Rivera, M Caccetta, R Cachia, EP Cagigal Ortega, S Cai, F Caiquo, I Caitens, I Çakır, A Çakmak, B Cakpo, B Calcaño, M Calderon, B Calik, V Çalik, G Calini, PG Calò, A Caltagirone, F Camacho, O Camilleri, F Cammelli, M Cammelli, M Campanelli, A Campbell, R Campbell, F Campus, M Camuera, M Canals Sin, O Čančar, MD Cancelas, G Canonico, GL Canu, B Capdevila Vilaró, L Capello, H Capote, F Cappellacci, M Cappiello, M Capuano, LJ Caram, MM Caramantin Obando, L Carbone, M Carbonell Pradas, S Cardelli, G Cárdenas Rivera, L Cardinali, DC Cardona Gomez, C Cardoso, A Cardoso Almeida, G Carganico, JM Carlos, P Carmignani, G Carollo, O Carpineto Samorani, Y Carpio Colmenares, S Carrabetta, JM Carranza Rosales, AL Carreira-Marques, C Carrillo Sarango, J Carrizo, A Carta, D Caruso, B Carvalho, M Carvalho, L Casalduero, F Casas J, M Casati, JR Casella Mariolo, L Casingena, R Casma Bustamante, J Cassiano Neves, A Castaldi, F Casti, F Castro, L Castro, RJ Castro Lara, M Castrovillari, L Catozzi, M Catterall, MDP Cebollero, C Cecconi, C Celano, N Celestine, B Celik, A Cembellin, DC Centonze, M Ceolin, M Ceresoli, A Cerovac, I Cervera, AF Cetişli, M Chaccour, P Chadha, F Chahrour, S Chaifouroosh Mamagany, D Chakarov, Y Chamara, G Chamoso Mialdea, A Chamzin, E Chan, J Chan, M Chandrasegaram, G Chang, L Chang, L Chantada, G Charalambous, L Chardalias, S Charitonos, S Chase, D Chatterjee, P Chatzikomnitsa, C Chatzinikolaou, I Chatzis, A Chaudhary, R Chaudhary, S Chaudhary, A Chaudhry, NK Chaudhry, H Chauhan, M Chauhan, C Chavarría Noya, N Chavarrias, D Chavez Fernandez, M Che Yaacob, HK Cheema, JM Chejfec-Ciociano, AJ Chekfa, JJQ Chen, N Chen, LP Cheng, C Cheong, ACK Cheung, M Chevallay, J Chew, A Chhabra, SYD Chia, P Chiacchio, C Chiam, A Chiappini, N Chidumije, A Chied, LR Chieng, SP Chigblo, S Chilaka, G Chillarge, BG Chiman, P Chimezie Andrew, D Chimkaomasiri, E Chin, Z Chin, MH Chishti, M Chisthi, A Chitul, B Chkir, P Chloropoulou, I Cholah, K Cholah, J Chóliz, L Chong, S Chopra, T Chouari, AM Choudhary, A Choudhrie, A Choudhry, C Chouliaras, SA Chowdhury, S Chowdhury, NA Christian, N Christian, O Christianah, G Christodoulidis, P Christodoulou, S Christodoulou, M Christou, K Chu, E Cianci, P Cianci, D Cianflocca, F Ciccarone, E Ciccioli, PM Cicerchia, L Cidade Costa, AB Ciftci, V Cijan, N Cillara, C Cini, E Ciofic, B Cirillo, B Cismasiu, B Citgez, C Ciubotaru, D Clarke, I Clementi, L Cobellis, N Cobeño Tamayo, G Cocorullo, L Codina Corrons, O Cohen-Arazi, I Colaço, E Colak, R Colbran, K Collins, E Colton, A Comandatore, A Comella, S Condron, L Conti, I Contis, I Conversano, F Coratti, M Corcos, B Cordeiro, D Córdova García, C Cornwell, M Coronas Soucheiron, ZM Correa López, P Correia, F Corronca, S Corso, F Corvatta, C Costa, S Costa, A Costas-Chavarri, C Coutinho, G Cox, O Coyoy-Gaitán, I Ćoza, M Cricrì, D Cristian, A Crowe, M Cruz, OM Cuenca Torres, M Cuesta Argos, M Çuhadar, J Cui, V Cuk, B Cuneo, A Curado Soriano, Sathiafrey D A, F d'Acapito, F D'Agostino, A D'Amore, MR D'Anna, A D'Ignazio, B D'Souza, C Da Lio, H Daaboul, D Daary, E Dabbagh, H Dąbrowski, N Dadoush, N Dafnios, M Daher, A Dahiru, E Dainius, G Dajti, I Dajti, E Daketsey, H Dalati, J Daleku, E Daleva, C Dally, C Damaskos, A Damigella, A Damola-Okesiji, R Damseh, M Daniyan, RA Dar, R Daradkeh, A Darkwa Boateng, A Das, K Das, M Dason, U Daspal, V Dassah, I Datji, A Dauksa, Z Dauksa, E Daukšaitė, M Dauphin, S Davakis, P David Santos, B Davies, A Davis, S Davis, A Davolio, A Davor, S Dawo, A Dawoud, B Dawud, B De Andrés-Asenjo, B De Andrés-Asenjo, G De Angeli, G De Carlo, J De Deken, VU De Donato, JM De Francisco Rios, I De Haro Jorge, Á De Jesús Gil, S De La Cruz Ahufinger, G De La Peña González, E De Leo, C De Martino, P De Nardi, GD De Palma, M De Prizio, N De Santis, WDD De Silva, Y De Silva, G De Wee, B De Zolt Ponte, Y Dean, S Debrah, J Deeb, L Degrate, J Dei-Asamoa, G Del Corpo, P Del Val Ruiz, H Delacave, G Delgado Hernandez, S Delibegovic, D Dellaportas, A Dembele, KS Dembele, A Demessie, Z Demetrashvili, A Demetriou, F Demiral, C Demiri, I Demiris, MT Demirpolat, JA Demma, C Dempers, B Demurtas, M Denini, A Denz, M Derattani, M Dery, A Deserra, S Deshpande, E Desiato, M Despotidis, T Devabalan Koil, A Develioğlu, H Devesa, L Devi, G Devidze, B Devkaran, P Devlin, E Dexter, KE Dey, P Dhamija, D Dhawan, J Dhillon, J Dhima, J Dhiman, ASP Dhinakar, S Dhole, G Di Franco, E Di Marco, F Di Marco, D Di Pietrantonio, S Di Saverio, M Diab, J Dias-Ferreira, F Diaz, R Diaz, R Díaz Pedrero, R Díaz-Ruiz, OAA Dicko, I Didier, B Dieck, U Dietz, M Diez Alonso, E Dijan, U Dilibe, N Dimitriou, N Dimitrokallis, D Dimitrov, C Dina, S Dindyal, VP Dinuzzi, N Diomede, C Distefano, Z Djama, SM Djote, JE Do, R Doamba, MÁ Dobón Rascón, A Doghaim, S Dognia, R Doherty, C Doku, MA Dokurugu, QK Dolatzay, H Dolo, T Doma Bhutia , A Domenech Plana, JC Domingues, J Dominguez, R Donchev, I Dong, J Dongmo, MA Doniquian, AE Dönmez, E Donnarumma, C Donohoe, EA Dontoh, P Dorovinis, S Dos Santos, S Dos Santos Rocha Ferreira, M Doss, F Dossou, C Doudakmanis, B Down, E Downes, E Downing, V Dragisic, R Drasovean, M Drogouti, L Dubs, JA Duffield, D Dugar, M Dugo, M Dum, B Duman, P Dummala, A Dupont, V Duque Mallén, ME Duran, II Durán Sánchez, G Duranti, Z Durna, A Duro, M Durrani, N Duru, A Dusabimana, R Duvenage, E Dvivedi, B Dweik, MF Dwikat, L Dyab, M Dyer, E Dylja, S Eam, N Eardley, B East, D Eaton, H Ebied, S Ebrahim, W Ebrahim, J Ede, M Edeeb, S Edem, M Edena, M Ediale, R Ediru, U Edith, A Edmundson, E Efremidou, A Efstathiou, E Efstathiou, E Efthymiou, A Egdeer, RA Eghonghon, S Egreara, OA Egwuonwu, A Ehab, A Eisa, L Eisner, M Eissa, H Ejaz, E Ekaladze, MT Ekanayake, G Eke, A Ekerin, A Ekpeti, N Ekwo, H Ekwuazi, OH Ekwunife, MM El Ghoul Miliotou, GN El Hunjul, AES El Kady, Y El Okazy, A El Shamarka, A El-Bastwesy, A El-Sherbiney, R Elaifia, R Elazary, A Elbalal, S Eldirdiri, O Elebute, P Elemile, M Elesseily, S Elfallah, H Elfeki, M ElFiky, S Elfurdag, MA Elgak, L Elgebaly, H Elghadban, MAM Elghriani, A Elghrieb, F Elhabishi, H Elhallaq, OA Eljizoly, D Elkafrawy, F Elkhafeefi, A Elkhalifa, AS Elkhodary, AM Elkhouly, R Ellawala, C Elliot-Wilson, S Ellouzy, M Elmesalmi, M Elmiesiry, A Elmosalamy, M Elmousili, M Elmujtaba, S Elnoamany, M Elnour, R Eloka, AM Elsayed, M Elsayed Metwally, A Elshazli Mahmoud, E Elsheikh, E Elshennawy, R Elshennawy, A Eltahir, A Eltayeb, A Elzanaty, M Elzayat, AE Elzoubi, O Emadeldeen, H Embarek, S Emile, G Emiliani, A Emran, O Emuze, J Enaholo, O Enciu, RE Enejo, V Enemuo, S Engel, D Enjuto, N Ensor, M Enßlin, L Epis, G Ercolani, M Erfan, M Ergenç, E Erginöz, M Erkaya, S Errami, C Ersavas, J Escartin, N Eshak, FJ Eshun, I Esparza Estrada, B Esposito, H Essalim, A Essamei, M Estaire Gómez, C Esteo Verdu, B Estraviz, V Etwire, M Eugene, P Evangelou, A Evans, D Evripidou, M Ewedah, K Ewool, B Eyduran, J Ezeh, U Ezidiegwu, U Ezomike, N Fabbri, S Faber, O Faboya, MV Facchino, A Fadipe, HA Fadlalmola, F Fadzlullah, M Fagbayimu, I Fagiri, J Fahed, B Fahmy, M Fahmy, D Fairbrass, A Faisal, M Faisal, R Faisal, A Fajardo, S Fakhouri, I Fakhradiyev, A Fakoya, M Faletar, A Fani, Y Farag, H Farhat, MA Farho, M Farooq, MR Farooqui, A Farrag, Z Farraj, G Farris, M Farrugia, A Farsi, OK Fasiku, O Fasoro, A Fathi, A Fatima, LM Fatucchi, O Fatudimu, A Faustino, M Favoriti, P Favoriti, K Fayed, JM Faylona, N Fazili, J Febré, L Fedele, H Fehrer, N Feijoo, S Fennelly, D Fenner, CV Feo, RL Ferlini, J Fernandes, U Fernandes, Á Fernández Camuñas, MA Fernandez Romero, MA Fernández Zurita, F Ferraina, F Ferrara, A Ferreira, M Ferreira, R Ferreira Acosta, C Ferreras García, CI Ferrero, E Ferri, G Fialho, I Fidoshev, C Figueiredo, J Figueiredo, R Figueroa, M Filardo, T Finlay, N Finocchiaro, A Finocchio, M Fiogbe, Y Fishman, M Fitri, M Flint, LA Flores Chávez, LM Flores Chávez, JA Flores Prado, CM Florián Villa, F Floris, C Floros, M Florou, H Foad, H Foda, GB Fonsi, J Fontaínhas, L Fortuna, S Fortuna Martins, I Fortune, B Fosh, LI Fountarlis, L Fountoulis, A Fox, ZA Fozo, D Fra Corral, E Fradelos, G Fragulidis, A Fraile, A Francia, E Francis, S Francis, A Franco, NF Franco, P Francúz, A Frankel, R Franz, E Fraser, R Fratarcangeli, J Frazão, B Freire, NM Freitas Oliveira, K Fretwell, E Frimpong-Manso, F Frongia, D Fry, JH Fu, C Fuentes Orozco, L Fuentes Rivera Lau, AN Fuertes Muñoz, R Függer, Y Fulgence, C Fumagalli, N Furbetta, D Fusario, L Gabellini, O Gabom, T Gach, K Gaikwad, M Galasyuk, S Galati, G Galatioto, J Galea, F Galley, E Gallo, G Gallo, A Galvan, Y Galvañ Félix, B Gama, MDC Gama Caldeira, SL Gamba, M Gambelli, S Gananadha, L Gantner, DS Garcés Palacios, M Garcia, JS García, A García Domínguez, JL Garcia Galocha, S García López, D Garcia López De Goicoechea, A García Marín, EP Garcia Santos, MÁ García Ureña, JC Garcia Vera, P Garcia-Dubus, J García-Quijada, C García-Salas, G García-Santos, EY García-Villegas., L García-Zambrano, L Garciandia, F Gareb, S Gargiulo, M Garino, N Garmpis, IU Garzali, C Gas, MÁ Gascón Domínguez, I Gascon Ferrer, É Gáspár, S Gaspar Reis, J Gass, D Gavit, E Gavriil, A Gbeadese, G Gbessi, A Gegúndez Simón, K Geiger-Timm, MS Genç, V Gentilino, D George, G George, M George, PY George, F Georgiades, D Georgiev, V Georgilaki, R Geow, S Gerdes, D Gero, I Gerogiannis, A Gerundo, E Gessa, G Getachew, R Ghaleb, YSSM Ghaleb, A Ghanbari, A Gharib, S Ghattas, A Ghazal, A Ghazal, F Ghazali, A Ghummied, M Ghunaim, A Giakoustidis, D Giakoustidis, E Gialamas, KS Giannakopoulos, S Gilbert, A Gill, S Gill, J Gillingham, T Gimenez Maurel, E Gimson, C Ginesta, R Gioco, V Giordano, S Giovampietro, G Giraudo, S Giridaran, T Girma, JE Giubi Bobeda, S Giudici, M Giuffrida, A Giuliani, M Giuliano, D Giulitti, M Gjoka, S Gkogkos, A Gkouniaroudi, A Gkoutoula, P Glushkov, D Gobatti, S Gobishangar, J Goedeke, M Goga, I Gogoulis, N Gokhare Viswanath, A Goliath, S Göller, C Golliez, I Gomatos, F Gomes, M Gomes, J Gomez, S Gomez, D Gómez, E Gómez Mejía, T Gómez Sanz, H Gomez-Fernandez, DM Gonçalves Múrias Gomes, JG Goncalves-Nobre, VE Gonzabay, DS Gonzalez, MDC Gonzalez, C González, E González, JE Gonzalez Aboytes, C González Baez, M González De Miguel, J Gonzalez Garcia, E González Marín, A Gonzalez Ojeda, FY González Ponce, ME Gonzalez-Gonzalez, GR Goodwin, F Gool, S Gopaul, A Gori, M Gosselink, AG Goswami, I Gouazar, R Goudou, N Gouvas, S Govender, U Govindan, R Govindaraju, V Govindasamy, C Gracia-Roche, F Grama, M Gramellini, R Granata, V Granata, G Grande, M Grande, L Granero, G Grassi, G Grava, G Gravante, A Gray, G Graziano, L Green, K Griffin, S Grimaldi, M Gritti, P Grivas, P Grondona, J Grosek, U Grossi, R Grützmann, S Guadagni, S Guarriello, G Guercio, R Guercio, B Guerra, M Guerrero, J Guevara, C Guijarro Moreno, I Guirguis, RR Gujjuri, OB Gülcicek, CE Guldogan, A Gulla, T Gülşen, S Gumede, M Gumsheimer, M Gün, V Gunasaegaram, A Gupta, A Gupta, A Gupta, H Gupta, M Gupta, N Gupta, P Gupta, R Gupta, M Gureh, M Gurung, J Gusa, JB Gusa, A Gusibat, AE Gut, E Gutierrez, CE Gutierrez De La Rosa, JA Gutiérrez Gómez, JA Guzman Barba, R Guzmán Lambert, FE Gyamfi, A Gyedu, M H. Oweidat, R Habib, H Habiba, B Habrat, AD Hacioglu, C Hacıalioğlu, D Hackner, A Hada, F Haddad, F Haddad, R Haddad, D Hadzhiev, E Hadzhieva, E Haege, I Hagbevor, M Haghighat Ghahfarokhi, A Hai, A Haidar, E Haiden, MA Haider, Y Haido, M Haimona, H Haj Freej, M Haj Hussein, M Hajalamin, A Hajali, A Hajela, M Hajhamad, I Hajmohammed, S Halabi, E Halilović, K Hall, R Hall, M Hamada Takrouney, G Hamdan, H Hamdar, IHS Hamdun, E Hamed, M Hamed, A Hameed, BH Hameed, S Hammad, AR Hammadieh, A Hammed, F Hammett, M Hammoda, M Hamoud Almahly, I Hamzaoglu, R Handa, S Handa, S Hanna, D Hannedige, ZA Hannouneh, O Hany, BZ Hao, PW Haque, S Hariharan, C Harmston, M Harris, T Harris, N Harrison, G Hart, A Hasan, L Hasan, L Hasan, M Hasan, Y Hasan, A Hashmi, S Hassan, A Hassanin, FRH Hassooni, E Hatangimana, A Haty, R Hauwa Sani, L Havranek, A Hawarah, K Hayat, S Hayat, L Heeren, E Hegab, R Hegy, B Helou, C Henriques, S Henriques, D Herappe, MO Herdan, A Herewini, A Hernández, JR Hernández, BY Hernandez Cervantes, C Hernandez Diaz, RA Hernandez Rodriguez, D Herrera, R Herrera, JOM Herrera Batres, I Herrero, TK Hessou, TK Hessou , A Hewari, R Hiary, C Hidalgo Salinas, N Hielscher, L Hijazein, M Hijazi, A Hilder, D Hili, A Hiller, SS Hlaing, R Hleyhel, G Hneino, R Hodgson, AM Hodonou, E Hodžić, E Hodžić, K Hofmann, P Holt, J Hong, C Hoogerboord, E Hopping, M Horga, A Horiguchi, P Horvath, C Hossu, D Houmran, F Hounde, SFA Houndji, F Hourri, I Hoxhaj, E Hristova, A Hrora, Y Huang, CF Huaroto Landeo, F Huda, P Hudáč, TJ Hugh, S Hügli, A Huremovic, M Husain, S Husanov, N Hüser, R Hussam Yacoub Hattar, I Hussein, A Huws, J Hwang, A Iacomino, T Iahmo, M Ibadin, F Ibañez Ortiz, O Ibarra, SA Ibarra Camargo, TR Ibarra-Hurtado, GP Ibero Casadiego, B Ibi, TT Ibiyeye, B Ibrahim, D Ibrahim, H Ibrahim, M Ibrahim, O Ibrahim, S Ibrahim, A Ibrahim Hassan, S Ibrahim Tour Harakan, S Ideh, H Idheiraj, P Idjerhe, D Idowu, G Ietto, N Ildephonse, K Iles, K Iliakopoulos, I Ilieva, C Ilo, S Imam, L Imchen, I Imihteev, M Imirski, C Infante, M Infantes Ormad, S Ingley, SG Intini, A Ioannidis, O Ioannidis, V Ioannou, A Iodice, A Iqbal, M Iqreewi, FF Irakiza, H Irfan Khan, O Irowa, S Irrinki, A Irshad, E Irungu, M Ishak, M Ishak, A Ishola, H Islam, S Islam, A Isler, MA Ismael Alamin, N Ismaiel, MIS Ismail, S Ismail, M Issa, MI Issa, R Issa, A Issaka, H Iswariah, T Ivanov, BO Izedomi, A Izgiş, A Izzo, F Izzo, Arya J, Kavitha J B, R Jaba'Teh, M Jabal, D Jabbar, S Jabeen, A Jaber, H Jaber, W Jabłoński, H Jafarkhan, K Jaffry, J Jahjah, A Jain, D Jain, G Jalal, H Jalilehvand, C Jamieson-Grigg, S Jamil, M Janda, A Janjua, A Jarimba, S Jarmusch, K Jasaitis, K Jasso García, R Jayakrishnan, A Jayapalan, U Jayarajah, S Jayasekara, S Jayatilleke, M Jean Claude, N Jeannette, Y Jedidi, T Jemal, MR Jesudason, C Jezieniecki, AK Jha, VS Jha, N Jiagge, L Jiménez, JA Jimenez Flores, A Jimoh, A Jindal, A John, P John, A Johnson, B Johnson, Z Johnson, L Johnstone, M Jokubauskas, O Jolayemi, S Jomaa, J Jones, JJ Jordaan, TW Jorgensen, E Jose, A Joseph, S Joseph, D Joshi, D Joshiba, R Joumaa, L Jovcheski, N Jović, A Jowharji, S Jp, LC Juan Carlos, R Jubran, J Juloski, M Jurado Román, R Jurdon, O Jurić, Manojkumar K B, Manoj Kishan Lal KSD, A K. Ali, AZ Kaan, K Kabulov, MJ Kacem, S Kache, A Kachi, A Kadamani Abiyomaa, B Kadir, G Kafa, E Kafui Ayodeji, J Kahn, J Kaippally, M Kajic, V Kakotkin, M Kalabić, N Kalampokis, MC Kalaydjian, M Kalender, S Kalenga, AA Kalidis, M Kalisvaart, V Kalles, A Kalogeropoulou, R Kalouk, UA Kalu, JA Kalyanapu, A Kamal, H Kamal, M Kamal, M Kamal Matter, V Kamalathevan, M Kamar, S Kamari, K Kambouri, P Kamenova, E Kamer, C Kamphues, S Kanaan, P Kanavidis, I Kandil, L Kang, M Kang, V Kanna D, N Kansakar, U Kanyan Kassim, E Kapasakis, S Kapiris, M Kaple, G Kapogiannatos, P Kapsampelis, N Kapur, ME Kara, MA Kara, Y Kara, EC Karabulut, G Karadeniz Cakmak, C Karagianni, SN Karahan, T Karahasanoğlu, G Karakaidos, E Karakeke, M Karakeke, A KARAKOSTA, M Karamanliev, A Karamarkovic, ZR Karampotaki, S Karandikar, M Karanikas, Y Karataş, I Karatsolis, M Kariem, S Karim, N Karimbocus, S Karmarkar, N Karthikeyan, N Karunaratne, S Karuppusamy Krishnasamy, L Karydakis, D Kasasbeh, C Kaselas, P Kasetsermwiriya, W Kasetsermwiriya, M Kashif, S Kashyap, S Kassad, M Katif, H Kato, J Katogiritis, S Katorkin, S Katragadda, I Katsaros, L Katsiaras, L Katsiaras , A Katsiou, R Kattini, Z Katusic, G Kaur, H Kaur, H Kaur, H Kaur, M Kaur, P Kaur, R Kaur, R Kaur, S Kaur, M Kausar, D Kaushal, N Kavak, A Kavalakat, G Kavalieratos, AA Kayali, G Kazobinka, T Kebede, A Kedwany, D Kelgiorgi, M Kelly, A Kelzia, G Kenchadze, S Kenworthy, MD Keramida, IM Kereet, Y Kerolous, S Kessab, A Kezze, A Khair Etareig, A Khairy, A Khaity, M Khaled, K Khalid, M Khalid, MS Khalid, R Khalid, L Khalifa, M Khalifa, A Khalil, K Khalil, M Khalil, M Khalil, O Khalil, R Khalil, A Khalleefah, A Khamees, A Khan, B Khan, MF Khan, S Khan, S Khan, Z Khan, Z Khan, A Khanduri, S Khare, B Kharga, A Khatib, B Khattab, DH Khattab, S Khattak, AA Kheirkhah Vakilabad, T Khewater, N Kheyrbek, A Khoneisser, A Khouli, J Khoury, Z Khraim, S Khurana, K Khutsishvili, R Kibuuka, M Kießler, B Kigwe, C Killoran, A Kirschniak, M Kisielewski, R Kitamura, A Kiyabayev, MC Kizilkaya, N Kiziltoprak, N Kiziltoprak , N Klammer, J Kleeff, M Klib, Z Klib, C Kloppers, N Kłos, C Knee, SR Knight, MA Koc, MA Koç, AF Kocaay, P Koggoh, D Koike, G Kokkinos, P Kokoropoulos, C Kolla, VV Kollengode, M Kołomańska, LC Kolongi, DO Komolafe, K Komorowska, S Kondpan, D Kone, A Königsrainer, I Königsrainer, D Konkin, EDF Konlan, MK Konstantinidis, E Kontis, A Kontokostopoulos, K Kontzoglou, N Kopanakis, KC Kordeni, D Korkolis, C Korzin, ML Koschke, J Kosir, JA Košir, T Košir Božič, S Kosna, P Kostoglou, G Kostoulas, M Kotb, N Koter, P Kothari, D Kotsaris, R Kottayasamy Seenivasagam, G Koukoulis, MN Kouliou, K Koumarelas, G Kouraklis, C Kourouniotis, M Kouta, N Kouzakos, P Kowalewski, YO Koyluoglu, A Kozadinos, I Kozadinos, T Kozonis, R Kpangkpari, F Krämer, C Krautz, W Krawczyk, S Kreid, A Krishna, R Krishna Raj, N Krishnappa, K Kröning, M Kruščica, S Kshirsagar, E Kubiliute, GO Kucuk, V Kudoh, Y Kudryavcev, M Kulimbet, D Kulkarni, S Kulkarni, N Kullab, B Kum, A Kumar, A Kumar, A Kumar, M Kumar, N Kumar, P Kumar, R Kumar, S Kumar, V Kumar, S Kumar Venkatappa, S Kumaran, P Kumassah, U Kumbhar, Ö Küpçüoğlu, D Kurochka, GK Kurtoglu, GM Kurtoglu, GK Kurtoglugk, M Kusiński, J Kutkevičius, A Kutma, F Kuubetersob B. N, TY Kwan, M Kwon, N Kydonakis, I Kyei, M Kyereh, D Kyeremeh, S Kykalos, H Kynaston, F Kyramargios, A Kyriakidis, M Kyriazi, Bandareesh L, A L'Hostis, DKK Labadah, O Ladlow, L Laface, N Lahmer, N Lahnaoui, A Lai, WKH Lai, M Lainez Escribano, Y Lakdawala, S Lakew, N Lakhanov, P Lal, AK Lala, R Lalanda, N Lalovic, YH Lam, K Lambri, A Landaluce-Olavarria, I Laopeamthong, A Lapergola, P Lapolla, N Laquatra, P Laranjo, A Lareida, C Larotonda, R Laskowski, C Lategan, SG Laura, S Laura, S Lauricella, AO Lawal, A Lawal, A Lawal, TA Lawal, S Lawday, M Lazo Ramírez, H Le Roux, C Leal Ferrandis, H Lederhuber, DN Lee, G Lee, K Lee, LD Lee, S Lee, K Lehmann, N Lekiashvili, L Lely, A Leon-Del-Angel, FJ León Frutos, C León-Espinoza, NN Leonelle Lore, J Leong, R Lertnamvongwan, C Leung, P Leungon, P Levíček, A Li, C Li, MR Li Valencia, R Liang, SC Liapis, A Liaquat, L Licari, A Licciardello, H Lidbetter, M Lie, L Liepa, M Lill, A Lim, A Lim, CK Lim, JH Lim, O Lima Azurdia, P Limani, C Limas, A Lin, V Lin, EP Lincango, J Lindert, N Lindi, J Linker, C Lionel, O Lisin, A Litchinko, D Liu, V Liu, V Lizzi, T Lo, LB Lo Piccolo, E Locci, M Lodha, N Lodhi, MW Löffler, A Loizou, SN Loke, A Lombardero, T Longkumer, R Lopatriello, B Lopes Patrício, NE López Bernal, E López-Negrete Cueto, Z Lorenc, E Lori, M Lotfy, AR Lourenço, IM Lourenço, B Louro, A Lovi, D Lowen, EE Lozada Hernandez, Y Lu, M Lucchini , F Lucero, S Luciano, G Luglio, A Lukáč, H Lule, B Lulham-Robinson, E Lun, EWY Lun, R Lunevicius, CI Lupercio Figueroa, A Luther, KK Luthra, M Luthra, A Luzzi, P Lykoudis, D Lytras, Srikanth M, L M Mheidat, Yashas M S, T M. Abubasheer, D M. Awad, B M. J. Alhaj, M M. Nathan, S M. Udwan, M Maak, D Maan, B Maanikuu, T Mabogoane, W Mabood, C Macedo Cardoso De Oliveira, N Machairas, A Mačiulaitytė, R Mackay, J Mackenzie, S Mackenzie, M Maclean, MEM Madany, R Madi, Y Maestre González, F Maffei, F Maffeis, R Magarini, A Maghrabi, S Magrhi, B Maguire, M Mahadi, M Mahafdah, OR Mahafdah, H Mahajna, C Mahakalkar, K Maharaj, I Maharem, N Maharjan, S Mahdi, SN Mahendra, A Mahendran, V Mahendravarman, A Maher, S Mahfoud, N Mahmood, F Mahmoud, F Mahnic, M Mahran, JV Maida, T Majed, L Majerčák, JG Makama, D Makary, ST Makkai-Popa, Y Maktabi, R Malatesti, S Malek, SS Malhi, AA Malik, NA Malik, H Malkawi, Y Malki, D Mallik, G Maltinti, S Mamduh, G Mammolo, M Manangi, F Manasci, DK Manatakis, V Manchev, A Manetti, M Manfredini, T Manickam, Y Manickchund, M Manigrasso, P Manikis, P Manjunath, L Manoj Joshua, M Mansoor Iqbal, A Mansour, AA Mansour, E Mansour, L Mansour, M Mansour, O Mansour, S Mansour, SV Manzoor, B Mao, S Maqbool, S Maqsood, A Maqsood-Shah, C Marafante, A Marano, A Maraqa, S Marawu, MK Marawy, M Marcianò, N Marcos, MMA Marei, A Marello, R Mares País, I Margaris, E Margelis, C Margiani, M Mariani, FAN Marin, GMF Marini, A Marinis, V Mariottini, J Maritz, C Markin, B Markowska, R Marlin, G Marom, P Marongiu, A Marouf, I Maroulis, J Marques Antunes, M Marqueta De Salas, L Marquez, E Marra, J Martellucci, E Martí Cuñat, MdM Martí-Ejarque, B Martin, J Martin Fernandez, MP Martin Gimenez, O Martin Sole, L Martinek, L Martinez, MJ Martínez, MN Martínez Bareiro, R Martínez Díaz, A Martinez German, G Martínez Izquierdo, JF Martinez Martin Del Campo, M Martorana, JN Marx, S Marzorati, N Marzouqa, M Masaaod, H Masalma, A Masciandaro, Q Mashlah, M Masood, M Masoud, R Masri, ST Massa, D Massaras, D Massaro, G Massarra, P Masseria, IM Matache, O Matar, M Mateu, AR Mateus Loureiro, A Mathew, S Mathew, NN Mathioudakis, Z Matkovic, G Matroud, S Matthiess, M Mattis, M Matyja, N Maulenov, J Mavrek, M Mavri, E Mavrodimitraki, A Maxwell, C Maya, M Maybury, W Mayo, E Mazumdar, R Mazurkiewicz, F Mazzola, A Mazzoni, E Mazzotta, E Mbanzabugabo, JC Mbonicura, R Mcclen, S Mcclintock, B Mcdermott, R McGee, M McGuinness, B Mckay, K McKenzie, J Mcnab-Hand, A Md Yunos, F Medas, MJ Medina, SP Meena, S Megdiche, MEA Meghaizerou, S Megna, M Mehanny, M Mehmood, A Mehta, Y Melkamu, MP Mellado Tellez, M Melouane, N Memos, J Mendes, G Mendiola, S Meneghini, B Mengesha, D Mengiste, P Mensah, S Mensah, C Meola, F Mercado Sanchez, M Mercurio, S Meriç, D Merlicco, D Merlini, M Merrakos, M Mesfin, YY Metaferia, J Metzger, G Mevognon, S Mewa Kinoo, H Mftah, R Michael, V Michael, N Michalopoulos, J Michel, S Midya, A Miele, M Mietła, A Migdanis, J Mihanovic, S Mikalauskas, D Mikami, I Mikulic, M Milentijević, PJ Milewski, L Milic, J Miller, M Milone, HA Mimouni, E Mina, AM Minaya Bravo, A Mingoli, Y Mintz, R Miranda Pera, MB Mirza, D Misca, A Mishra, KK Mishra, TS Mishra, J Miskovic, D Mitchell, A Mitsala, R Mittal, H Miyasika, M Mobolaji-Ojibara, V Modekwe, MM Modolo, I Mogárrio, TG Moges, E Moggia, M Mogiatti, T Mogne, SF Moh Pauzi, B Mohamad, M Mohamad Amin, E Mohamed, O Mohamed, Y Mohamed, Y Mohamed, A Mohamed Ibrahim Mohamed, O Mohamed Mokbel, M Mohamedshafee, M Mohammad, J Mohammad Mohammad, H Mohammadi Sardoo, A Mohammed, A Mohammed, E Mohammed, KMG Mohammed, L Mohammed, MMH Mohammed, S Mohammed, T Mohammed, U Mohammed Bello, FA Mohammed Daoud, M MohammedAli, D Mohanty, MS Mohd Shah, A Mohsen, G Moitzi, M Mokhtar, Á Molero, C Molewa, S Molfino, JD Molina Marin, M Molteni, M Momoh, E Monati, A Moncy, I Mondi, J Mondino, FA Monib, L Moniz, S Monkhouse, M Montagna, E Montalbán Martínez, E Montanari, O Montaño Angeles, AG Montaser, L Monteiro, J Montero García, S Montisci, D Montwedi, P Moodley, L Moolla, A Morad, J Morales, S Morales, J Moravik, L Moreira, L Morelli, A Morello, M Moreno Gijon, MA Moreno Gonzales, C Moreno-Licea, O Morgan, K Mori, V Morinelli, D Moris, M Morjan, MT Morna, D Moro-Valdezate, N Morricone, N Mosbah, C Mosca, V Mosca, S Moschella, N Mosleh, MS Mosquera Paz, A Mostafa, M Mostafa, M Mostafa, S Mostafa Yassin, T Motiwala, A Mouffokes, R Moukarzel, MB Mourato, A Mourtzouni, V Mousafeiris, HB Moya- Ambriz, M Mozel, C Mpirimbanyi, I Mrazkova, MR Mslmani, M Mubarak, I Mubezi, AB Muhammad, S Muhammad, DA Muhie, J Mühlhäusser, M Muir, L Mukamazera, F Mulita, S Mungo, D Muñoz, ME Muñoz Fernández, N Muñoz Montes, AM Muntaka, R Munyaneza, G Munzi, M Muragi, V Muralidharan, M Muresan, ME Muriel, EMA Murphy, I Murshed, A Murtada, V Murzi, N Musawa, K Muscat, D Muschitiello, Z Musilová, A Mustafa, A Mustafa, B Mustapha, AB Muthunayagam, D Mutter, D Muyenzi, A Mwanjoka, AM Myintmo, Y Myla, IC Mylona, N Naabo, MH Nabhan, A Nabil, EA Nachelleh, A Nada, T Nadeem, I Nadi, TN Nagwamutse, G Nagy, K Nahar, E Naidoo, R Naidoo, U Naidoo, S Naidu, A Nair, S Nair, VV Nair, J Najajra, P Nambala, K Nanayakkara, M Nandasena, MA Ñañez, J Napoli, N Narain, JR Naranjo Fernández, C Narayan, M Narayanan, B Nardo, AE Narin, M Nasani, PR Nashidengo, I Nasser, K Nassim, C Nastos, F Natali, R Nataraja, G Natchagande, M Natey, R Nathani, A Natili, V Naunova, AM Nava Franco, A Navarrete-Peón, Ł Nawacki, A Nawawi, S Nayak, F Nazareth, E Ndizeye, O Ndizeye, B Neall, I Negoi, VM Negoita, CN Nelly Rosine, S Neogi, Y Nerabani, I Neri, R Nesco, LC Nespoli, A Neves, V Neykov, J Ng, S Ng, SHM Ng, V Ng, J Ng-Kamstra, A Nguyen, A Niazi, GD Nibogora, C Nicholson Jr., A Nidali, M Nieto Galvan, K Nieto Yrigoin, K Nieuwenhuys, NS Nik Burhanuddin, N Nikam, M Nikberg, A Nikitaras, AI Nikolaou, C Nikolaou, V Nikolaou, B Nimako, C Nimbona, A Ningi, GE Nita, AF Nixon Fulli, L Niyidukunda, I Njere, M Nkogatse, M Nkoronko, G Nkunguzi, M Nnabagulanyi, J Nnoli, M Nofal, S Nofal, K Noguchi, M Noguez Castillo, R Noor, I Norman, M Nortey, O Nouhail, H Novák, S Novello, C Ntagkas, N Ntanzi, K Ntatsis, P Ntem, CK Ntow-Boahen, G Ntwari, OG Nubi, EG Nubi Mohamed, M Nunes, M Nunes Luís, H Nuñez Del Barrio, A Nuñez Venzor, CM Nuño-Guzmán, SE Nwabuoku, C Nwachukwu, N Nwafulume, EI Nwangwu, C Nwanmah, N Nwanne, C Nwegbu, C Nwokoro, C Nwosu, CD Nwosu, J Nyamekye-Baidoo, C Nyampinga, F Nyarko, M Nycz, M Nyirenda, R Nyirenda, PC Nze Obiang, IC Nzenwa, E O'Connell, E O'Neill, M Obaid, A Obbeng, I Obianyo, EA Obiesie, NKA Obuobi, HO Odah Bashi, Z Odeh, KE Oderoha, V Odigie, KB Oduro-Boateng, EO Ofori, J Ofori, D Ofosuhene, I Ogundele, H Ogundipe, S Ogunlade, O Ogunsua, K Oğur, S Ohri, A Ojewuyi, O Ojewuyi, A Ojo, M Ojo, O Ojo, A Oke, M Okechukwu, J Okei, I Okoro, OA Okoye, O Okrah, M Okudero, AI Okunlola, A Olabode, O Olajide, O Olajumoke, F Olaniru, J Olaogun, O Olasehinde, R Olatunji, S Oliveira, M Olivos, MDM Olmedo Reinoso, T Olobatoke, N Oloko, S Olori, J Olorunfunmi, A Olsen, K Oluchukwu, AM Olugbami, O Oluseye, O Oluyemi, D Omar, M Omar, M Omar, S Omar, U Omara, M Omer, N Omer, D Ommi, O Omoike, L Omomeji, P Omwansa, M Oncel, F Ondago, HMA Oneizah, CS Ong, A Ongaro, V Ongil Rodríguez, F Oni, C Onuoha, C Onwuzu, L Onyebulu, C Ónyeka, K Onyekachi, K Ooi, R Ooi, AN Oommen, A Oosterkamp, F Opoku Twene, A Opoku-Agyapong, A Oppong, J Oppong, R Oppong, R Oppong-Amoah, Y Orabi, I Orji, G Orlando, JE Orozco Navarro, J Orozco-Perez, M Ortega Escudero, L Ortega Lechuga, Z Orzeszko, O Osagie, Z Osama, E Osaze, N Oscar, DB Osei, EO Osei, D Osifo, C Osime, F Osman, I Osman, M Osman, O Oso, V Osoka, M Osso, P Ostruszka, C Osuigwe, O Osunlusi, S Otho, J Otote, A Ottaiano, L Ottaviani, A Ouachhou, N Ouachou, Y Ouadi, S Oum, A Ousseini, P Owens, O Owolanke, F Owusu, G Owusu, B Owusu Ansah, A Oyedele, AAA Oyelekan, N Oyelowo, T Oyeyemi, H Oyinlola, O Oyinloye, MA Oyortey, D Ozal, İH Özata, A Özcan, B Özcan, MF Ozcelik, K Özdoğan, OF Ozkan, C Özkan, A Ozkomec, E Ozoran, H Ozsahin, MS Ozsoy, AM Öztürk, S Öztürk, G Ozyuksel, Sagar P, Induchoodan P S, M Pacilli, M Pacilli, T Padioti, N Paez, M Pagani, E Page-Taylor, D Paglione, N Pahuja, HS Pahwa, D Pais, R Pajtak, AK Pal, RM Palacios Huatuco, N Palamara, M Palas, P Palazon Bellver, DA Palma Portillo, M Palmeri, F Palmieri, G Palmieri, D Palmisano, G Palomba, F Palomino Escalante, P Palumbo, A Panagakis, A Panagidis, A Panagopoulos, D Panagopoulos, P Panahi, L Pánči, V Panduro-Correa, S Pandya, H Panga, C Panis, M Pannullo, DA Pantoja Pachajoa, I Papaconstantinou, N Papadogianni, M Papadoliopoulou, A Papadopoulos, V Papadopoulos, D Papageorgouli, E Papamattheou, M Papamichail, C Papazacharias, X Papazarkadas, G Pappas Gogos, P Paraskeva, J Paredes, L Paredes, M Park, N Parker, R Parker, T Parker, C Parmar, FQ Parray, A Parseliunas, I Parwaiz, G Pascarella, FR Pascual, A Paspala, N Pasqua, C Pastás, A Patel, P Patel, P Patel, P Patel, S Paterna-Lopez, L Paterson, M Pathak, SR Pathan, DC Patiño García, S Patrocínio, R Patrone, D Patterson, B Paul, N Paul Ambrose, NP Paul Sigamony, K Pavlopoulos, G Pavone, S Payyanur Thotan, A Peckham-Cooper, F Pederiva, H Pehlivan, M Pejović, LÁ Pelayo Orozco, JB Pellecer Cano, M Pellen, GA Pellicano', G Pellino, LG Peña Balboa, MJ Peña Soria, J Pereira-Macedo, M Perera, R Perera, M Perez, R Perez, Y Perez, CJ Pérez-Padrón, HDJ Pérez Baca, DA Pérez Muñoz, CJ Perez Rivera, RH Perez-Soto, MR Peris, K Perivoliotis, A Perja, M Perkins, G Peros, S Perretta, D Pertile, AL Pesce, A Pesce, L Petashvili, CT Petcu, B Peter, M Peter, F Peters, L Peters, N Petrarota, K Petrenko, J Petric, T Petropoulou, A Petrungaro, W Petrushnko, B Phakathi, M Pham, TV Pham, O Pheiffer, R Philip Sridhar, M Philipp, F Philips, D Phom, MG Piacentini, M Piccino, SA Piccioni, MC Pignanelli, G Pignata, D Pignatelli, A Pikarsky, A Pilavas, A Pilavas, T Pilia, A Pillai, G Pillai, V Pillay, S Pimentel Morais, R Pinheiro Duque, AM Pinheiro Pereira, J Pinho, A Pinto, B Pinto, I Pipia, C Piras, D Pironi, M Pisano, M Pisano, A Pisanu, G Pisarevi, A Pita, M Pitiakoudis, I Pius Ogolekwu, J Pizarro Lozano, A Plastiras, H Pleass, XY Po, M Podda, L Poggi, L Poggi, G Poillucci, S Polat, A Polatbekov, A Police, D Politis, R Polo, K Polychronopoulos, E Pontecorvi, O Popoola, F Porcelli, M Portelli, E Potenza, GE Poto, G Pou, E Poulios, M Pourfridoni, D Powell, HK Prabhakar, PS Prabhu, L Pramod, Arun Prasath S V, J Prat-Ortells, N Pratas, AL Preto Barreira, D Prevezanos, F Prieto La Noire, P Probst, G Procaccini, MP Proclamà, D Proud, M Prudhomme, O Pryer, M Pryt, J Psaila, I Psilopatis, S Puddu, C Pun, A Punzengruber, A Qasem, H Qayum, J Quansah, M Quante, S Quartarone, M Quaunine, FW Quayson, A Quddus, C Quintela, K Quispe De La Roca, S Quoy, AU Qureshi, Z Qureshi, S Qwyder, B R Budihal, I R Fakhradiyev, R Rabah, N Rabai, D Rabaia, R Raddad, B Radja, R Radojković, G Rados, R Radwan, S Rafiq, MN Rafique, A Ragab, L Ragazzini, A Ragheb, R Raghunath, K Raghuwanshi, CF Rahantasoa Finaritra, M Raheem, R Raheem Attallah Al_obaidy, A Raheja, A Rahman, GA Rahman, KR Rahman, D Rahme, L Rai, N Raiq, N Rajab, K Rajaratnam, S Rajendra, D Rajput, MJ Rakotonaivo, A Rakotondrainibe, I Rakshit, I Rakvin, F Ramalho De Almeida, M Ramazanov, R Ramchandani, A Ramírez Beas, RA Ramirez Calas, S Ramjit, NN Ramli, AN Ramly, A Ramos Bonilla, L Rampersad, RR Ranadive, B Ranjous, M Ransome, C Raphael, A Rashad Temerik, A Rashid, T Rashid, F Rasoaherinomenjanahary, N Rasool, V Ratheesh, J Rathod, KJ Rathod, D Rattray, F Rauf, S Raul, N Rawal, T Rawther, O Ray, M Rayzah, A Raza, YM Razafimandimby, JB Razafindrahita, M Razaq, S Razaq, A Razzore, F Ré, LA Rea Bocanegra, M Reda, KMR Reddy, SM Reddy, R Redkar, E Redondo, FJ Redondo Calvo, M Redynk, R Refaie, D Rega, P Rego Ponte, WU Rehman, B Rehmani, M Reia, T Reichelt, E Reid, H Reilly, J Reilly, D Reim, M Reis, E Reitano, K Rekouna, A Rencuzogullari, L Rende, S Rennie, M Rennis, N Renovat, L Resca, B Révérien, DE Reyes Rodríguez, DH Riazhussain, M Riba Martínez, J Ribeiro, J Ribeiro, R Ribeiro, R Ribeiro Dias, D Ribero, C Riboni, J Ricardo, V Ricchiuti, G Riccioli, HE Rice, K Richetti, K Richter, D Ridder, P Riedl, C Righetti, J Ringers, C Rio Ferreira, L Ripamonti, R Ripoll I Palmés, F Ris, T Risteski, M Rius, P Riva, R Rivas, U Rivolta, R Roberts, Z Robertson, B Robertson-Jones, A Robin Valle De Lersundi, L Robine-Durnell, SJA Robinson, S Robinson, RJ Robitsek, MS Rodha, JL Rodicio Miravalles, CS Rodrigues, L Rodrigues Madeira, L Rodríguez Gómez, A Rodriguez Gonzalez, M Rodriguez-Lopez, M Rodríguez-Ordoñez, R Rodríguez-Uria, A Rojas, J Rolinger, C Rolo Santos, E Romairone, J Roman, CF Roman Ortega, S Romano, A Romero De Diego, G Romero Reyna, EV Romo Ascencio, FD Romo Rosales, U Ronellenfitsch, F Roodt, B Roopavathana. S, D Rosado, A Roslani, L Rossi, M Rottoli, N Roukounakis, A Roumieh, E Roussos, M Roussos, F Roviello, P Royo Dachary, L Rubin, R Ruccella, F Rudisch, R Rüdrich, MJ Rueda Medécigo, F Ruescas, M Ruffoli, JP Rugambwa, VA Ruiz López, M Ruiz Soriano, E Ruiz-Daum, L Ruiz-Villa, D Rukavina, C Rukundo, IS Russo, N S.Bayleyegn, MM Saad, A Saba, A Sabbah, Q Sabbah, S Sabbatini, M Sabboh, H Sabir, T Sabra, M Sabri Massadi, A Sabry, MD Sacdalan, M Sadek, I Sadiq, MA Sadiq, MH Sadiq, H Sagar, R Sagoe, L Sahan, H Said, M Said, S Said, M Saidani, M Saifi, N Saini, A Sainz Lete, E Saitoglou, Y Sakaray, P Sakarellos, A Sakr, M Salah, M Salah, O Salako, A Salam, S Salama, G Salamone, P Salas Núñez, A Salazar, TA Salazar-Lorenzana, M Saleem, A Saleh, M Saleh, M Saleh Khatab, G Saletta, N Salgadoe, M Salibašić, S Salido, R Salim, S Salim, AA Salinas Barragan, S Salindera, D Salinovic, E Salloum, S Samadi, E Samara, D Samardali, S Samardali, Y Samer Morsy, L Samison, M Sammut, M Sammut, C Samojeden, D Sampanis, R Sampietro, M San, A Sanad, MA Sánchez Audelo, N Sánchez Fuentes, A Sanchez Gallego, A Sánchez Gollarte, M Sánchez Ramirez, M Sánchez-Rubio, AI Sánchez-Terán, P Sancho Pardo, SA Sani, AN Sanli, AST Sanon, AF Sanon, A Sanou, R Santana Ortiz, M Santarelli, L Sante Serna, M Santillan, G Santos, I Santos, M Santos, R Santos Pereira, W Santucci, S Sanz, A Sapmaz, MW Saqib, A Sarafi, A Sarakatsanos, B Sarang, M Sarda, O Sarhan, A Saridaki, AC Sarı, MAK Sarker, G Sarp, Y Sarpong, SH Sarwary, D Sasia, R Sato, S Saudí-Moro, J Saunders, C Saviello, KA Sawaftah, MA Sawaftah, MAM Sawaftah, MN Sawas, G Saxena, R Saxena, R Sayad, A Scacchi, L Scaravilli, M Scheiterle, A Scheiwiller, J Schembri Higgans, N Schiavon, L Schiavone, S Schimpke, D Schippers, V Schirinzi, S Schirone, D Schizas, E Schmidt, R Schmidt-Branden, MA Schneider, M Schön, M Schüler, G Scialandrone, R Scola, F Scolari, M Scopelliti, M Scortechini, B Scotto, D Scotto Di Carlo, M Scricciolo, Y Seada, A Sebai, M Sebastián-Mendoza, J Secchi, N Seeger, C Seet, S Segnitome, B Sehgal, AS Seidu, C Seiler, MI Seixo, ME Seker, S Selim, S Sellahewa, F Selvaggi, C Semeraro, YK Şen, KJ Senanayake, J Senavirathna, C Seneza, R Senin, Z Şenol, I Separovic, E Sepúlveda, L Sequi, A Serao, W Serednicki, I Serfontein, M Şermet, V Serna-Alarcon, I Serrano, P Serrano Méndez, M Sersarah, BK Seshie, MK Sethi, R Sethi, M Seto, E Setsoafia, S Severi, K Sevnaran, G Seyfu, J Seyi-Olajide, G Sgarzini, Z Shabello, A Shabkah, A Shah, ZA Shah, I Shahbaz, A Shaheen, HH Shahid, M Shahid, M Shakhshir, M Shalaby, M Shalaby, M Shalkamy, H Shames, KA Shamiyah, L Shammas, M Shams, G Shamsi, A Shamsutdinova, A Shanker, S Shanker, A Shanmugalingam, B Sharaf Eldin, I Shariff, D Sharl Ajami, A Sharma, A Sharma, K Sharma, M Sharma, N Sharma, N Sharma, RD Sharma, R Sharma, V Sharma, A Sharp, AA Sharq, L Shawesh, I Shehadeh, N Shehata, C Shelton, K Shemyatovsky, D Shen, R Shen, H Sherif Farouk Ahmed Hassan, M Sheriff, AA Sheshe, E Shestakov, P Shimbulu, C Shiraishi Zapata, S Shittu, P Shivashankar, S Shivashankar Chikkanayakanahalli, K Shodunke, A Shoker, TT Sholadoye, MD Shoshkova, K Shreyas, P Shukla, N Shulman, K Shwail, A Shweiki, I Siannis, S Sibiya, J Siby, G Sica, M Siddique, T Sidiropoulos, A Sidorova, J Sidorovskaia, V Siepaal, S Sierra, E Signaroli, E Silanos, A Silva, C Silva, D Silva, R Silva Borges, FJ Silva Rivera, DW Silva-Cano, S Silvestre, V Silvestri, KK Sim, B Sime, S Simeonidis, J Simoes, L Simonelli, G Singer, A Singh, D Singh, G Singh, G Singh, I Singh, K Singh, M Singh, M Singh, M Singh, MP Singh, SK Singh, S Singh, S Singh, TP Singh, V Singh, A Sinha, A Sinha, R Sinha, A Sinjab, L Siragusa, D Siriwardena, AC Sironi, J Siu, J Skaff, A Skarpas, H Škiljo, A Skreka, G Skroubis, M Slavchev, C Smart, G Smith, K Smith, S Smith, RL Smolinski Kurek, A Smyrnis, C Soares-Aquino, C Soddu, S Soelling, F Soggiu, R Soglonou, M Sohn, R Sokratous, L Solaini, N Soldo, R Solecki, F Solimene, R Soliva Domínguez, M Solkar, N Solomon, E Somuncu, AA Sonkar, RE Sönmez, RE Sönmez , T Soric, D Soriero, P Sornoza, S Sorrenti, C Sorrentino, G Sorrentino, ED Sosa Ferreira, DA Sosa Méndez, MA Sotiriou, M Sotiropoulou, S Soto Schütte, ÓA Soublett Rivas, I Sougkas, A Soumpasis, R Souto, E Soyer Güldoğan, E Spalice, F Spanos, E Spanoudakis, A Sparke, E Spartalis, M Spartalis, K Spassov, A Spaziani, R Spence, B Sperotto, N Spiteri, I Spyridakis, N Srinivas, S Srishankar, M Srour, S Staight, P Stamopoulos, N Stamos, H Stark, F Stavratis, GA Stavrou, G Steenkamp, C Stefanou, M Steffani, F Stefou, T Stefura, L Stella, C Stennard, N Steve, S Stevens, M Stjepanovic, s Stock, D Stoian, A Stollberg, S Stonelake, S Strachan, A Strumia, AA Suárez Álvarez, D Subasinghe, S Subbiah Nagaraj, A Subocius, R Sucher, S Sucu, R Sugair, A Sukhdev Jadhav, P Sukhvibul, A Sukumar, R Sulce, M Sulciner, F Suldrup, I Suleiman, IE Suleiman, R Suleiman, J Suleiman Hadidi, M Suleman, J Suliman, MA Suliman, F Sulo, A Sultana, A Sumbaev, A Sundaram, G Sundaram Venkatesan, S Sundaramurthy, S Sundararajan, S Surendran, P Suresh, S Suresh, AL Surkitt, A Suroy, J Sutcliffe, V Sutharshan, B Sv, L Sweidan, M Święch, AN Syed, A Syllaios, E Synekidou, A Szpytko, M Szura, G Szydlo Shein, D T. Enti, P Taah-Amoako, M Taamreh, N Tabatabaian, M Tabaza, AJ Tabiim, J Tabora-Zepeda, M Taeme, L Tafesh, M Tageldin, K Taghavi, L Taglietti, I Tagreda, H Taher, A Tahir, MA Tahir, W Tahir, F Tahir Lwdie, J Taiwo, A Talabi, N Talat, L Talé-Rosales, L Tallon-Aguilar, DP Talreja, K Tambudze, N Tamini, ZF Tamou, EC Tampaki, A Tampakis, E Tan, S Tan, M Tanal, Y Tanas, I Tanase, M Tanashat, YI Tandoğan, C Tandup, P Tang, A Tansawet, L Tapia Moral, G Tarantino, MF Tarar, A Tariq, X Tarrado, N Tartaglia, N Tasis, K Tata, F Tauheed, D Tavares, JA Tavares-Ortega, S Tawfik, M Tayyab, N Techapongsatorn, S Techapongsatorn, T Techapongsatorn, JST Tefay, PS Tekam Wadje, H Telci, M Tello Jimenez, V Ten, RA Tenfen Carneiro, F Terefe, M Teressa, ED Terzi, I Tesfahun, A Teshome Sahilemariam, H Tetley, SPB Thalgaspitiya, P Thambi, A Thanasa, T Theivendrampillai, SA Theivendran, V Themelidi, A Theochari, M Theodoridou, K Theodoropoulou, T Theodosopoulos, N Theophile, L Theuil, RS Thind, A Thomas, PA Thomas, C Thrasyvoulou, SI Thuraisamy Sarma, A Tibude, F Tierenye, I Tierris, S Timmalah, S Tingle, B Tinoco, F Tirelli, A Tirkey, R Titcombe, J Tk, SR Tobome, F Tofani, B Togay, ACS Toi, E Tokidis, M Tokocin, MA Tolani, EA Toma, A Tomazic, M Tomlinson, S Tontus, T Tony V, S Toprak, MMS Tora, A Torbey, LV Torres Bavestrello, EM Torres De Anda, ST Torres Rodríguez, A Torroella, Y Tosun, Z Toutounji, N Trabulsi, P Trakosari, N Tran, G Travaglini, SL Trejo Ramos, M Trejo-Avila, TDB Tridip, A Trif, A Trigos Díaz, M Trindade, P Trinity, R Tripathi, FP Tropeano, SA Trujillo Ponce, I Tsakiridis, N Tsakiridis, C Tsalikidis, N Tsantikos, V Tsaousis, S Tsatsos, S Tsatsu, P Tschann, A Tsechpenakis, V Tselepidis, A Tsiaka, D Tsiardas, F Tsige, T Tsirlis, M Tsopozidi, SM Tsoti, G Tsoulfas, M Tsuruta, L Tulinsky, H Tümer, R Tummon, R Tumolo, E Tunçcan, C Tung, KY Türker, S Turkmani, TB Türkmen, A Tursunovic, R Tutino, M Tutton, E Tuzuner, A Tvaladze, A Twumasi, H Tyagi, E Tyurina, P Ubiali, R Ubiñas, C Uche, J Ugwu, I Ugwueke, C Ugwunne, T Uiyapat, JJ Ulloa Robles, H Ulman, SS Uludağ, N Umar, CW Un, U Una, E Unal, H Unwin, TK Uprak, K Urbańska, G Urdang, M Usama, M Usman Malik, N Uttam, C Uwakunda, MS Uyanik, DS Uymaz, J Uys, AM Uysal, MY Uzunoglu, C Vacca, X Vagena, M Vailas, J Vaitekūnas, R Vakil, F Valente Costa Pinto, JI Valenzuela, Z Valera Sanchez, S Valeri, T Valizadeh Elizeh, L Van Den Hil, M Van Der Colf, J Van Niekerk, B Van Zyl, D Vardakostas, A Varga, C Varghese, R Varshney, M Vasconcelos, Y Vashishth, S Vasquez, P Vásquez, C Vasquez Maya, F Vassallo, P Vassiliu, J Vaz, D Vaz Acosta, R Vaz Pereira, A Vazquez Fernandez, A Vazquez Melero, SJ Vázquez-Sánchez, S Vederaki, A Veira, J Velkoski, F Velluti, S Vembar, L Venclauskas, D Venskutonis, P Venturelli, C Vera Mansilla, D Verdi, SS Verhage, S Vernadakis, M Veroux, G Verras, L Verre, S Vertaldi, NA Ververidis, A Vezakis, A Viacava, M Vicario Bravo, A Victor, I Vidić, B Vieira, JP Vieira De Sousa, A Vig, A Vilar, R Villalobos Mori, SA Villeda, JF Viñas, GR Viscido, D Visconti, C Vitiello, N Vlachakos, P Vladova, E Volpin, K Voon, V Vougas, F Vovola, A Vricheva, A Vu, J Vu, D Vukosav, K Waddell, P Waghchoure, H Wain, G Waiyaki, M Walędziak, D Waleed, A Walfor, DS Walia, P Walker, E Wallner, J Walshaw, B Wang, J Wang, J Wang, R Wani, SH Waqar, S Ward, M Waseem, I Wasiu, A Wassouf, M Watson, C Weadick, GF Weber, M Weber, O Webster, M West, O Whitehurst, M Wichmann, D Wickramarathna, D Wickramasinghe, F Wiese, C Wiesner, W Wijenayake, K Wijesinghe, M Wikar, A Wilkins, A Williams, B Williams, OM Williams, H Willy-Chidire, M Wilson, P Wilson, M Windsouri, U Wirth, T Wojewoda, J Woleský, V Wolfschluckner, E Wong, J Wong, ZY Wong, ST Workineh, M Worku, S Wrenn, D Wright, W Wysocki, H Xiao, B Yacoub, H Yaghmour, A Yahaya, M Yahaya, M Yahmad, A Yakubu, O Yalkın, K Yalley, W Yang, C Yanowsky-Gonzalez, G Yanowsky-Reyes, G Yarovenko, AC Yaşar, M Yashar, L Yasin, MM Yassin, S Yassin, E Yavuz, IE Yavuz, P Yeboah Owusu, EMT Yenli, E Yeshialem, A Yeshitila, AB Yevide, WT Yew, E Yhoshu, A Yiallourou, M Yigah, B Yigit, D Yigit, D Yiğit, H Yigitbas, S Yigman, EO Yildirim, A Yildiz, S Yilmaz, A Yingess, M Yinusa, S Yılmaz, TE Yılmaz, G Yohanna Abrak, H Yonekura, J Yorke, I Yotsov, T Yotsov, J You, E Younes, A Younis, H Younus, R Yousef Yassin, N Yousefzadeh Kandevani, MMT Youssef, E Yousuf, KKA Yu, P Yuide, S Yumurtacilar, H Yunes, MA Yunus, B Yunusa, SA Yunusa, C Yurttas, S Yussif, M Zaazou, B Żaczek, A Zafar, M Zahed Abdalla, ZA Zaher, M Zahran, H Zaigham, M Zaigham, HHZ Zaini, JK Zajac, AD Zakaria, K Zakkas, A Zamareh, M Zambon, M Zammit Vincenti, N Zampitis, ÖP Zanbak Mutlu, C Zandonella, G Zanni, G Zanus, F Zapata, C Zapata Syro, K Zapsalis, A Zarafidou, MÁ Zaragoza Mendieta, MF Zarate Casas, M Zawadzki, G Zaza, L Zeng, A Zerbinati, D Zerbo, WA Zerefa, G Zeringa, R Zerna Encalada, I Zerrouq, JC Zevallos-Quiroz, E Zhang, S Zhang, J Zhu, E Zidan, P Zimmermann, M Zounon, S Zourntou, A Zubillaga-Mares, V Zucchini, V Žufić, D Zuikyte, D Zulian, Y Zwain, H Zwaraa.
