# Correction to: Global access to technologies to support safe and effective inguinal hernia surgery: A prospective, international cohort study

**DOI:** 10.1093/bjs/znae275

**Published:** 2024-10-30

This is a correction to: This is a correction to: National Institute for Health and Care Research (NIHR) Global Health Research Unit on Global Surgery, Global access to technologies to support safe and effective inguinal hernia surgery: prospective, international cohort study, *British Journal of Surgery*, Volume 111, Issue 7, July 2024, znae164, https://doi.org/10.1093/bjs/znae164.

In the originally published version of this manuscript, the names of the members of the National Institute for Health and Care Research (NIHR) Global Health Research Unit on Global Surgery were not listed in the main manuscript. A ‘**Collaborators**’ section has been added to the manuscript, in which those names now appear.

