# Supplementary material for: Global access to technologies to support safe and effective inguinal hernia surgery: prospective, international cohort study
Source: Br J Surg. 2024 Jul 10;111(7):znae164. doi: 10.1093/bjs/znae164 (PMC11235323; doi:10.1093/bjs/znae164)

**Supplement**

**Global access to technologies to support safe and effective inguinal hernia surgery: A prospective, international cohort study**

NIHR Global Research Health Unit on Global Surgery

A full list of collaborating authors is shown in Appendix 1

**Corresponding authors:**

Dr Maria Picciochi, NIHR Global Health Research Unit on Global Surgery, Institute of Applied Health Research, University of Birmingham, Birmingham B15 2TH, UK

[m.picciochi@bham.ac.uk](mailto:m.picciochi@bham.ac.uk)

**Acknowledgments:** This study was supported by NIHR Global Health Research Unit Grant (NIHR133364) and a project research grant from Portuguese Hernia and Abdominal Wall Society (Sociedade Portuguesa de Hernia e Parede Abdominal). The funders had no role in study design or writing of this report. The views expressed are those of the authors and not necessarily those of the National Health Service, the NIHR or the UK Department of Health and Social Care.

**Appendices**

| Appendix 1 – List of authors | Pages 1-34 |
| --- | --- |
| Appendix 2 – Supplementary tables and materials | Pages 35-41 |

**Appendix 1: List of authors**

**Dissemination Committee** (listed by country)**:**

Albania: I Dajti; Algeria: Z Djama; Argentina: M Lucchini, RM Palacios Huatuco; Australia: K Atherton, AC Dawson, E Lun; Austria: F Aigner; Belgium: F Berrevoet; Benin: I Lawani, S Lawani, C Bokossa; Bosnia and Herzegovina: S Delibegovic; Bulgaria: M Slavchev; Burkina Faso: AF Sanon, A Sanou; Burundi: JB Gusa, JC Mbonicura; Cameroon: A Bang, O Gabom, C Nwegbu; Canada: A Brar, J Martin; Chile: MM Modolo, M Olivos; Colombia: JA Calvache; Croatia: J Mihanovic; Cyprus: N Gouvas, A Yiallourou; Czech Republic: B East; Dominican Republic: S Batista, R Rivas; Ecuador: EP Lincango; Egypt: S Emile; Ethiopia: AB Aregawi; France: AP Arnaud; Gabon: N Boumas; Georgia: Z Demetrashvili; Germany: H Lederhuber, MW Löffler; Ghana: AE Agbeko, NB Sam, S Tabiri, F Agyei, FE Gyamfi, S Mohammed; Greece: I Katsaros, G Tsoulfas; India: L Bains, J Dhiman, D Ghosh, P Haque, A Suroy; Ireland: S Ramjit; Israel: G Marom; Italy: F Pata, G Gallo; Jordan: F Ayasra; Kazakhstan: I R Fakhradiyev; Kenya: IHS Hamdun; Kyrguzstan: A Iqbal; Liberia: E Mbanzabugabo; Libya: M Elhadi; Lithuania: A Gulla; Madagascar: L Samison; Malawi: M Nyirenda, R Nyirenda; Malaysia: AC Roslani; Mali: B Bengaly; Malta: J Psaila; Mexico: L Martinez, A Ramos-De la Medina; Namibia: PR Nashidengo; New Zealand: M McGuinnes, D Wright; Niger: A Ousseini; Nigeria: A Adisa, AO Ademuyiwa; North Macedonia: T Risteski; Oman: Z Al Balushi, B Dawud, A AlSharqi, F Ali; Pakistan: AU Qureshi; Palestine: H Abu-Arish; Paraguay: H Gomez-Fernandez; Philippines: JM Faylona, MD Sacdalan; Poland: W Krawczyk; Portugal: JG Goncalves-Nobre, M Sampaio-Alves, I Santos; Romania: I Negoi; Russian Federation: A Butyrskii; Rwanda: JC Allen, F Ntirenganya; Sierra Leone: I Fortune; Slovenia: J Kosir; South Africa: N Parker, K Chu; Spain: A Minaya Bravo; Sri Lanka: D Wickramasinghe, U Jayarajah; Sudan: M Elmujtaba; Sweden: M Nikberg; Switzerland: E Gialamas; Syria: M Alshaar; Tanzania: M Nkoronko; Turkey: A Isik; Uganda: I Mubesi; United States: J Ng-Kamstra; Venezuela: O Bahsas-Zaky.

**Writing group (Study Management Group):** M Picciochi, AO Ademuyiwa, A Adisa, AE Agbeko, JA Calvache, D Chaudhry, R Crawford, AC Dawson, M Elhadi, A Ghaffar, D Ghosh, J Glasbey, P Haque, E Harrison, A Isik, I Jakaityte, SK Kamarajah, O Kouli, I Lawani, S Lawani, V Ledda, E Li, J Martin, A Minaya Bravo, D Morton, D Nepogodiev, F Ntirenganya, O Omar, SZY Ooi, R Oppong, F Pata, A Ramos-De la Medina, M Sampaio-Alves, JFF Simoes, M Steinruecke, S Tabiri, A Bhangu.

**Data handling and management:** M Picciochi, R Acharya, D Badran, A Chaudhry, JG Goncalves-Nobre, RR Gujjuri, B Kadir (senior statistician), SR Knight, S Lawday, O Omar (senior statistician), KSY Ooi, R Ooi, C Varghese.

**Hospital leads** (listed by country and city)**:**

Albania: E Agastra (Korca; Regional Hospital of Korca); I Dajti (Tirana; University hospital Koco Gliozheni).

Algeria: R Belouz (Algiers; CHU Isaad Hassani); Z Djama (Constantine; University Hospital Abdelhamid Ben Badis); A Mouffokes (Oran; EHU-1st November 1954).

Argentina**:** ME Muriel (Allende, Cordoba; Sanatorio Allende - Sede Cerro); RM Palacios Huatuco (Buenos Aires; Hospital Italiano de Buenos Aires); M Santillan (Buenos Aires; Hospital Universitario CEMIC); A Duro (Buenos Aires; Hospital municipal de vicente lópez); JI Valenzuela (City of Buenos Aires; Hospital Velez Sarsfield); DA Pantoja Pachajoa (Cordoba; Clinica Universitaria Reina Fabiola); G Romero reyna (Cordoba; Sanatorio Allende - Sede Nueva Cordoba); CM Florián Villa (San Francisco; Clinica Regional del Este).

Aruba**:** M Gosselink (Oranjestad; Dr. Horacio E Oduber Hospital).

Australia: YH Lam, A Nguyen (Adelaide; Northern Adelaide Local Health Network); JA Duffield (Adelaide; Royal Adelaide Hospital); A Frankel (Brisbane; Princess Alexandra Hospital); S Bowman (Brisbane; Queen Elizabeth 2 Jubilee Hospital); D Mitchell (Brisbane; Surgical Treatment and Rehabilitation Service); H Iswariah (Brisbane; The Prince Charles Hospital (TPCH)); S Abeykoon (Campbelltown; Campbelltown Hospital); S Gananadha (Canberra; Calvary Hospital); S Gananadha (Canberra; Canberra Hospital); S Salindera (Coffs Harbour NSW; Coffs Harbour Health Campus); S Stevens (Colac; Colac Area Health); AC Dawson (Gosford; Gosford Hospital); M Issa (Hamilton; Hamilton Base Hospital); E Wong (Melbourne; Angliss Hospital); L Bromley (Melbourne; Austin Hospital); E Wong (Melbourne; Box Hill Hospital); K Jaffry (Melbourne; Casey Hospital); M Bickford (Melbourne; Knox Private Hospital); E Wong (Melbourne; Maroondah Hospital); R Nataraja (Melbourne; Monash Childrens Hospital); R Hodgson (Melbourne; Northern Hospital); A Fox (Melbourne; St Vincent’s Hospital); M Wichmann (Mount Gambier; Mount Gambier and Districts Health Service); A Davis (Mount Nasura; Armadale Health Service); S Zhang (Newcastle; Calvary Mater Newcastle); M Ishak, F Amico (Newcastle; John Hunter Hospital); R Liang (Robina; Robina Hospital); P Tang (Sale; Sale Hospital / Central Gippsland Health Service); R Liang (Southport; Gold Coast University Hospital); M Park (Sydney; Royal North Shore Hospital); C Cornwell (Sydney; The Royal Prince Alfred Hospital); E Page-Taylor (Toowoomba; Toowoomba Hospital); A Hameed (Westmead; Westmead Hospital); R McGee (Wyong; Wyong Public Hospital).

Austria: I Königsrainer (Feldkirch; Landeskrankenhaus Feldkirch); F Aigner (Graz; Barmherzige Brüder Krankenhaus, Graz); S Mikalauskas (Graz; Medical University of Graz); L Havranek (Linz; Ordensklinikum Linz Elisabethinen); A Binder (Tulln; Universitätsklinikum Tulln).

Bangladesh: S Islam (Dhaka; Dhaka Medical College Hospital); MP Singh (Dinajpur; Lamb Hospital).

Benin**:** G Gbessi (Cotonou; Centre National Hospitalier et Universitaire Hubert Koutoukou Maga); H Aouagbe Behanzin (Cotonou; Hopital de Menontin); M Agbadebo (Dassa-Zoumè; Hôpital de Zone de Dassa-Zoumè); E Bara (Kandi; Hôpital de zone de Kandi); AB Yevide (Klouékanme; Hopital de Zone de Klouékanme); TK Hessou (Natitingou; Centre Hospitalier Départemental de l’Atacora); AM Hodonou (Parakou; Centre Hospitalier Universitaire Borgou Alibori); I Lawani (Porto Novo; Centre Hospitalier Universitaire et Departemental Oueme Plateau).

Bosnia and Herzegovina: Z Matkovic (Doboj; Genera Hospital ‘Sveti aposto Luka’ Doboj); N Lalovic (Foča; University Hospital Foča); J Miskovic (Mostar; SKB University Clinical Hospital Mostar); M Salibašić (Sarajevo; Clinical Center University of Sarajevo); A Cerovac (Tešanj; General Hospital Tešanj); A Tursunovic (Tuzla; University Clinical Center Tuzla).

Brazil: C Panis (Francisco Beltrão; Universidade Estadual do Oeste do Paraná).

Bulgaria: T Ivanov (Pleven; Heart and Brain - Pleven Hospital); M Karamanliev (Pleven; University Hospital Dr Georgi Stranski, Medical University - Pleven); R Donchev (Plovdiv; MHAT St. Karidad); D Hadzhiev (Plovdiv; UMHAT Sveti Georgi); T Yotsov (Ruse; University Hospital Medika); E Hristova (Sofia; Fifth City Hospital Sofia - 5th MBAL).

Burkina Faso**:** AF Sanon (Ouagadougou; Tengandogo University Hospital).

Burundi**:** JC Mbonicura (Bujumbura; Centre Hospitalo-Universitaire de Kamenge); N Diomede (Bujumbura; Kamenge Military Hospital); J Gusa (Bujumbura; Prince Regent Charles Hospital).

Cambodia: S Stock (Battambang; Handa Medical Centre).

Cameroon: O Ndizeye (Bamenda; Nkwen Baptist Hospital).

Canada**:** R Spence (Halifax; Queen Elizabeth II); S Lee (Port Moody; Eagle Ridge Hospital); S Lee (Vancouver; Royal Columbian Hospital).

Chile: MM Modolo (Santiago; Hospital Barros Luco Trudeau).

China: W Yang (Guangzhou; The First Affiliated Hospital of Jinan University).

Colombia: CJ Perez Rivera (Bogota; Fundacion Cardioinfantil-IC); S Sierra (Medellin; Clínica CES); DS Garcés Palacios (Popayan; Hospital Susana Lopez de Valencia); JA Calvache (Popayán; Hospital Universitario San José).

Croatia: J Mihanovic (Zadar; Zadar General Hospital); G Augustin (Zagreb; University Hospital Centre Zagreb).

Cyprus**:** A Yiallourou (Nicosia; Nicosia General Hospital).

Czech Republic: J Moravik (Decin; Krajská zdravotní as - Hospital Decin); A Al Kaddah (Hradec Kralove; Charles University Hospital); Z Musilová (Ivančice; Hospital Ivančice); M Schön (Novy Jicin; Hospital & Oncological Centre Novy Jicin); J Roman (Ostrava; University Hospital Ostrava); B East (Prague; Motol University Hospital).

Dominican Republic: R Rivas (Santo Domingo; CEDIMAT - Centro de Diagnóstico, Medicina Avanzada, Laboratorio y Telemedicina).

Egypt: G Abouelnagah (Alexandria; Alexandria Main University Hospital); D Attia (Alexandria; Alexandria Medical Research Institute); G Abouelnagah (Alexandria; Smouha University Hospital); A Maher (Assiut; Assiut University Children Hospital); A Kedwany (Assiut; Assiut University Hospital); S Abdelmohsen (Aswan; Aswan University Hospital, Aswan University); R Adel Diab (Cairo; Al Zahraa University Hospital); A Al-Mallah (Cairo; Al-Azhar University Hospitals); H Taher (Cairo; Cairo University Children’s Hospitals (CUSPH & CUCH)); H Abozied (Cairo; EL-Hussein University Hospital, Al-Azhar University, Faculty Of Medicine); ASM Abdelrahman (Cairo; Giza International Hospital); M ElFiky, N Shehata (Cairo; Kasr Al Ainy Faculty of Medicine, Cairo University); B Fahmy (Giza; The Memorial Soaad Kafafi University Hospital); H Elghadban (Mansoura; Mansoura University Hospital); E Adel Mahmod Sultan (Menofia; Menofia University Hospital); M Omar (Qena; Qena University Hospital); EA Ahmed (Sohag; Sohag University Hospital); AM Elkhouly (Tanta; Tanta University Hospital); A Asla (Zagazig; Al Ahrar Zagazig Teaching Hospital).

Ethiopia: F Terefe (Addis Ababa; Yekatit 12 hospital medical college); A Yeshitila (Deberebirhan; Hakim Gizaw Hospital); M Taeme (Dessie; Dessie Referral Hospital); A Demessie (Gondar; Gondar University Comprehensive specialized hospital); AB Aregawi (Hawassa; Hawassa University Comprehensive Specialized Hospital); N S.Bayleyegn (Jimma; Jimma University Medical Center); B Sime (Yirgalem; Yirgalem Hospital Medical College).

France: A Police (Eaubonne; Hôpital Simone Veil); A Castaldi (Nimes; Hôpital Carèmeau); E Reitano (Strasbourg; Nouvel Hopital Civil de Strasbourg).

Gabon: N Boumas (Libreville; Centre Hospitalier universitaire mère enfant Fondation Jeanne Ebori).

Georgia: Z Demetrashvili (Tbilisi; N.Kipshidze Central University Clinic).

Germany: C Kamphues (Berlin; Park-Klinik Weissensee); D Hackner (Erlangen; Universitätsklinikum Erlangen); U Ronellenfitsch (Halle; University Hospital Halle); J Rolinger (Moenchengladbach; Kliniken Maria Hilf); D Reim (Munich; Klinikum Rechts der Isar TUM School of Medicine); N Börner (Munich; Ludwig Maximilian University of Munich - Großhadern); J Goedeke (Munich; Ludwig Maximilian University of Munich - Innenstadt); AE Gut (München; Isarklinikum); M Janda (Rostock; University Hospital Rostock); J De Deken (Saarbruecken; Klinikum Saarbruecken); MW Löffler (Tuebingen; University Hospital Tuebingen).

Ghana: R Armah (Accra; Greater Accra Regional Hospital); A Bediako Bowan (Accra; Korle-Bu Teaching Hospital); E Kafui Ayodeji (Accra; Pentecost Hospital); EA Arkoh (Ankaful; Ankaful Leprosy General Hospital); FE Gyamfi (Berekum; Berekum Holy Family Hospital); A Davor (Bolgatanga; Upper East Regional Hospital); MT Morna (Cape-Coast; Cape Coast Teaching Hospital); N Agboadoh (Damongo; St. Anne’s Hospital); EA Nachelleh (Ho; Ho Teaching Hospital); AE Agbeko (Kumasi; Komfo-Anokye Teaching Hospital); S Mensah (Kumasi; University Hospital, KNUST); P Taah-Amoako (Nsawkaw; Tain District Hospital); K Collins (Sunyani; Brong-Ahafo Regional Hospital); AS Seidu (Tamale; Tamale Teaching Hospital); BK Seshie (Tema; Tema General Hospital); G Ansong (Walewale; Walewale Government Hospital).

Greece: K Kambouri (Alexandroupolis; Alexandroupolis University General Hospital); A Kyriakidis (Amfissa; General Hospital of Amfissa); D Korkolis (Athens; Agios Savvas Anticancer Hospital); N Memos (Athens; Aretaieion Hospital); D Kelgiorgi (Athens; Athens Euroclinic); C Chouliaras (Athens; Athens Medical Center); E Fradelos (Athens; Athens Naval and Veterans Hospital); N Michalopoulos (Athens; Attikon University General Hospital); N Dimitrokallis (Athens; Evaggelismos General Hospital); A Paspala (Athens; Evgenideio Hospital); P Christodoulou (Athens; General Hospital Asklepieio Voulas); EC Tampaki (Athens; KAT Athens General Hospital); D Schizas (Athens; Laiko University Hospital); K Kontzoglou (Athens; National and Kapodistrian University of Athens); M Spartalis (Athens; Sotiria General Hospital of Thoracic Diseases); M Billis (Filiates; General Hospital of Filiates); N Tsakiridis (Florina; Florina General Hospital ‘Eleni Th. Dimitriou’); A Karakosta (Ioannina; University Hospital of Ioannina); D Panagopoulos (Kyparissia; General Hospital of Messinia, Hospital Unit of Kyparissia); G Koukoulis (Larissa; General Hospital of Larissa ‘Koutlimpaneio and Triantafylleio’); G Christodoulidis (Larrisa; General University Hospital of Larissa); K Bouchagier (Patras; General University Hospital of Patras); V Mousafeiris (Patras; Karamandaneio Prefecture Children Hospital of Patras); A Papadopoulos (Piraeus; General Hospital of Nikaia); L Katsiaras (Piraeus; Metaxa Cancer Hospital); N Zampitis (Piraeus; Tzaneio General Hospital); O Ioannidis (Thessaloniki; George Papanikolaou General Hospital of Thessaloniki); M Drogouti (Thessaloniki; O Agios Dimitrios General Hospital); C Kaselas (Thessaloniki; Papageorgiou General Hospital); D Lytras (Volos; Achillopoyleio General Hospital of Volos).

Guatemala: M Aguilera-Arevalo (Guatemala City; Hospital General San Juan De Dios); L Talé-Rosales (Guatemala City; Hospital Juan Jose Arevalo Bermejo); ST Torres Rodríguez (Guatemala City; Hospital de Referencia Nacional de Enfermedades Respiratorias).

India: N Krishnappa (Bangalore, Karnataka; BGS Global Institute of Medical Sciences); S Kumar Venkatappa (Bangalore; Victoria Hospital); TS Mishra (Bhubaneswar; All India Institute Of Medical Sciences - Bhubaneswar); Y Sakaray (Chandigarh; Postgraduate Institute of Medical Education & Research, Chandigarh, India); R Kottayasamy Seenivasagam (Coimbatore; PSG Institute of Medical Sciences and Research); R Sharma (DELHI; St Stephen’s Hospital); R Gupta (Dehradun; Synergy Institute of Medical Sciences); M Luthra (Delhi; Holy Family Hospital); T Longkumer (Dimapur; Christian Institute of Health Sciences and Research); A Chhabra (Faridkot; Guru Gobind Singh Medical College & Hospital (Baba Farid University of Health Sciences)); T Doma Bhutia (Gangtok; Sir Thutob Namgyal Memorial Hospital Sochakgang); M Pathak (Jodhpur; All India Institute of Medical Sciences (AIIMS), Jodhpur); J Rathod (Karamsad; Shree Krishna Hospital); MK Agrawal (Lucknow; King George’s Medical University); D Jain (Ludhiana; Christian Medical College & Hospital); NK Chaudhry (Ludhiana; Satguru Partap Singh Hospital); A Mathew (Madhepura; Madhepura Christian Hospital); P Alexander (Manali; Lady Willingdon Hospital); V Kumar (Manipal; Kasturba Medical College Hospital, Manipal); RD Sharma (Mumbai; Lilavati Hospital & Research Centre); B Sarang (Mumbai; Terna Medical College and Hospital); D Singh (Nandurbar; Chinchpada Christian Hospital); N Gupta (New Delhi; ABVIMS Dr RML Hospital); S Kulkarni (New Delhi; Army Hospital Research & Referral New Delhi); T Rashid (New Delhi; Hamdard Institute of Medical Sciences & Research); L Bains (New Delhi; Maulana Azad Medical College); T Iahmo (Padhar; Padhar Hospital); A Kumar (Patiala; Government Medical College Patiala); M Kumar (Patna; All India Institute of Medical Sciences, Patna); R Abhinaya (Pondicherry; Jawaharlal Institute of Postgraduate Medical Education and Research); VS Jha (Pune; Command Hospital, Southern Command); D Dugar (Raipur; All India Institute of Medical Sciences Raipur); S Basu (Rishikesh; All India Institute Of Medical Sciences); K Singh (SAS Nagar (Mohali) ; BR Ambedkar State Institute of Medical Sciences Mohali); C Mahakalkar (Sawangi (Meghe), Wardha; Acharya Vinoba Bhave Rural Hospital); FQ Parray (Srinagar; Sher-i-Kashmir Institute of Medical Sciences); JA Kalyanapu (Tezpur; Baptist Christian Hospital); M Chisthi (Thiruvananthapuram; Government Medical College Thiruvananthapuram); A Kavalakat (Thrissur; Jubilee Mission Medical College & Research Institute); B Roopavathana. S (Vellore; Christian Medical College & Hospital).

Iran, Islamic Rep.: N Yousefzadeh Kandevani (Bastak; Farabi hospital); M Pourfridoni (Jiroft; Imam Khomeini Hospital).

Iraq: R Raheem Attallah Al_obaidy (Anbar; Heet General hospital); Z Alkhuzaie (Najaf; Al Batool private hospital); MA Al-Juaifari (Najaf; Al-Najaf Al-Ashraf Teaching Hospital).

Ireland: S Ramjit (Dublin; St James’s Hospital); R Tummon (Kerry; University Hospital Kerry).

Israel: S Abu Salem (Jerusalem; Hadassah Medical Center).

Italy: R Sulce (Arezzo; Ospedale San Donato USL Toscana Sud Est); PM Cicerchia (Ariccia; Ospedale dei Castelli (N.O.C.)); E Marra (Aversa; San Giuseppe Moscati); M Rottoli (Bologna; IRCCS Azienda Ospedaliero-Universitaria di Bologna); J Andreuccetti (Brescia; ASST Spedali Civili, Ospedale di Brescia); E Locci (Cagliari; Cagliari University Hospital); F Cappellacci (Cagliari; Chirurgia Generale e Polispecialistica, Cagliari University Ospital ‘Duilio Casula’); N Cillara (Cagliari; Santissima Trinità - ATS Sardegna); E Abate (Carate Brianza (MB); Ospedale Vittorio Emanuele III - Carate Brianza); F Ascari (Carpi; Ramazzini); S Romano (Casarano; Francesco Ferrari Hospital); M Veroux (Catania; Azienda Ospedaliero- Universitaria Policlinico San Marco); B Nardo (Cosenza; Azienda Ospedaliera di Cosenza); D Sasia (Cuneo; Santa Croce e Carle Hospital, Cuneo); G Baronio (Esine; ASST Valcamonica Ospedale di Esine); N Fabbri (Ferrara; Azienda Unità Sanitaria Locale di Ferrara); J Martellucci (Firenze; Azienda Ospedaliera Universitaria Careggi); G Canonico (Firenze; Ospedale San Giovanni di Dio); V Lizzi (Foggia; Ospedali Riuniti Azienda Ospedaliera Universitaria); F D’acapito (Forlì; Morgagni-Pierantoni); D Merlini (Garbagnate Milanese; ASST Rhodense - Ospedale di Garbagnate Milanese); A Barberis (Genoa; E.O. Ospedali Galliera); MF Amisano (Genoa; IRCCS Ospedale Policlinico San Martino); A Luzzi (Genoa; Ospedale Villa Scassi); F Palmieri (Gravedona ed Uniti; Ospedale Moriggia Pelascini); CL Bertoglio (Magenta; Ospedale ‘G.Fornaroli’, ASST-OVEST Milanese); E Baldini (Melzo; Ospedale Santa Maria delle Stelle, ASST Melegnano Martesana); M Ceolin (Milan; Humanitas Research Hospital); P De Nardi (Milan; IRCCS San Raffaele Scientific Institute, Milan); MG Piacentini (Milan; Ospedale Fatebenefratelli e Oftalmico); F Ferrara (Milan; San Carlo Borromeo); F Brucchi (Milan; Sesto San Giovanni Hospital); F Di Marco (Modica; Ospedale Maggiore); N Tamini (Monza; Fondazione IRCCS San Gerardo dei Tintori Monza, Scuola di Medicina e Chirurgia, Università Milano Bicocca); P Anoldo (Naples; Federico II University of Naples); R Patrone (Naples; Istituto Nazionale Tumori Fondazione, Pascale IRCCS); F Selvaggi (Naples; Primo Policlinico di Napoli); G Bellio (Padova; Piove di Sacco Hospital); P Venturelli (Palermo; Policlinico Universitario Paolo Giaccone); L Conti (Piacenza; G. Da Saliceto); L Morelli (Pisa; Azienda Ospedaliero Universitaria Pisana); SMM Basso (Pordenone; Azienda Sanitaria Friuli Occidentale (AS FO)); F Biolchini (Reggio Emilia; Azienda Unità Sanitaria Locale - IRCCS di Reggio Emilia); C Marafante (Rivoli; Ospedale degli Infermi di Rivoli); A Antinori (Rome; Fondazione Policlinico Universitario Agostino Gemelli); M Campanelli (Rome; Policlinico Tor Vergata Hospital, Rome); P Lapolla (Rome; Policlinico Umberto I Sapienza University of Rome); G Palomba (Salerno; San Giovanni di Dio e Ruggi d’Aragona); L Cardinali (San Benedetto del Tronto; Madonna del Soccorso Hospital); E Andolfi (Sansepolcro; Valtiberina); L Verre (Siena; Azienda Ospedaliero Universitaria Senese); G Poillucci (Spoleto (PG); San Matteo degli Infermi); E Pontecorvi (Sulmona; SS Annunziata); S Novello (Treviso; Ospedale Ca’ Foncello - Università di Padova (DISCOG)); M Santarelli (Turin; Città della Salute e della Scienza); L Cobellis (Vallo Della Lucania; Casa di Cura Prof. Dott. Luigi Cobellis); G Ietto (Varese Lombardy; University of Insubria, Ospedale di Circolo e Fondazione Macchi (Varese)); F Pederiva (Varese; Filippo Del Ponte Hospital, University of Insubria); A Iacomino (Venezia; Ospedale Civile - Santi Giovanni e Paolo); D Verdi (Venice; Mirano Hospital); A Broglia (Voghera; Ospedale Civile di Voghera); P Cianci (andria; Lorenzo Bonomo); M Angelucci (rome; policlinico universitario campus bio medico of rome); G Calini (udine; santa maria della misericordia).

Japan: H Yonekura (Aichi; Fujita Health University Bantane Hospital).

Jordan: S Alananzeh (Ajloun; Al Iman Hospital); A Qasem (Amman; Al-Basheer Hospital); Y Alawneh (Amman; Ibn Al Haitham Hospital); S Al-Tahayneh (Amman; Islamic Hospital); A Khamees (Amman; Jordan University Hospital); MEH Albanna (Amman; Marka Specialty Hospital); RKZ Almahadin (Amman; Prince Hamza hospital); M Mahafdah (Ar Ramtha; King Abdullah University Hospital/ Jordan University of Science and Technology); O Mansour (As-Salt; Al Hussain New Salt Hospital); M Mubarak (Irbid; Ar Ramtha Govermental Hospital); A Alrababah (Irbid; Princess Basma Hospital).

Kazakhstan: M Kulimbet (Almaty; City Clinical Hospital No.7, Asfendiyarov Kazakh National Medical University); I Fakhradiyev (Almaty; JSC ‘Central Clinical Hospital’, Asfendiyarov Kazakh National Medical University).

Kenya: R Parker (Bomet; Tenwek Hospital).

Lebanon: D Rahme (Beirut; Hopital Libanais Geitaoui); H Hamdar (Jbail ; Maritime Hospital).

Libya: W Ebrahim (Albayda; Albayda Medical Center); M Saleh (Benghazi; Al-jalaa Teaching/Trauma Hospital); S Alsaeiti (Benghazi; Benghazi Children’s Hospital); R Michael (Benghazi; Benghazi Medical Center); A Bojazyah (Darna; Al-Wahda Hospital); H Bileid Bakeer (Gharyan; Gharyan Central Hospital); M Abudabbous, N Albahloul (Misurata; Misurata Central Hospital); A Egdeer (Nalut; Nalut Central Hospital); H Embarek (Sebha; Al-Majd Clinic); M Abdelkabir (Sebha; Aseel Alghad Clinic); H Idheiraj (Sebha; Sabha Medical Center); R Salim (Tobruk; Tobruk Medical Center); K Ayad (Tripoli; Alkhalil hospital); A Alragheai (Tripoli; Metiga Hospital); S Egreara (Tripoli; Sabratha teaching hospital); S Timmalah (Tripoli; Tripoli Medical Center/ Tripoli University Hospital); N Lahmer (Zawia; Zawia Teaching Hospital); N Ben Hasan (Zliten; Zliten Teaching Hospital).

Lithuania: D Venskutonis (Kaunas; LUHS Kaunas Hospital); A Dauksa (Kaunas; Lithuanian University of Health Sciences Kaunas Clinics); A Gulla (Vilnius; Vilnius University Hospital).

Madagascar: F Rasoaherinomenjanahary (Antananarivo; Joseph Ravoahangy Andrianavalona Hospital).

Malaysia: MS Mohd Shah (Kelantan; Hospital Universiti Sains Malaysia); R Noor (Kota Bharu; Hospital Raja Perempuan Zainab II); SN Loke (Kuching, Sarawak; Sarawak General Hospital); MA Yunus (Malacca; Hospital Jasin); H Amin-Tai (Serdang; Hospital Pengajar Universiti Putra Malaysia (HPUPM)).

Mali: KS Dembele (Ségou; District Hospital of Tominian).

Malta: J Psaila (Msida; Mater Dei Hospital).

Mexico: CM Nuño-Guzmán (Guadalajara; Antiguo Hospital Civil de Guadalajara); LA Flores Chávez (Guadalajara; Clínica de Especialidades más Centro de Cirugía Simplificada); CM Nuño-Guzmán, G Yanowsky-Reyes (Guadalajara; Hospital Civil Fray Antonio Alcalde); A Gonzalez Ojeda (Guadalajara; Hospital de Especialidades, CMNO-IMSS); G Ambriz González (Guadalajara; UMAE Hospital de Pediatria Centro Medico Nacional de Occidentes IMSS); EE Lozada Hernandez (León; Hospital Regional e Alta Especialidad del Bajio); M Trejo-Avila (Mexico City; Hospital General Dr. Manuel Gea González); C Moreno-Licea (Mexico City; Instituto Nacional de Ciencias Médicas y Nutrición ‘Salvador Zubirán’); A Navarrete-Peón (Pachuca; Sociedad Española de Beneficencia); M Noguez Castillo (Querétaro; Hospital de especialidades del niño y la mujer); A Ramos-De la Medina (Veracruz; Hospital Español Veracruz).

Morocco: I Gouazar (Marrakech; Centre Hospitalier Universitaire Mohammed VI, Marrakech); A Ouachhou (Rabat; Centre Hospitalier Universitaire Ibn Sina Rabat).

Namibia: PR Nashidengo (Windhoek; Windhoek Central Academic Hospital).

New Zealand: S Rennie (Masterton; Wairarapa Hospital); M Haimona (Wellington; Wellington Regional Hospital); F Fadzlullah (Whanganui; Whanganui Hospital); L Paterson (Whangarei; Whangarei Hospital).

Nigeria: A Adeyeye (Ado Ekiti; Afe Babalola University Multi-System Hospital); J Olaogun (Ado-Ekiti; Ekiti State University Teaching Hospital); N Oloko (Bauchi; Abubakar Tafawa Balewa University Teaching Hospital Bauchi); P Agbonrofo (Benin City; University of Benin Teaching Hospital); A Abiodun (Bida; Federal Medical Centre Bida); U Ezomike (Enugu; University of Nigeria Teaching Hospital); SA Sani (Gwagwalada; University of Abuja Teaching Hospital); TA Lawal (Ibadan; University College Hospital); AO Lawal (Idi Araba; Lagos University Teaching Hospital); AI Okunlola (Ido Ekiti; Federal Teaching Hospital, Ido Ekiti); OM Williams (Ikeja; Lagos State University Teaching Hospital); A Adisa (Ile-Ife; Obafemi Awolowo University Teaching Hospitals Complex); T Mohammed (Ilesa; Obafemi Awolowo University Teaching Hospitals Complex Wesley Guild Hospital Unit); P Elemile (Ilishan-Remo; Babcock University Teaching Hospital); II Aremu (Ilorin; General Hospital); L Abdur-Rahman (Ilorin; University of Ilorin Teaching Hospital); JG Makama (Kaduna; Barau Dikko Teaching Hospital); IU Garzali (Kano; Aminu Kano Teaching Hospital); TT Ibiyeye (Lokoja; Federal Teaching Hospital Lokoja); OH Ekwunife (Nnewi; Nnamdi Azikiwe University Teaching Hospital); OH Ekwunife (Onitsha; Holy Rosary Specialist Hospital); O Ojewuyi (Osogbo; UNIOSUN Teaching Hospital); I Ogundele (Sagamu; Olabisi Onabanjo University Teaching Hospital); M Daniyan (Zaria; Ahmadu Bello University Teaching Hospital).

North Macedonia: T Risteski (Skopje; University Clinic for Pediatric Surgery).

Oman: H Al-Aamri (Ibra; Ibra Hospital); AH ALSharqi (Muscat; Sultan Qaboos University Hospital); M Al Hinai (Nizwa; Nizwa Hospital).

Pakistan: S Ahmed (Islamabad; Dr Akbar Niazi Teaching Hospital); SH Waqar (Islamabad; The Pakistan Institute of Medical Sciences); M Shahid (Karachi; PAF Faisal Hospital); F Ashraf (Karachi; Patel Hospital); AN Syed (Karachi; The Indus Hospital); AS Ammar (Lahore; Bahria International Hospital, Bahria Orchard); K Hayat (Lahore; Services Hospital Lahore); N Talat (Lahore; The Children’s Hospital & The Institute of Child Health Lahore); W Mabood (Peshawar; Mercy Teaching Hospital); HW Bhatti (Rawalpindi; Benazir Bhutto Hospital); M Usman Malik (Sargodha; District Headquarter & Teaching Hospital - Sargodha).

Palestine: B Mohamad (Bethlehem, West Bank; Beit Jala Governmental Hospital (Al Hussein)); A Alwali (Gaza; Al-Shifa Hospital); A AbuNemer (Gaza; Nasser Hospital); S Alijla (Gaza; Palestine Red Crescent Society - Al-amal Hospital); H Ayesh (Hebron, West Bank; Al-Ahli Hospital); H Abu-Arish (Hebron, West Bank; Governmental Hebron Hospital-Alia); D Rabaia, A Jaber (Jenin, West Bank; The Martyr Dr. Khalil Sulaiman Hospital (Jenin Governmental Hospital)); M MohammedAli (Nablus, West Bank; Rafidia Hospital); A Attili (Tulkarm, West Bank; Martyr Thabet Thabet Govermental Hospital).

Paraguay: OM Cuenca Torres (Asuncion; Hospital de Clínicas, II Cátedra de Clínica Quirúrgica, Universidad Nacional de Asunción).

Peru: V Panduro-Correa (Huánuco; Hospital Regional Hermilio Valdizán Medrano); L Fuentes Rivera Lau (Lima; British American Hospital); CF Huaroto Landeo (Lima; Clinica Internacional); G Mendiola (Lima; Hospital Santa Rosa de Lima); Y Carpio Colmenares (Lima; SANNA - Clínica El Golf); C Shiraishi Zapata (Paita; Hospital I Miguel Cruzado Vera EsSalud); R Díaz-Ruiz (Piura; Jose Cayetano Heredia III Regional Hospital).

Poland: Ł Nawacki (Kielce; Wojewódzki Szpital Zespolony w Kielcach); M Kisielewski (Krakow; 5th Military Clinical Hospital); Z Orzeszko (Krakow; Brothers Hospitallers Hospital); M Matyja (Krakow; Jagiellonian University Medical College); W Krawczyk (Sosnowiec; Wojewódzki Szpiital Specjalistyczny nr 5 im. Św Barbary); M Walędziak (Warsaw; Military Institute Of Medicine); JK Zajac (Wroclaw; Regional Specialist Hospital in Wroclaw); F Brzeszczyński (Łódź; Copernicus Memorial Hospital).

Portugal: S Henriques (Almada; Hospital Garcia de Orta); J Frazão (Amadora; Hospital Prof. Doutor Fernando Fonseca, E.P.E.); S Gaspar Reis (Barreiro; Centro Hospitalar Barreiro Montijo, EPE); AR Mateus Loureiro (Caldas da Rainha; Hospital das Caldas da Rainha - Centro Hospitalar do Oeste, E.P.E); M Reia (Elvas; Hospital Santa Luzia Elvas); J Pinho (Figueira da Foz; Hospital Distrital da Figueira da Foz); DG Alves (Funchal; Hospital Dr. Nélio Mendonça); R Silva Borges (Horta; Hospital da Horta, E.P.E.); E Borges (Lisbon; Centro Hospitalar Lisboa Norte); M Nunes (Matosinhos; Unidade Local de Saude de Matosinhos - Hospital Pedro Hispano); A Faustino (Ponta Delgada; Hospital do Divino Espírito Santo); G Fialho (Portalegre; Hospital Doutor José Maria Grande); J Dias-Ferreira (Porto; Centro Hospitalar e Universitário de São João); M Santos (Porto; Hospital da Prelada); J Marques Antunes (Santa Maria da Feira; Centro Hospitalar Entre o Douro e Vouga); H Devesa (Santarem; Hospital de Santarem); J Ricardo (Santiago do Cacém; Hospital do Litoral Alentejano); R Branquinho (Tomar; Centro Hospitalar Médio Tejo); J Fernandes (Vila Franca de Xira; Hospital Vila Franca de Xira); J Pereira-Macedo (Vila Nova de Famalicao; Centro Hospitalar do Medio Ave); B Vieira (Vila Real; Centro Hospitalar de Trás-os-Montes e Alto Douro, E.P.E.).

Qatar: CE Guldogan (Doha; Turkish Hospital).

Romania: ST Makkai-Popa (Brasov; Regina Maria); F Grama (Bucharest; Coltea Clinical Hospital); EA Toma (Bucharest; Elias Emergency Hospital); I Negoi (Bucharest; Emergency Clinical Hospital Bucharest); M Muresan (Cluj-Napoca; Medicover Hospital Cluj).

Russian Federation: V Kakotkin (Kaliningrad; Immanuel Kant Baltic Federal University, Regional Clinical Hospital); A Bedzhanyan (Moscow; Petrovsky National Research Centre of Surgery); S Katorkin (Samara; Hospital Surgery Clinic of Samara State Medical University); A Butyrskii (Simferopol; Municipal Emegency Hospital No.6); V Ten (Yuzhno-Sakhalinsk; Private healthcare institution ‘RZD-Medicine’).

Rwanda: N Christian (Huye, Gisagara; Butare university teaching hospital (CHUB)); C Mpirimbanyi (Kigali; Kibagabaga Hospital); A Costas-Chavarri (Kigali; Rwanda Military Hospital).

Saudi Arabia: N Alzerwi (Al-Majmaah; King Khalid General Hospital); A Shabkah (Jeddah; International Medical Center); A Nawawi (Jeddah; King Abdulaziz University Hospital); N Alzerwi (Riyadh; King Salman Hospital in Riyadh); S Chowdhury (Riyadh; King Saud Medical City); DY Alalawi (Tabuk; King Salman Armed Forces Hospital); S Awad (Taif City; King Faisal Medical Complex).

Serbia: A Karamarkovic (Belgrade; Zvezdara University Medical Center).

Slovenia: JA Košir (Ljubljana; University Medical Centre).

South Africa: M Flint (Cape Town; Groote Schuur Hospital); A Victor (Cape Town; Karl Bremer Hospital); SS Verhage (Cape Town; Khayelitsha District Hospital); F Gool (Cape Town; Mitchell’s Plain District Hospital); T Mabogoane (Cape Town; Victoria Hospital Wynberg); B Phakathi (Durban; King Edward VIII Hospital); V Pillay (Durban; Stanger Hospital); R Jayakrishnan (East London; Cecilia Makiwane Hospital); Y Manickchund (East London; Frere Hospital); O Jolayemi (Empangeni; Ngwelezana Hospital); H Stark (George; George Hospital); C Molewa (Johannesburg; Edenvale); H Wain (Pietermaritzburg; Edendale Hospital); D Montwedi (Pretoria; Kalafong Academic Hospital); G De Wee (Upington; Dr Harry Surtie Hospital); C Dempers (Worcester; Worcester Provincial Hospital).

Spain: H Aguado López (Albacete; Hellín Hospital); MDM Martí-Ejarque (BARCELONA; Hospital Universitari Sagrat Cor); A Torroella (Barcelona; HM Nou Delfos); O Martin Sole (Barcelona; Hospital Sant Joan de Deu); A Landaluce-Olavarria (Bizkaia; Hospital Urduliz); V Alonso (Burgos; Hospital Universitario de Burgos); Á Fernández Camuñas (Ciudad Real; Hospital General Universitario de Ciudad Real); M Estaire Gómez (Leganés; Severo Ochoa University Hospital); AG Barranquero (Lleida; Hospital Universitari Arnau de Vilanova); L Marquez (Madrid; Hospital Central de la Cruz Roja San Jose y Santa Adela); P Serrano Méndez (Madrid; Hospital Clinico San Carlos); A Vilar (Madrid; Hospital Universitario Principe de Asturias); J Guevara (Madrid; Hospital Universitario la Paz); AM Minaya Bravo (Madrid; Hospital del Henares); JL Rodicio Miravalles (Oviedo; Hospital Universitario Central de Asturias (HUCA)); R Díaz Pedrero (Rivas-Vaciamadrid, Madrid; Hospital Universitario HM Rivas); L Tallon-Aguilar (Sevilla; Hospital Universitario Virgen del Rocio); A Curado Soriano (Seville; Hospital Universitario Virgen Macarena); Z Balciscueta (Valencia; Hospital Arnau de Vilanova); D Moro-Valdezate (Valencia; Hospital Clínico Universitario de Valencia); B De Andrés-Asenjo (Valladolid; Hospital Clínico Universitario de Valladolid); A Vazquez Melero (Vitoria-Gasteiz; Hospital Universitario Araba); J Escartin (Zaragoza; Hospital Royo Villanova); C Gracia-Roche (Zaragoza; Hospital Universitario Miguel Servet).

Sri Lanka: S Srishankar (Anuradhapura; Teaching Hospital Anuradhapura); D Wickramasinghe (Colombo; National Hospital of Sri Lanka); U Jayarajah (Dehiwala; Colombo South Teaching Hospital); S Gobishangar (Jaffna; Teaching Hospital, Jaffna); W Wijenayake (Werahera; University Hospital, Kotelawala Defence University).

Sudan: I Abdalla (Ed Dueim; Ed Dueim Teaching Hospital); S Ibrahim Tour Harakan (El Geneina; El Geneina teaching Hospital); MM Yassin (Gadarif city; Gadarif teaching hospital); Z Aljalabi (Kassala; Police hospital); I Adel (Khartoum; Bashair Teaching Hospital); IMG Ahmed (Khartoum; Ibrahim Malik Teaching Hospital); M Hajalamin (Khartoum; Omdurman Teaching Hospital); EE Abuobaida Banaga Hag El Tayeb (Khartoum; Ribat university hospital); HA Fadlalmola (Khartoum; Soba University Hospital); E Alkhalifa (Wad Madani; University of Gezira Hospital).

Sweden: H Zaigham (Malmö; Skåne University Hospital); M Nikberg (Vasteras; Västmanlands Hospital Västerås).

Switzerland: P Probst (Frauenfeld; Spital Thurgau AG); E Gialamas (Geneva; Geneva University Hospitals); J Gass (Luzern; Luzerner Kantonsspital); A Tampakis (Olten; Kantonsspital Olten); G Peros (Winterthur; Kantonsspital Winterthur); MA Schneider (Zurich; University Hospital of Zurich).

Syrian Arab Republic: MA Farho (Aleppo; Abd Al Wahab Agha Hospital); AA Kayali (Aleppo; Al-Shahbaa Private Hospital); M Aloulou (Aleppo; Aleppo Private Hospital); A Ghazal (Aleppo; Aleppo University Hospital); B Alsaid (Damascus; Al assad university hospital); M Klib (Damascus; Al-Mouwasat University Hospital); H Dalati (Damascus; Children’s University Hospital); S Jomaa (Damascus; Damascus Hospital); Y Alhammoud (Homs; Al-Basel Specialized Hospital in Karm El-Louz); S Abbas (Homs; The Military Hospital); G Hneino (Latakia; Al Saydeh Surgical Hospital); I Ali (Latakia; National Hospital); G Bashour (Latakia; Othaman Hospital); A Hammed (Latakia; Tishreen University Hospital).

Thailand: S Techapongsatorn (Bangkok; Vajira hospital).

Togo: F Alassani (Lomé; CHU Sylvanus Olympio).

Tunisia: A Sebai (Tunis; La Rabta Hospital).

Turkey: GC Bulbuloglu (Adana; Adana Seyhan State Hospital); MA Koç (Ankara; Ankara University Medical School); MY Uzunoglu (Bursa; Bursa City Hospital); B Yigit (Elazig; Elazig Fethi Sekin City Hospital); AN Sanli (Gaziantep; Abdulkadir Yuksel State Hospital); GK Kurtoglu (Istanbul; Acibadem Altunizade Hospital); E Tuzuner (Istanbul; Acibadem Maslak Hospital); Y Altinel (Istanbul; Bagcilar Research And Training Hospital); ÖP Zanbak Mutlu (Istanbul; Bahçelievler State Hospital); RE Sönmez (Istanbul; Istanbul Medeniyet University, School of Medicine); S Bektas (Istanbul; Istanbul Medipol University Hospital); E Erginöz (Istanbul; Istanbul universty - Cerrahpaşa Medical faculty); A Özcan (Istanbul; Kanuni Sultan Suleyman Training and Research Hospital); Y Tosun (Istanbul; Kartal Dr. Lutfi Kirdar Training and Research Hospital); İH Özata (Istanbul; Koç University Medical School); TK Uprak (Istanbul; Marmara University, School of Medicine); E Unal (Istanbul; Sehit Prof.Dr. İlhan Varank Training and Research Hospital); N Kiziltoprak (Istanbul; Sultan 2. Abdulhamid Han Training and Research Hospital, University of Health Sciences); M Ergenç (Istanbul; Sultanbeyli State Hospital); MT Demirpolat (Istanbul; University of Health Science Umraniye Education and Research Hospital); B Citgez (Istanbul; Uskudar University Faculty of Medicine, Memorial Hospital); H Ulman (Izmir; Bakircay University Cigli Training and Research Hospital); YK Şen (Izmir; University of Health Sciences Izmir Bozyaka Training and Research Hospital); E Colak (Samsun; Samsun University Samsun Training and Research Hospital); N Kavak (Zonguldak; Zonguldak Bulent Ecevit University School of Medicine Research and Training Hospital); E Kamer (İzmir; University of Health Sciences Tepecik Training and Research Hospital).

Uganda: I Mubezi (Iganga; Iganga district hospital); H Lule (Kigumba; Kiryandongo Hospital); S Stonelake (Luwero; Kiwoko Hospital).

United Kingdom: CS Ong (Bangor, North Wales; Ysbyty Gwynedd); P Patel (Barrow in Furness; Furness General Hospital); S Dindyal (Basildon; Basildon University Hospital); F Georgiades (Bedford; Bedford Hospital); J Abbasy (Birmingham; Heartlands Hospital); M Kaur (Brighton; Royal Sussex County Hospital); B Martin (Bristol; Bristol Royal Hospital for Children); M Chauhan (Camberley; Frimley Health NHS FT - Frimley Park); S Ahmed (Chester; Countess of Chester Hospital); M Tutton (Colchester; Colchester Hospital University); N Chidumije (Coventry; University Hospitals Coventry and Warwickshire NHS Trust); W Al-Khyatt (Derby; Royal Derby Hospital); A Sukumar (Dudley; Russell’s Hall Hospital); H Kamal (Dundee; Ninewells Hospital); A Nada (Durham; University Hospital North Durham); A Chaudhary (Exeter; Royal Devon and Exeter Hospital); M Bogdan (Great Yarmouth; James Paget Univeristy NHS Foundation Trust Hospital); M Peter (Huddersfield; Huddersfield Royal Infirmary); J Walshaw (Hull; Hull University Teaching Hospitals NHS Trust); M Ewedah (Ilford; King George Hospital); L Rampersad (Larbert; Forth Valley Royal Hospital); A Peckham-Cooper, NS Blencowe (Leeds; Leeds Teaching Hospitals); R Lunevicius (Liverpool; Aintree University Hospital); P Panahi (London; Ealing Hospital); E Baili (London; Guy’s and St Thomas’ Hospitals); K Theodoropoulou (London; Homerton University Hospital); P Kapsampelis (London; Kingston); MMH Mohammed (London; Queen Elizabeth Hospital, Woolwich); C Parmar (London; The Whittington Hospital); MMH Mohammed (London; University Hospital Lewisham); C Smart (Macclesfield; Macclesfield District General Hospital); P Wilson (Manchester; Wythenshawe Hospital); F Gareb (Margate; Queen Elizabeth the Queen Mother Hospital Margate); G Sundaram Venkatesan (Middlesbrough; James Cook University Hospital); C Hidalgo Salinas (Morecambe; Royal Lancaster Infirmary); S Tingle (North Shields; Northumbria NHS Hospital Trust); N Marzouqa (Nottingham; Queens Medical Centre); T Theivendrampillai (Oxford; John Radcliffe Hospital); M Zahed Abdalla (Portsmouth; Queen Alexandra Hospital); A Rahman (Redhill; East Surrey Hospital); M Abdelkarim (Rhyl; Glan Clwyd Hospital); O Whitehurst (Salford; Salford Royal Hospital); E Tokidis (Sheffield; Sheffield Teaching Hospital NHS Foundation Trust); S Bandyopadhyay (Southampton; Southampton General Hospital); A Evans (Swansea; Morriston Hospital Swansea); A Salam (Walsall; Walsall Manor Hospital).

United States: M Sulciner (Boston; Brigham and Women’s Hospital); G Chang (Chicago, IL; Mount Sinai Hospital); A Alecci (Chicago, IL; Rush University Medical Centre); HE Rice (Durham, NC; Duke University Medical Center); D Ridder (Honolulu, Hawaii; The Queen’s Medical Center); K McKenzie (Jamaica; Jamaica Hospital); A Choudhry (Syracuse; SUNY Upstate University Hospital).

Yemen, Rep.: MY Abdualqader (Hajjah; Kowaydina hospital); B Alshaikh (Sana’a; Al-Thawra Modern General Hospital).

**Collaborators** (listed by country and city)**:**

Albania: B Ibi, S Faber (Korca; Regional Hospital of Korca); I Dajti (Tirana; University hospital Koco Gliozheni).

Algeria: K Bensmain, ZR Benamrouche , IE Boumakhlouf, IE Boudis, H Abdoun , M Benamrouche , M Saidani (Algiers; CHU Isaad Hassani); Z Djama, A Chied, HA Mimouni (Constantine; university hospital abdelhamid ben badis); AK Awad, B Radja, B Abdennour , MN Bouhafs, MEA Meghaizerou (Oran; EHU-1st November 1954).

Argentina: P Carmignani, J Mondino, R Figueroa, J Morales, FR Pascual (Allende, Cordoba; Sanatorio Allende - Sede Cerro); MA Bequis, F Suldrup, C Korzin, J Napoli , N Feijoo, F Mahnic, ME Duran, L Chantada, C Brandi, JF Viñas, F Lucero, C Samojeden, LJ Caram, S Bertone, F Corvatta (Buenos Aires; Hospital Italiano de Buenos Aires); L Garciandia (Buenos Aires; Hospital Universitario CEMIC); M Rius, S Matthiess, J Paredes, MC Kalaydjian , A Veira (Buenos Aires; Hospital municipal de vicente lópez); MA Fernández Zurita, JI Valenzuela (City of Buenos Aires; Hospital Velez Sarsfield); S Gomez, GR Viscido, MA Doniquian (Cordoba; Clinica Universitaria Reina Fabiola); R Badra, JS García, CI Ferrero, M Garcia, L Granero (Cordoba; Sanatorio Allende - Sede Nueva Cordoba); M Pagani (San Francisco; Clinica Regional del Este).

Aruba: M Gosselink, J Ringers (Oranjestad; Dr. Horacio E Oduber Hospital).

Australia: M Lie, B Mao, C Stennard, EMA Murphy, JE Do, M Harris, B Fosh (Adelaide; Northern Adelaide Local Health Network); M Watson, J Petric, M Maclean, XY Po, M Pham, D Patterson, V Gunasaegaram, E Hopping, P Holt, JA Duffield (Adelaide; Royal Adelaide Hospital); E Schmidt, R Colbran (Brisbane; Princess Alexandra Hospital); V Liu, E Tan, JST Tefay, R Shen, S Bowman (Brisbane; Queen Elizabeth 2 Jubilee Hospital); D Mitchell, M Kelly, A Edmundson (Brisbane; Surgical Treatment and Rehabilitation Service); H Iswariah, R Franz, M Chandrasegaram, P Yuide, SS Hlaing (Brisbane; The Prince Charles Hospital (TPCH)); S Abeykoon, D Kaushal (Campbelltown; Campbelltown Hospital); C Leung (Canberra; Calvary Hospital); S Davis, NF Franco (Canberra; Canberra Hospital); T Rawther, R McClen, W Petrushnko, E Roussos, K Das (Coffs Harbour NSW; Coffs Harbour Health Campus); S Stevens, F Alnimri, S McClintock, J Maritz (Colac; Colac Area Health); S Hariharan, S Laura, B Wang, V Ng, J Linker, A Li, I Dong, R Bhatia, S Cai, WKH Lai, AC Dawson, SYD Chia, M Binks, N Tran, SHM Ng, D Shen, EWY Lun, E Reid, J Cui, M Roussos (Gosford; Gosford Hospital); M Issa, M Anandan, P Devlin, U Naidoo (Hamilton; Hamilton Base Hospital); B Balaravi Pillai, D Abeysirigunawardana , T Parker, T Valizadeh Elizeh (Melbourne; Angliss Hospital); D Liu, S Ng, J Jones, O Ladlow, JV Maida, D Proud, A Vu, N Shulman, L Bromley, V Muralidharan, K Hall, C Cheong, C Jamieson-Grigg (Melbourne; Austin Hospital); A Hilder, T Manickam, L Barnard (Melbourne; Box Hill Hospital); K Jaffry, A Gray, A Lim, R Kattini (Melbourne; Casey Hospital); M Bickford, S Kenworthy (Melbourne; Knox Private Hospital); A Crowe, J Zhu (Melbourne; Maroondah Hospital); M Pacilli, A Comella, K Taghavi, R Nataraja, SJA Robinson (Melbourne; Monash Childrens Hospital); D Lowen, A Khan, S Samadi, S Tan, AL Surkitt, S Condron, E Haege, E Francis, A Boynes, S Gill, B D’Souza, H Xiao, E Fraser, J Wong, S Fennelly, K Mori, M Muir, Y Huang, R Pajtak (Melbourne; Northern Hospital); J You, C Banal, T Abelman, N Chen, L Chong, H Jalilehvand, W Santucci, B McKay (Melbourne; St Vincent’s Hospital); I Murshed, D Makary, L Green, M Wichmann, A Lim, M Kang, EA Dontoh (Mount Gambier; Mount Gambier and Districts Health Service); P Walker, A Fani, S Sundararajan, E Downes, A Davis, M Bajwa, R Geow, KK Sim (Mount Nasura; Armadale Health Service); S Smith, L Peters, S Zhang, ACK Cheung, I Caitens (Newcastle; Calvary Mater Newcastle); M Ishak, E Zhang, KKA Yu, L Beukes, L Kang (Newcastle; John Hunter Hospital); M Bowles, E Downing, B Williams, G Cox, V Bakshi (Robina; Robina Hospital); N Ensor (Sale; Sale Hospital / Central Gippsland Health Service); J Ng, CT Petcu, J Hwang (Southport; Gold Coast University Hospital); TJ Hugh, S Quoy, C Knee, D Siriwardena, C Tung, G Smith (Sydney; Royal North Shore Hospital); O Camilleri, J Vu, J Hong, C Cornwell (Sydney; The Royal Prince Alfred Hospital); E Chan, C Killoran, J Mackenzie, D Fry (Toowoomba; Toowoomba Hospital); V Mahendravarman, A Shanmugalingam, C Li, L Allan, P Shivashankar, H Pleass (Westmead; Westmead Hospital); V Lin, R McGee, B Bereket Araya, CW Un, Y De Silva, D Maan, J Wang, M Kwon, R Kibuuka, J Chew, J Siu, J Barklimore (Wyong; Wyong Public Hospital).

Austria: N Klammer, R Schmidt-Branden, P Tschann, P Horvath (Feldkirch; Landeskrankenhaus Feldkirch); N Koter, G Moitzi, E Wallner, C Allmer, F Aigner (Graz; Barmherzige Brüder Krankenhaus, Graz); J Kahn, A Belarmino, R Sucher, V Wolfschluckner, G Singer (Graz; Medical University of Graz); R Függer, M Biebl, A Punzengruber, H Fehrer (Linz; Ordensklinikum Linz Elisabethinen); A Binder, E Haiden, P Riedl, M Enßlin (Tulln; Universitätsklinikum Tulln).

Bangladesh: K Nahar, T Akter (Dhaka; Dhaka Medical College Hospital); A Oosterkamp (Dinajpur; Lamb Hospital).

Benin: G Gbessi, J Avakoudjo, M Fiogbe, P Assouto, SP Chigblo (Cotonou; Centre National Hospitalier et Universitaire Hubert Koutoukou Maga); H Aouagbe Behanzin , M Seto, G Mevognon (Cotonou; Hopital de Menontin); M Agbadebo, A Hada, SFA Houndji (Dassa-Zoumè; Hôpital de Zone de Dassa-Zoumè); E Bara (Kandi; Hôpital de zone de Kandi); AB Yevide, ZF Tamou, E Hatangimana, B Cakpo , R Soglonou (Klouékanme; Hopital de Zone de Klouékanme); TK Hessou, SR Tobome, M Zounon (Natitingou; Centre Hospitalier Départemental de l’Atacora); AM Hodonou, C Bokossa , F Hounde , R Alinde (Parakou; Centre Hospitalier Universitaire Borgou Alibori); F Dossou, R Goudou, ACS Toi , G Natchagande (Porto Novo; Centre Hospitalier Universitaire et Departemental Oueme Plateau).

Bosnia and Herzegovina: M Stjepanovic (Doboj; Genera Hospital ‘Sveti aposto Luka’ Doboj); O Čančar, M Pejović (Foča; University Hospital Foča); J Miskovic, M Boras, M Kajic, V Dragisic, Z Brekalo, I Mikulic, N Soldo, M Bevanda, M Faletar (Mostar; SKB University Clinical Hospital Mostar); M Salibašić, E Hodžić, E Halilović, M Kruščica (Sarajevo; Clinical Center University of Sarajevo); A Cerovac, H Škiljo, E Hodžić, O Bedak, M Kalabić, E Begunić (Tešanj; General Hospital Tešanj); A Huremovic, E Alić (Tuzla; University Clinical Center Tuzla).

Brazil: RA Tenfen Carneiro (Francisco Beltrão; Universidade Estadual do Oeste do Paraná).

Bulgaria: D Georgiev, I Fidoshev, V Neykov, E Daleva, I Ilieva (Pleven; Heart and Brain - Pleven Hospital); M Karamanliev, D Dimitrov, A Shanker, P Vladova, MD Shoshkova, A Mehta, M Abdullahi, V Ratheesh, V Kamalathevan, C Wiesner, S Shittu, M Galasyuk, S Shanker (Pleven; University Hospital Dr Georgi Stranski, Medical University - Pleven); M Imirski, A Soumpasis (Plovdiv; MHAT St. Karidad); E Hadzhieva, D Chakarov (Plovdiv; UMHAT Sveti Georgi); T Yotsov, P Kamenova, A Vricheva, I Yotsov (Ruse; University Hospital Medika); E Hristova, K Spassov (Sofia; Fifth City Hospital Sofia - 5th MBAL).

Burkina Faso: A Sanou, M Windsouri, R Doamba, IW Bahikoro , AST Sanon (Ouagadougou; Tengandogo University Hospital).

Burundi: N Ildephonse, N Steve , Y Fulgence , GD Nibogora, C Nimbona , B Paul (Bujumbura; Centre Hospitalo-Universitaire de Kamenge); G Kazobinka, C Rukundo, N Renovat, E Ndizeye , M Dauphin (Bujumbura; Kamenge Military Hospital); FF Irakiza, L Niyidukunda, G Nkunguzi, N Oscar, N Theophile, B Révérien (Bujumbura; Prince Regent Charles Hospital).

Cambodia: S Oum, S Eam (Battambang; Handa Medical Centre).

Cameroon: NS Bibila, NN Cabrel, J Dongmo (Bamenda; Nkwen Baptist Hospital).

Canada: R Spence, G Berger, D Hannedige, C Hoogerboord, A Abidali (Halifax; Queen Elizabeth II); M Mozel, L Monteiro, R Lertnamvongwan, D Konkin, R Kaur (Port Moody; Eagle Ridge Hospital); M Mozel, R Lertnamvongwan, L Monteiro, S MacKenzie, D Konkin (Vancouver; Royal Columbian Hospital).

Chile: MM Modolo, E Sepúlveda, Á Molero, M Perez, LV Torres Bavestrello, ÓA Soublett Rivas , C Carrillo Sarango, L Paredes, P Sornoza, A Pinto, C González (Santiago; Hospital Barros Luco Trudeau).

China: J Wang (Guangzhou; The First Affiliated Hospital of Jinan University).

Colombia: L García-Zambrano, PA Cabrera Rivera, N Paez, SV Agudelo Mendoza, MS Mosquera Paz, A Kadamani Abiyomaa, CF Roman Ortega, F Casas J, B Guerra (Bogota; Fundacion Cardioinfantil-IC); JD Molina Marin, C Maya, C Vasquez Maya, B Dieck, F Zapata (Medellin; Clínica CES); VA Ruiz López, MA Ñañez (Popayan; Hospital Susana Lopez de Valencia); DC Cardona Gomez, A Rojas, DC Patiño García , LI Bolaños, C Pastás, DA Pérez Muñoz (Popayán; Hospital Universitario San José).

Croatia: J Mihanovic, I Bacic, D Vukosav , V Žufić, O Jurić, E Dijan, N Jović, I Ćoza, I Rakvin, Z Katusic, T Soric, I Vidić, D Rukavina (Zadar; Zadar General Hospital); I Separovic, R Radojković, J Mavrek (Zagreb; University Hospital Centre Zagreb).

Cyprus: M Theodoridou, A Pilavas, R Moukarzel, R Andreou, N Gouvas, K Lambri, S Charitonos , IC Mylona, G Kokkinos, N Dimitriou, MM El Ghoul Miliotou, V Ioannou, NA Ververidis, N Kalampokis, A Yiallourou, R Sokratous, D Tsiardas , P Evangelou, D Evripidou, C Thrasyvoulou (Nicosia; Nicosia General Hospital).

Czech Republic: T Reichelt, P Hudáč (Decin; Krajská zdravotní as - Hospital Decin); K Akter, L Moolla, F Rudisch, A Ibrahim Hassan, O Ahmad, E ELShennawy, M Shalaby, M Khaled, A Akiba, F Philips, E Bankart, R Elshennawy, A Al Kaddah, H Al Atassi, S Ashry, N Salgadoe (Hradec Kralove; Charles University Hospital); L Majerčák , P Levíček, A Lukáč, L Pánči (Ivančice; Hospital Ivančice); J Roman, L Tulinsky, I Mrazkova, P Ostruszka, A Varga, L Martinek (Ostrava; University Hospital Ostrava); H Novák, J Woleský, P Francúz (Prague; Motol University Hospital).

Dominican Republic: R Rivas, B Calcaño, J Michel, Y Perez, R Ubiñas, P Garcia-Dubus, S Batista, S Strachan (Santo Domingo; CEDIMAT - Centro de Diagnóstico, Medicina Avanzada, Laboratorio y Telemedicina).

Egypt: Y Tanas, Y Kerolous, Y El Okazy, M Mokhtar, M Lotfy, M AL Sayed, H Altabbaa, AGMM Abouelnagah, O Al Shaqran, D M. Awad, A Sabry, G Nagy, E Amer, M Khalil, A El Shamarka, B Sharaf eldin, AAA Aboshosha, A Farrag, H Sherif Farouk Ahmed Hassan, Y Badr (Alexandria; Alexandria Main University Hospital); Y Orabi, M Kamal matter, A Alrifaee (Alexandria; Alexandria Medical Research Institute); M Elnour , M Zahran, A Aladl, M Bahnacy, Y Seada, M Kotb, A Ragab, Y Farag, L Khalifa, M Elmiesiry , D Abdalaziz, I Maharem, O AbouHiekal, O Hany, S Hanna, Y Dean, A Faisal (Alexandria; Smouha University Hospital); M Mostafa, I Ali, T Sabra, H Ibrahim , A K. Ali, M Osman , A Eltayeb (Assiut; Assiut University Children Hospital); A Morad, MO Herdan , A Abdelshafi, M M. Nathan, M Shalkamy, M Hamada Takrouney, R Sayad, FA Monib, A A Elhars, MM Saad, A Rashad Temerik, AM Abbas, O Mohamed Mokbel, E AbdElBaset, A Barakat, Z Bady, S Arafa, Z Osama, A Elzanaty , S Salama (Assiut; Assiut University Hospital); MEM Madany (Aswan; Aswan University Hospital, Aswan University); A Khaity , R Adel Diab, A Ghazal, A Ehab, A Abd Elsattar (Cairo; Al Zahraa University Hospital); A El-bastwesy (Cairo; Al-Azhar University Hospitals); A Eisa, M Elesseily, R Radwan, Y Asar, D Waleed, S Tawfik , AF Nixon Fulli , MJI Albert, A Autiak Ayii Chol, AR AbdelHalim, B Azhar, H Al-derume , M Alqadasi, N Alasbahi (Cairo; Cairo University Children’s Hospitals (CUSPH & CUCH)); H Abozied, Y Ashour, Y Mohamed , M Abdelmaboud, H Abdelazim, AES El kady, M Omar, A Haty, M Abd Al-Fattah, I Tagreda , IM Kereet, AG Montaser, M Faisal, M Masoud , M ElSayed Metwally (Cairo; EL-Hussein University Hospital, Al-Azhar University, Faculty Of Medicine); ASM Abdelrahman, S Mansour (Cairo; Giza International Hospital); A Nabil, MMA Marei, A Elmosalamy, L ElGebaly, AM Allam, T Awad, H Taher, K Fayed, M Abdelfattah, DH Khattab, N Ali, A Saleh, K Nassim, NK Aly, I Abo Elhagag, M Doss, M Elzayat, Y Samer Morsy, M ElFiky (Cairo; Kasr Al Ainy Faculty of Medicine, Cairo University); M Erfan, M Zaazou , M Reda, M Kouta, M Mohamad Amin, I Guirguis, H Amir , S Aboseif, M Abdelhafez, O Agha, A Khairy , A Dawoud, M Hamoud Almahly, S Mostafa Yassin , A El-Sherbiney, A Adel, H Foda, D Ahmed, AS Elkhodary , AA Mansour (Giza; The Memorial Soaad Kafafi University Hospital); A Elghrieb, M Natey, A Elshazli Mahmoud , A Khalleefah, H Elfeki, M Shalaby, M Sadek, M Abdelmaksoud, M Mostafa, M Waseem, A Adel, A Azam, A Sakr, A Sanad, م عبدالفتاح (Mansoura; Mansoura University Hospital); H Foad, S Elnoamany, S Selim, DS Alrokh, A Hassanin (Menofia; Menofia University Hospital); M Alansary (Qena; Qena University Hospital); A Ragheb, M Fahmy , M Mehanny (Sohag; Sohag University Hospital); AGE Aboelnasr, KMG Mohammed, M Eissa, S Allam, M Kamar (Tanta; Tanta University Hospital); A Asla (Zagazig; Al Ahrar Zagazig Teaching Hospital).

Ethiopia: F Terefe, WA Zerefa, E Gallo , A Yingess, T Kebede, M Mesfin, G Alemayehu , SM Djote, T Girma, DA Muhie (Addis Ababa; Yekatit 12 hospital medical college); E Yeshialem, G Seyfu, F Tsige, A Yeshitila (Deberebirhan; Hakim Gizaw Hospital); M Worku, S Lakew, Y Melkamu, ST Workineh (Dessie; Dessie Referral Hospital); B Mengesha, G Getachew , I Tesfahun, M Teressa , BG Chiman, AB Aregawi (Hawassa; Hawassa University Comprehensive Specialized Hospital); N S.Bayleyegn, YY Metaferia, TG Moges, D Mengiste, A Teshome Sahilemariam (Jimma; Jimma University Medical Center); B Sime, T Jemal (Yirgalem; Yirgalem Hospital Medical College).

France: E Volpin, H Braham, C Lionel, R Arena, Y Malki (Eaubonne; Hôpital Simone Veil); M Bertrand, A Castaldi, L Theuil, A L’Hostis, M Prudhomme (Nimes; Hôpital Carèmeau); P Riva, A Lapergola, D Mutter, S Perretta (Strasbourg; Nouvel Hopital Civil de Strasbourg).

Gabon: PC Nze Obiang (Libreville; Centre Hospitalier universitaire mère enfant Fondation Jeanne Ebori).

Georgia: Z Demetrashvili, G Devidze, G Pisarevi, L Petashvili, E Ekaladze, N Lekiashvili, I Pipia, A Tvaladze, G Kenchadze, K Khutsishvili (Tbilisi; N.Kipshidze Central University Clinic).

Germany: LD Lee (Berlin; Park-Klinik Weissensee); J Binder, A Denz, C Krautz, M Brunner, M Maak, GF Weber, A Stollberg, D Hackner, S Engel, F Krämer, R Grützmann (Erlangen; Universitätsklinikum Erlangen); M Schüler, J Kleeff, R Rüdrich (Halle; University Hospital Halle); A Kirschniak, J Rolinger, S Göller, L Van den Hil , J Miller, H Pehlivan (Moenchengladbach; Kliniken Maria Hilf); M Kießler, N Hüser, D Schippers, M Berlet, M Steffani, M Weber (Munich; Klinikum Rechts der Isar TUM School of Medicine); N Börner, M Albertsmeier, H Arbogast, M Mattis, S Jarmusch, P Zimmermann, U Wirth (Munich; Ludwig Maximilian University of Munich - Großhadern); F Anzinger (Munich; Ludwig Maximilian University of Munich - Innenstadt); FG Bader, M Sohn, ML Koschke, M Busch , N Hielscher (München; Isarklinikum); A Brosin, J Lindert, M Philipp, M Gumsheimer, F Wiese (Rostock; University Hospital Rostock); L Sahan, GA Stavrou, J De Deken, M Jabal (Saarbruecken; Klinikum Saarbruecken); MW Löffler, A Königsrainer, M Quante, C Yurttas (Tuebingen; University Hospital Tuebingen).

Ghana: R Armah, NA Christian, D Daary, S Akuffo, A Twumasi, AD Andani , J Oppong, E Agbowada, J Daleku, J Ampadu, W Afedo, Z Robertson, A Obbeng, DN Lee, D Ofosuhene (Accra; Greater Accra Regional Hospital); GD Brown, F Osman, FJ Eshun, C Banka, I Amankwaa, E Ametefe , G Owusu, J Nyamekye - Baidoo , O Okrah, G Birikorang, P Kumassah, J Dei-Asamoa, J Annan, C Akli-Nartey , D Alifoe , S Tsatsu, C Ansah Larbi, U Una, K Yalley, A Bediako Bowan (Accra; Korle-Bu Teaching Hospital); KB Oduro-Boateng , S Anim , H Adjei, S Dognia , A Oppong , C Markin, JL Ahale, NKA Obuobi (Accra; Pentecost Hospital); S Agana , EB Akakpo, F Galley (Ankaful; Ankaful Leprosy General Hospital); FE Gyamfi, S Segnitome , S Agordjor , D Adjei, D Kyeremeh, Y Sarpong, F Opoku Twene, CK Ntow-Boahen, SAA Atupra , V Siepaal , IN Bakaweri, AJ Tabiim , R Agyei Boakye, A Asare Twumasi (Berekum; Berekum Holy Family Hospital); R Akankoatuesi Apatewen, U Kanyan Kassim , B Owusu Ansah, F Amoako (Bolgatanga; Upper East Regional Hospital); V Kudoh, K Boakye-Acheampong , B Boakye, R Kpangkpari, MT Morna, GA Rahman , EO Ofori, L Adagrah Aniakwo, M Amoako-Boateng , D T. Enti, S Debrah, M Nortey, P Koggoh , P Mensah, MM Agyapong, T Agyen, V Etwire, Y Adofo-Asamoah , S Yussif, M Yigah (Cape-Coast; Cape Coast Teaching Hospital); B Maanikuu, F Kuubetersob B. N, D Powell, A Gbeadese , F Tierenye (Damongo; St. Anne’s Hospital); EA Nachelleh, DYD Agbley , N Jiagge , R Akpaka, D Labadah, N Naabo, R Guzmán Lambert, FJ Eshun, P Ntem, E Setsoafia, N Affram, BY Hernandez Cervantes, DB Osei, K Ewool, F Nyarko, MA Ali, MA Oyortey, I Hagbevor, ME Ashong , JN Anyorigiya (Ho; Ho Teaching Hospital); J Yorke, A Lovi, EO Osei, PA Boateng, R Oppong-Amoah, K Agbedinu, C Dally, SG Brenu, F Galley, FM Agbemafoh, I Kyei, C Aboah, AY Appiah-Kubi, B Nimako, M Aikins, M Adinku , A Opoku-Agyapong, J Adjei, R Sagoe (Kumasi; Komfo-Anokye Teaching Hospital); A Gyedu, PK Boateng, S Mensah, E Frimpong-manso (Kumasi; University Hospital, KNUST); P Taah-Amoako, S Tabiri (Nsawkaw; Tain District Hospital); F Owusu, P Yeboah Owusu (Sunyani; Brong-Ahafo Regional Hospital); EMT Yenli, AS Seidu, M Dason , M Amadu, GA Adoro, M Kyereh, I Osman , J Quansah, C Doku, A Darkwa Boateng, AM Muntaka, MA Dokurugu, R Nesco, M Yahaya, EDF Konlan, A Issaka, RA Ramirez Calas , M Sheriff, M Dery, V Dassah (Tamale; Tamale Teaching Hospital); BK Seshie, F Caiquo, M Dum, L Ackam, K Yalley, FDA Agbodo, A Baiden Amissah, D Ashitey , LD Bray, J Ofori, M Ishak (Tema; Tema General Hospital); G Ansong (Walewale; Walewale Government Hospital).

Greece: I Gogoulis, K Bekiaridou, A Mitsala, S Botaitis, C Tsalikidis, M Asimakidou, C Nikolaou, E Efremidou, C Limas, P Chloropoulou, M Aggelidou, M Pitiakoudis, P Kostoglou, G Pappas Gogos, M Karanikas (Alexandroupolis; Alexandroupolis University General Hospital); E Kapasakis, M Karakeke, A Skarpas, C Floros, K Athanassiou, E Karakeke (Amfissa; General Hospital of Amfissa); N Tasis, A Sarafi, A Plastiras, G Kavalieratos, T Tsirlis (Athens; Agios Savvas Anticancer Hospital); L Chardalias, N Memos, V Themelidi, I Papaconstantinou, T Theodosopoulos, D Politis, K Iliakopoulos, K Bramis, P Antonakis, A Skreka, D Kotsaris, N Dafnios, A Vezakis, I Contis, T Petropoulou, KC Kordeni, T Kozonis, G Fragulidis, D Massaras (Athens; Aretaieion Hospital); E Apostolopoulos, I Karatsolis, A Mourtzouni, K Avgerinos, D Kelgiorgi, K Polychronopoulos, G Kostoulas, A Tsechpenakis, A Saridaki (Athens; Athens Euroclinic); A Ioannidis, C Chouliaras, I Tierris, MK Konstantinidis (Athens; Athens Medical Center); DK Manatakis, D Balalis, N Stamos, N Tasis, V Kalles (Athens; Athens Naval and Veterans Hospital); T Sidiropoulos, M Papadoliopoulou, N Arkadopoulos, D Sampanis, P Vassiliu, E Dylja, AI Nikolaou, I Margaris, P Kokoropoulos, V Tsaousis, S Christodoulou, E Poulios, A Chamzin (Athens; Attikon University General Hospital); S Kapiris, E Mavrodimitraki, M Sotiropoulou, N Dimitrokallis, N Papadogianni, V Vougas, M Papamichail, K Rekouna, K Pavlopoulos, N Roukounakis, A Thanasa, P Trakosari, M Christou, E Saitoglou (Athens; Evaggelismos General Hospital); C Nastos, D Dellaportas, P Lykoudis, N Garmpis, G Kouraklis (Athens; Evgenideio Hospital); P Christodoulou, G Kapogiannatos, A Nikitaras, SM Tsoti, J Katogiritis (Athens; General Hospital Asklepieio Voulas); EC Tampaki, C Papazacharias, O Bellou (Athens; KAT Athens General Hospital); N Machairas, P Dorovinis, C Doudakmanis, D Schizas, A Syllaios, MD Keramida, P Stamopoulos, S Davakis, M Despotidis, A Panagakis, S Kykalos, F Stavratis, M Vailas, L Karydakis, N Kydonakis, A Loizou, KS Giannakopoulos, P Sakarellos, A Kozadinos, I Katsaros (Athens; Laiko University Hospital); C Damaskos, E Antoniou, M Mavri, I Psilopatis, S Vernadakis , I Bokos, P Paraskeva, D Vardakostas, I Gomatos, A Barlas, A Smyrnis, D Prevezanos, NN Mathioudakis, I Kozadinos, A Kozadinos, P Kanavidis, N Garmpis (Athens; National and Kapodistrian University of Athens); E Spartalis, M Spartalis (Athens; Sotiria General Hospital of Thoracic Diseases); C Stefanou, S Gkogkos, L Fountoulis, I Sougkas, M Billis, A Kontokostopoulos, A Balta, T Padioti , MA Sotiriou, A Theochari (Filiates; General Hospital of Filiates); N Tsakiridis, I Tsakiridis, E Synekidou (Florina; Florina General Hospital ‘Eleni Th. Dimitriou’); E Athanasopoulou, C Ntagkas, E Samara, A Katsiou (Ioannina; University Hospital of Ioannina); D Panagopoulos, A Panagopoulos, L Katsiaras (Kyparissia; General Hospital of Messinia, Hospital Unit of Kyparissia); C Kolla, L Mansour, G Koukoulis, S Zourntou, D Papageorgouli, K Bouliaris, LI Fountarlis, M Bei, AA Kalidis, X Vagena, A Gkouniaroudi, A Migdanis, A Bakalis, E Gavriil (Larissa; General Hospital of Larissa ‘Koutlimpaneio and Triantafylleio’); K Koumarelas, MN Kouliou (Larrisa; General University Hospital of Larissa); K Bouchagier, F Mulita, G Verras, G Skroubis, I Maroulis (Patras; General University Hospital of Patras); V Mousafeiris, A Panagidis (Patras; Karamandaneio Prefecture Children Hospital of Patras); A Papadopoulos, P Grivas, F Spanos, A Kalogeropoulou , G Zeringa, C Kourouniotis, G Rados, E Barkolias , K Zakkas, I Demiris, V Nikolaou, K Tata, G Karakaidos (Piraeus; General Hospital of Nikaia); E Kontis, E Papamattheou, A Efstathiou, E Efstathiou, N Kopanakis, K Ntatsis, I Katsaros, P Manikis, V Tselepidis, M Kyriazi (Piraeus; Metaxa Cancer Hospital); I Siannis, N Kouzakos, V Georgilaki , N Vlachakos, S Vederaki, A Tsiaka, A Zarafidou, N Zampitis, S Tsatsos, F Stefou, F Kyramargios, M Merrakos, G Bekakos, A Marinis (Piraeus; Tzaneio General Hospital); O Ioannidis, E Anestiadou, K Zapsalis, S Simeonidis, S Bitsianis (Thessaloniki; George Papanikolaou General Hospital of Thessaloniki); M Drogouti, A Gkoutoula, A Sarakatsanos, E Efthymiou, I Chatzis (Thessaloniki; O Agios Dimitrios General Hospital); I Spyridakis, C Kaselas, M Tsopozidi, M Florou, C Demiri, V Papadopoulos, D Giakoustidis, A Giakoustidis, D Alexandrou, P Chatzikomnitsa (Thessaloniki; Papageorgiou General Hospital); SC Liapis, K Perivoliotis, C Chatzinikolaou, N Tsantikos, ZR Karampotaki , D Lytras (Volos; Achillopoyleio General Hospital of Volos).

Guatemala: M Aguilera-Arevalo, M Rodríguez-Ordoñez, DA Sosa Méndez, M Sebastián-Mendoza, TA Salazar-Lorenzana, R Herrera, DE Reyes Rodríguez, P Vásquez , S Morales , J Gomez , C García-Salas, JR Asturias Luna, J Tabora-Zepeda, S Vasquez, JR Hernández , JOM Herrera Batres , D Muñoz, E Ayala , O Coyoy-Gaitán (Guatemala City; Hospital General San Juan De Dios); JB Pellecer Cano, DA Palma Portillo, M Blanco (Guatemala City; Hospital Juan Jose Arevalo Bermejo); D Herrera, SA Villeda , O Lima Azurdia (Guatemala City; Hospital de Referencia Nacional de Enfermedades Respiratorias).

India: S Jp, M Bhat, A Raheja, I Shariff, H Anand , B R Budihal, S Kashyap, B Arya, Y M S, N Krishnappa (Bangalore, Karnataka; BGS Global Institute of Medical Sciences); M Manangi, P Anandan, S Shivashankar chikkanayakanahalli, S Kumar Venkatappa (Bangalore; Victoria Hospital); TS Mishra, P Kumar, M Gureh, MK Sethi, AA Asharaf (Bhubaneswar; All India Institute Of Medical Sciences - Bhubaneswar); Y Sakaray, S Irrinki, S Subbiah Nagaraj, S Khare, C Tandup (Chandigarh; Postgraduate Institute of Medical Education & Research, Chandigarh, India); M K B, AB Muthunayagam, A Prasath S V , P Arunachalam, P John (Coimbatore; PSG Institute of Medical Sciences and Research); S Raul, R Vakil, R Sinha, E Dvivedi, A Thomas, S Joseph, A Sharma, B Khan, D Chatterjee (DELHI; St Stephen’s Hospital); R Gupta, A Khanduri, S Singh, DH Tyagi , U Daspal, N Rawal, R Varshney (Dehradun; Synergy Institute of Medical Sciences); M Luthra, R Handa, S Basu , P Chadha, R Sethi (Delhi; Holy Family Hospital); T Longkumer, KK Mishra, S Sundaramurthy, S Kumar, D Phom, J Kaippally, D Ommi, L Imchen, T Alinger (Dimapur; Christian Institute of Health Sciences and Research); A Chhabra, A Kumar (Faridkot; Guru Gobind Singh Medical College & Hospital (Baba Farid University of Health Sciences)); B Kharga, M Sarda, K Bhutia (Gangtok; Sir Thutob Namgyal Memorial Hospital Sochakgang); N Sharma, MS Rodha, N Banerjee, A Baksi, S Kaur, R Chaudhary, M Lodha, SP Meena, M Badkur, I Singh, A Sinha, KJ Rathod, R Saxena, J Tk, A Vig, M Pathak, A Sukhdev Jadhav, S Nayak, T Motiwala, K Shreyas (Jodhpur; All India Institute of Medical Sciences (AIIMS), Jodhpur); SR Pathan, J Rathod, C Agarwal, K Sharma, S Pandya (Karamsad; Shree Krishna Hospital); A Anand, A Kumar, HS Pahwa, AA Sonkar, MK Agrawal, AK Pal (Lucknow; King George’s Medical University); D Jain, PD Haque, V Michael, W Bhatti, J Dhiman, DRS Thind, A Bhatt, P Gupta, A Luther, S Khurana, RR Ranadive, A Suroy, H Kaur, S D A, P Shukla (Ludhiana; Christian Medical College & Hospital); NK Chaudhry, DA Hajela, P Patel, PK Arya, DD Dhawan, DR Tripathi, KK Luthra, A Kumar, DH Gupta (Ludhiana; Satguru Partap Singh Hospital); A Mathew, C Pun, P Dummala, M Gurung (Madhepura; Madhepura Christian Hospital); P Alexander, N Aruldas (Manali; Lady Willingdon Hospital); PS Prabhu, S Payyanur Thotan, B L, B Sv (Manipal; Kasturba Medical College Hospital, Manipal); RD Sharma, R Redkar, R Nathani, S Karmarkar, A Sharma, S Singh, S Achugatla, A Bangar, DD Kulkarni, K Raghuwanshi, DN Nikam (Mumbai; Lilavati Hospital & Research Centre); B Sarang, D Belekar, K Gaikwad (Mumbai; Terna Medical College and Hospital); A John, PA Thomas, L Pramod, D Gavit, D Singh (Nandurbar; Chinchpada Christian Hospital); N Kansakar, N Gupta, N Kapur, N Narain (New Delhi; ABVIMS Dr RML Hospital); S Kulkarni (New Delhi; Army Hospital Research & Referral New Delhi); T Rashid, M Husain, F Tauheed, SV Manzoor , S Ohri (New Delhi; Hamdard Institute of Medical Sciences & Research); L Bains, P Lal, S Neogi, A Mishra, S Ahuja (New Delhi; Maulana Azad Medical College); T Iahmo, M George, M Singh , P Waghchoure, A Choudhrie (Padhar; Padhar Hospital); A Kumar, M Aggarwal, V Kanna D, S Vembar, TP Singh, DS Walia, V Singh, G Kaur, A Jindal, P Dhamija (Patiala; Government Medical College Patiala); M Kumar, A Sinha, AK Jha, M Sharma, A Bhadani (Patna; All India Institute of Medical Sciences, Patna); R Abhinaya, U Kumbhar, A Jain, S Chilaka , S P (Pondicherry; Jawaharlal Institute of Postgraduate Medical Education and Research); VS Jha, A Jayapalan, Y Vashishth, G Jalal, VV Nair, C Raphael , HK Prabhakar, Z Khan (Pune; Command Hospital, Southern Command); D Dugar, D Mohanty, TDB Tridip, DR Ramchandani (Raipur; All India Institute of Medical Sciences Raipur); S Basu, N Kumar, AG Goswami, H Panga, D Mallik, A Gupta, D Rajput, R Anjum T Siddeek, P Manjunath, S Edem, F Huda, SK Singh, S Karuppusamy Krishnasamy, S Katragadda, KMR Reddy, I Ahmed, E Yhoshu, L Manoj Joshua, A Das, P Kothari (Rishikesh; All India Institute Of Medical Sciences); K Singh , SS Malhi, R Kaur, HK Cheema, M Singh, N Saini, M Gupta, A Bhatti, A Gupta, P Kaur, N Pahuja , H Kaur, S Chopra, B Sehgal (SAS Nagar (Mohali) ; BR Ambedkar State Institute of Medical Sciences Mohali); DS Kshirsagar, M Kaple, G Saxena, S Dhole, A Bhargava, C Mahakalkar, S Deshpande (Sawangi (Meghe), Wardha; Acharya Vinoba Bhave Rural Hospital); R Wani, RA Dar, AA Malik, N Bhat, ZA Shah, GA Bhat (Srinagar; Sher-i-Kashmir Institute of Medical Sciences); S Kondpan, A Kutma, JA Kalyanapu (Tezpur; Baptist Christian Hospital); A Sundaram, K J B, R Krishna raj, G George, M Chisthi, H Jafarkhan, I P s, U Govindan, MKL K S D, C Narayan, G Pillai, VV Kollengode, D Jabbar (Thiruvananthapuram; Government Medical College Thiruvananthapuram); T Tony V, A Nair, A Kavalakat, A Moncy, A Johnson, A Joseph, A Appukuttan, A J, AN Oommen, S Francis, S M, N Srinivas, M Narayanan (Thrissur; Jubilee Mission Medical College & Research Institute); T Devabalan Koil, MR Jesudason, Y Myla, ASP Dhinakar, A Tirkey, B Roopavathana. S, R Mittal, S Surendran, N Paul Ambrose, D Joshiba, P Trinity, R Raghunath, NP Paul Sigamony, PY George, R Philip Sridhar, S Chase, SJ Arthur (Vellore; Christian Medical College & Hospital).

Iran, Islamic Rep.: N Yousefzadeh Kandevani (Bastak; Farabi hospital); M Pourfridoni, H Askarpour, H Mohammadi sardoo, AA Kheirkhah Vakilabad, M Ali-Hassanzadeh (Jiroft; Imam Khomeini Hospital).

Iraq: R Raheem Attallah Al_obaidy (Anbar; Heet General hospital); Z Alkhuzaie, S Salim , FRH Hassooni (Najaf; Al Batool private hospital); Y Zwain , HMA Oneizah, S Razaq, M Razaq, HHZ .zaini (Najaf; Al-Najaf Al-Ashraf Teaching Hospital).

Ireland: E Colton, KE Oderoha, A Gill, S Ramjit, F Ghazali, K Griffin, R Ahmed, C Weadick, S Nair, V Sharma, C Donohoe (Dublin; St James’s Hospital); R Tummon, B Maguire, S Wrenn, R Habib, P Owens (Kerry; University Hospital Kerry).

Israel: A Shweiki, G Szydlo Shein, G Almogy, Y Mintz, A Pikarsky, R Elazary, O Cohen-Arazi, JA Demma, H Mahajna, B Helou, Y Fishman, G Marom (Jerusalem; Hadassah Medical Center).

Italy: M De Prizio, K Kröning, R Sulce, LM Fatucchi, F Tofani, V Mariottini, R Malatesti, M Scricciolo, GA Pellicano’, A D’Ignazio, A Mazzoni, A Biancafarina, M Angelini, V Borgogni (Arezzo; Ospedale San Donato USL Toscana Sud Est); L Rossi, G Munzi, G Tarantino, M Castrovillari, A Serao , JR Casella Mariolo, G Del Corpo, A Natili, A Iodice (Ariccia; Ospedale dei Castelli (N.O.C.)); S Gargiulo, B Esposito, M Pannullo, L Bracciano, E Marra, A Alberico (Aversa; San Giuseppe Moscati); A Gori, S Cardelli, G Dajti, C Larotonda, IS Russo (Bologna; IRCCS Azienda Ospedaliero-Universitaria di Bologna); J Andreuccetti, S Molfino, D Alberti, G Pignata, G Emiliani, G Boroni, M Ruffoli, M Manfredini, L Sequi, G Zanni (Brescia; ASST Spedali Civili, Ospedale di Brescia); E Locci, M Podda, V Murzi, C Piras , A Carta, A Pisanu, T Pilia, P Marongiu, A Saba, M Pisano, F Campus, E Gessa, E Silanos, A Lai, F Frongia, F Corronca , S Montisci (Cagliari; Cagliari University Hospital); F Cappellacci, C Soddu, GL Canu, F Medas, PG Calò, S Puddu, M Biancu, M Abbas, F Casti (Cagliari; Chirurgia Generale e Polispecialistica, Cagliari University Ospital ‘Duilio Casula’); B Demurtas, A Deserra, F D’Agostino, C Margiani (Cagliari; Santissima Trinità - ATS Sardegna); L Laface, M Casati, M Mariani, S Guarriello, A Balconi (Carate Brianza (MB); Ospedale Vittorio Emanuele III - Carate Brianza); B Scotto, N Laquatra, R Ruccella, G De Angeli (Carpi; Ramazzini); G Gravante, A Chiappini , R Lopatriello, G Mammolo (Casarano; Francesco Ferrari Hospital); C Distefano, G Riccioli, R Granata, M Veroux, DC Centonze, S Costa, R Gioco, D Zerbo, A Licciardello, L Stella (Catania; Azienda Ospedaliero- Universitaria Policlinico San Marco); L Rende, M Osso, D Paglione, F Pata (Cosenza; Azienda Ospedaliera di Cosenza); D Sasia, G Giraudo, D Ribero, S Alberti, V Schirinzi (Cuneo; Santa Croce e Carle Hospital, Cuneo); P Belotti, L Taglietti (Esine; ASST Valcamonica Ospedale di Esine); V Giordano, A Pesce, CV Feo, MC Pignanelli, S Severi (Ferrara; Azienda Unità Sanitaria Locale di Ferrara); F Cammelli, F Natali, M Scheiterle, G Maltinti, L Fortuna, F Coratti, A Manetti , J Martellucci, E Monati (Firenze; Azienda Ospedaliera Universitaria Careggi); L Gabellini, R Fratarcangeli, A Damigella, E Adinolfi, A Anastasi (Firenze; Ospedale San Giovanni di Dio); M Montagna, A Giuliani, G Procaccini, N Tartaglia, F Vovola, D Merlicco, S Schirone , ST Massa, G Pavone, M Pacilli, F Maffei, A Gerundo (Foggia; Ospedali Riuniti Azienda Ospedaliera Universitaria); D Di Pietrantonio, S Quartarone , L Solaini, G Ercolani, L Ragazzini, V Zucchini (Forlì; Morgagni-Pierantoni); M Cammelli, D Scotto Di Carlo, FAN Marin (Garbagnate Milanese; ASST Rhodense - Ospedale di Garbagnate Milanese); A Azzinnaro, A Razzore, A Petrungaro, E Mina, B Sperotto (Genoa; E.O. Ospedali Galliera); G Carganico, D Pertile, D Soriero (Genoa; IRCCS Ospedale Policlinico San Martino); R Diaz, A Luzzi, S Carrabetta, C Meola, D Caruso, F Floris, P Grondona, E Romairone, S Marzorati, F Ré, C Righetti, A Viacava, L Epis (Genoa; Ospedale Villa Scassi); R Sampietro, D Gobatti, C Zandonella (Gravedona ed Uniti; Ospedale Moriggia Pelascini); VP Dinuzzi, U Rivolta, S Luciano, GMF Marini, L Scaravilli (Magenta; Ospedale ‘G.Fornaroli’, ASST-OVEST Milanese); R Magarini, G Saletta, AC Sironi, G Grava, M Mercurio (Melzo; Ospedale Santa Maria delle Stelle, ASST Melegnano Martesana); D Zulian, A Izzo, M Gritti, S Giudici, E Desiato (Milan; Humanitas Research Hospital); M Molteni, L Ottaviani (Milan; IRCCS San Raffaele Scientific Institute, Milan); G Grande, E Mazzotta (Milan; Ospedale Fatebenefratelli e Oftalmico); S Grimaldi (Milan; San Carlo Borromeo); F Brucchi, F Ferraina, S Lauricella (Milan; Sesto San Giovanni Hospital); E Di Marco (Modica; Ospedale Maggiore); LC Nespoli, G De Carlo, EA Baccalini, D Palmisano, A Scacchi, N Tamini, L Ripamonti, M Rennis, C Vitiello, A Davolio, L Degrate, P Masseria, V Brocco, P Chiacchio, M Ceresoli, E Signaroli, A Finocchio, C Fumagalli, M Binda (Monza; Fondazione IRCCS San Gerardo dei Tintori Monza, Scuola di Medicina e Chirurgia, Università Milano Bicocca); M Milone, M Manigrasso, S Vertaldi, A D’Amore, GD De Palma, A Marello, L Fedele, C Sorrentino, D Pignatelli , G Luglio, FP Tropeano, M Cricrì, A Miele, G Aprea, G Palomba, M Capuano, R Basile, G Sorrentino (Naples; Federico II University of Naples); D Rega, A Ottaiano, V Granata, A Belli, F Izzo (Naples; Istituto Nazionale Tumori Fondazione, Pascale IRCCS); G Pellino, D Massaro, V Mosca, F Selvaggi (Naples; Primo Policlinico di Napoli); G Bellio, L Rubin, N De Santis, N Schiavon, A Zerbinati, S Corso, C Cecconi (Padova; Piove di Sacco Hospital); P Venturelli, G Cocorullo, G Carollo, R Tutino, A Bonelli, G Salamone, MP Proclamà, R Guercio, L Licari, N Finocchiaro, G Graziano, G Orlando, G Guercio, M Marcianò, G Galatioto, F Vassallo (Palermo; Policlinico Universitario Paolo Giaccone); G Palmieri, F Banchini (Piacenza; G. Da Saliceto); G Di Franco, F Porcelli, N Furbetta, A Comandatore, S Guadagni, M Palmeri (Pisa; Azienda Ospedaliero Universitaria Pisana); P Ubiali, F Maffeis, J Velkoski (Pordenone; Azienda Sanitaria Friuli Occidentale (AS FO)); M Giuffrida, GE Nita (Reggio Emilia; Azienda Unità Sanitaria Locale - IRCCS di Reggio Emilia); C Marafante, M Garino, SL Birolo , M Dugo, M Pisano, A Borello, R Barone, LD Bonomo, MV Facchino, M Caccetta, E Moggia, MR D’Anna, C Mosca, S Mungo, A Masciandaro (Rivoli; Ospedale degli Infermi di Rivoli); F Tirelli, I Neri , M Aulicino, C Vacca (Rome; Fondazione Policlinico Universitario Agostino Gemelli); M Campanelli, M Grande, L Siragusa, G Sica (Rome; Policlinico Tor Vergata Hospital, Rome); A Mingoli, G Brachini, B Cirillo, S Meneghini, S Giovampietro , G Duranti, F Ciccarone, L Simonelli, I Clementi, B Binda, GB Fonsi, E Spalice , E Cianci, MI Bellini, G Sgarzini, S Sorrenti, E Lori, P Palumbo, D Pironi (Rome; Policlinico Umberto I Sapienza University of Rome); A Amendola, C De Martino, E Bisogno (Salerno; San Giovanni di Dio e Ruggi d’Aragona); S Di Saverio, A Morello, L Lely, I Merlini, G Travaglini , S Sabbatini, M Zambon (San Benedetto del Tronto; Madonna del Soccorso Hospital); D Giulitti, L Barni, GE Poto (Sansepolcro; Valtiberina); D Fusario, L Resca, L Carbone, A Francia, GE Poto, AL Pesce, O Carpineto Samorani, F Roviello, A Ongaro, SA Piccioni, M Gambelli, L Catozzi, M Gjoka, A Bartalini Cinughi de Pazzi, NN Leonelle Lore, G Grassi, F Manasci, V Ricchiuti (Siena; Azienda Ospedaliero Universitaria Senese); E Basile, A Spaziani (Spoleto (PG); San Matteo degli Infermi); V Silvestri, M Favoriti, P Favoriti (Sulmona; SS Annunziata); S Novello, M Piccino, R Baldan, U Grossi, G Zanus, F Scolari, E De Leo, M Brizzolari, A Brun-Peressut, I Hoxhaj, M Scopelliti (Treviso; Ospedale Ca’ Foncello - Università di Padova (DISCOG)); LB Lo Piccolo, E Montanari, D Cianflocca, A Marano, S Galati, SL Gamba, F Velluti, A Caltagirone, M Giuliano , E Potenza, B De Zolt Ponte, D Visconti, VU De Donato, L Capello, S Chaifouroosh Mamagany, C Celano (Turin; Città della Salute e della Scienza); E Donnarumma, C Saviello, R Scola (Vallo Della Lucania; Casa di Cura Prof. Dott. Luigi Cobellis); S Megna, M Berselli, N Palamara, L Liepa, E Ferri (Varese Lombardy; University of Insubria, Ospedale di Circolo e Fondazione Macchi (Varese)); V Gentilino, M Mogiatti, G Farris, N Pasqua (Varese; Filippo Del Ponte Hospital, University of Insubria); A Iacomino (Venezia; Ospedale Civile - Santi Giovanni e Paolo); I Mondi, C Da Lio, F Sulo, E Ciccioli, D Verdi (Venice; Mirano Hospital); M Martorana, M Filardo, L Schiavone (Voghera; Ospedale Civile di Voghera); I Conversano, M Cappiello, G Scialandrone, N Petrarota, R Tumolo (andria; Lorenzo Bonomo); M Angelucci, S Valeri, G Pascarella, A Strumia, R Alloni (rome; policlinico universitario campus bio medico of rome); D Muschitiello, V Morinelli, L Bonello, SG Intini, S Moschella (udine; santa maria della misericordia).

Japan: H Kato, A Horiguchi, D Koike, Y Asano (Aichi; Fujita Health University Bantane Hospital).

Jordan: S Alananzeh, S Al Momani, M Tanashat, O Altobaishat, S Alsmadi, N Al RABADI, L Sweidan (Ajloun; Al Iman Hospital); Z Alnajjar, G Alsheikh, N Mosleh, H Alzuhd, R Alkhatib, H Al-Abdallat, M Aljarawn, T Aloqaili, I Nadi, A Abdllah, O Al-Fahel, R Khalil, M Said, A Qasem, H Al-Fahel, M Hijazi, R Rabah, A Alaqtash, DB Badwan (Amman; Al-Basheer Hospital); Y Alawneh (Amman; Ibn Al Haitham Hospital); B Alrayes, M Salah, M Al-Qannas, H Abu Obead, I Alnimer, Y Alawneh, M Almaletti (Amman; Islamic Hospital); A Khamees, R Yousef Yassin, A Alsheikh, K Al-Shami, E Abu Siam, O Sarhan, KA Sawaftah, MAM Sawaftah, MA Sawaftah, M Sabri Massadi, Z Al-sheikh ali, N Raiq, O Ibrahim, J Al Karmi, M Diab, I Aburumman , AA Altawaiha , M Hasan, A AlZu’bi, L Yasin, B Yacoub (Amman; Jordan University Hospital); R Abu Salah, M Alqedrh , S Abu khousa, MEH Albanna (Amman; Marka Specialty Hospital); R Hussam Yacoub Hattar, D Samardali, R Refaie, L Hijazein , S Hammad, M Barbarawi, S Mamduh, J Al Daradkah, S Samardali , T Alshawabkeh (Amman; Prince Hamza hospital); M Mahafdah, Q Sabbah, SAM Ba-Shammakh, A Al Hammoud, M Bani hani, M Tabaza, H Malkawi, H Haj Freej, D Kasasbeh, B Dweik, K Ayyoub, OR Mahafdah (Ar Ramtha; King Abdullah University Hospital/ Jordan University of Science and Technology); R Hiary, I Shehadeh, L Dyab, R Daradkeh, R Raddad, R Alzu’bi, T Alhaj Hasan , B Alzoubi, M Alsharayri, M Nofal, S Ellouzy, M Al-Masri, S Fakhouri, N Absy, R Suleiman, O Mansour (As-Salt; Al Hussain New Salt Hospital); A Al-fandi (Irbid; Ar Ramtha Govermental Hospital); M Al-Fraijat, N Rabai, A Al-Zubeidy, R Abd Elkareem, S Bani Amer , T Alhusban, S AL-Doghme, R Abd Alkareem, R Damseh, M Alshami , T Majed, S Al Sharie, M Araydah, R Jaba’Teh, F Haddad, O Almomani, L M Mheidat, R Haddad, S Bataineh, S Ababneh (Irbid; Princess Basma Hospital).

Kazakhstan: M Kulimbet, N Maulenov, N Lakhanov, M Ramazanov, A Kiyabayev (Almaty; City Clinical Hospital No.7, Asfendiyarov Kazakh National Medical University); D Amangaliyev, A Shamsutdinova, A Polatbekov (Almaty; JSC ‘Central Clinical Hospital’, Asfendiyarov Kazakh National Medical University).

Kenya: R Parker, E Irungu, A Fadipe, F Ondago, G Waiyaki (Bomet; Tenwek Hospital).

Lebanon: A Khoneisser, A Kachi, B Abboud (Beirut; Hopital Libanais Geitaoui); M Chaccour, G Bechara, N Eshak, R Hleyhel, M Barakat (Jbail ; Maritime Hospital).

Libya: N Lindi, BH Hameed, A Abaidalla, N Mosbah, M Saleh khatab (Albayda; Albayda Medical Center); MAM Elghriani, A Ali, A Fathi, S Qwyder, M Saleh (Benghazi; Al-jalaa Teaching/Trauma Hospital); M Alshamikh, A Alhammali, A Aldurssi, A Gusibat, M Suleman, S Alashhab, M Denini, F Alowjaly, MM Almihashhish, F Elkhafeefi , H Altawati (Benghazi; Benghazi Children’s Hospital); S Elfallah, F Benghalbon, R Michael , M Abosedra, A Ahmayda, H Mftah (Benghazi; Benghazi Medical Center); M Bohlala, M Muragi, S Alneihuom (Darna; Al-Wahda Hospital); A Alkaseek, A Alshiteewi, H Shames, H Bileid Bakeer (Gharyan; Gharyan Central Hospital); N Albahloul, M Abudabbous, A Belkhair, A Abdelmalik, M Assalhi, M Altajouri, A Alailesh , A Alshukre (Misurata; Misurata Central Hospital); B Alazabi, M Alazabi, G Birqeeq, AAY Almugaddami, A Egdeer (Nalut; Nalut Central Hospital); H Embarek, M Bilfaqirah (Sebha; Al-Majd Clinic); M Abdelkabir, S Abdeewi , A Abdalhadi, M Benghazi (Sebha; Aseel Alghad Clinic); H Idheiraj, M Yahmad, M Alfaid, M Abdu, E Abdu, M Khalifa, G Matroud, A Amaigl , H Aldare, E Ali, K Shwail, K Abdulrahman (Sebha; Sabha Medical Center); A Bouhuwaish, A Emran, A Abdraba (Tobruk; Tobruk Medical Center); AE Elzoubi, A Belaid (Tripoli; Alkhalil hospital); A Alragheai, D Omar, S Magrhi, H Farhat, S Alsuwiyah , S Abrayik, S Bensalem, B Algettawi (Tripoli; Metiga Hospital); S Egreara, TMA Abdulmola, I Kandil (Tripoli; Sabratha teaching hospital); F Alshreef, F Elhabishi , M Alsori, L Shawesh , S Timmalah, M Alnuwayli, A Alhamadi, K Ahmed Ibrahim, S Abdullateef, R Altayargh, A Essamei, S Altoume, A Haidar, M Khalil, A Abdulnabi, S Mohammed , A Ghummied (Tripoli; Tripoli Medical Center/ Tripoli University Hospital); E Younes, S Elfurdag , A Ali, S Ashini, M Edeeb, Z Al-azher El-hamel, M Akkawe , N AlWAER, A Khair Etareig (Zawia; Zawia Teaching Hospital); B Allbakosh, H Abusnina, A Awidan, L Alokshi, M Iqreewi , M Almahjoub , H Altounsi, M Almaqrahi (Zliten; Zliten Teaching Hospital).

Lithuania: E Dainius, S Bradulskis, E Margelis, A Mačiulaitytė, A Subocius, A Parseliunas, E Kubiliute, D Zuikyte, J Kutkevičius, J Vaitekūnas (Kaunas; LUHS Kaunas Hospital); L Venclauskas, K Jasaitis, M Jokubauskas, Z Dauksa (Kaunas; Lithuanian University of Health Sciences Kaunas Clinics); A Gulla, E Daukšaitė (Vilnius; Vilnius University Hospital).

Madagascar: MJ Rakotonaivo, CF Rahantasoa Finaritra, JB Razafindrahita, YM Razafimandimby , A Rakotondrainibe (Antananarivo; Joseph Ravoahangy Andrianavalona Hospital).

Malaysia: AD Zakaria, MIS Ismail (Kelantan; Hospital Universiti Sains Malaysia); R Noor, M Che yaacob, SF Moh Pauzi, Z Chin (Kota Bharu; Hospital Raja Perempuan Zainab II); K Voon, JH Fu, JH Lim, SA Theivendran, NN Ramli (Kuching, Sarawak; Sarawak General Hospital); M Fitri, MA Yunus, AN Ramly, A Md Yunos, AAA Anuar (Malacca; Hospital Jasin); NS Abd Ghani, AAH Ahmad Zaidi, F Ashraf, M Mahadi, AA Abdul Rahim (Serdang; Hospital Pengajar Universiti Putra Malaysia (HPUPM)).

Mali: OAA Dicko, A Dembele, H Dolo, D Kone (Ségou; District Hospital of Tominian).

Malta: C Cini, A Sultana, M Farrugia, J Schembri Higgans, S Bowman, J Psaila, P Andrejevic, S Brincat, K Muscat, M Sammut, R Abela, M Zammit Vincenti, L Casingena, J Galea, M Portelli, M Sammut, N Spiteri, K Iles, R Cachia, D Hili (Msida; Mater Dei Hospital).

Mexico: TR Ibarra-Hurtado (Guadalajara; Antiguo Hospital Civil de Guadalajara); LA Flores Chávez, JA Flores Prado, K Jasso García, NE López Bernal, EV Romo Ascencio, LM Flores Chávez, MP Mellado Tellez, SA Ibarra Camargo, G Delgado Hernandez, JA Guzman Barba, LA Rea Bocanegra, M Tello Jimenez, JA Tavares Ortega, E Gómez Mejía, I Esparza Estrada, AA Salinas Barragan, JA Jimenez Flores, SJ Vázquez-Sánchez, J Gonzalez Garcia , ZM Correa López, FJ Barbosa Camacho (Guadalajara; Clínica de Especialidades más Centro de Cirugía Simplificada); CM Nuño-Guzmán, AM Nava Franco, JF Martinez Martin del Campo , JJ Ulloa Robles , L Bravo, ME Gonzalez-Gonzalez, FD Romo Rosales, TR Ibarra-Hurtado, LG Peña Balboa, C Yanowsky-Gonzalez, R Santana Ortiz, SA Trujillo Ponce, J Orozco-Perez, MDC Gonzalez, JE Gonzalez Aboytes, J Pizarro Lozano , JE Orozco Navarro, F Ibañez Ortiz , O Montaño Angeles, M Calderon, F Diaz , M Lazo Ramírez , JA Aguilar (Guadalajara; Hospital Civil Fray Antonio Alcalde); A Gonzalez Ojeda, C Fuentes Orozco, JM Chejfec-Ciociano, JM Carranza Rosales, MA Sánchez Audelo, CI Lupercio Figueroa, KV Ascencio Diaz, CE Gutierrez de la Rosa, F Mercado Sanchez, FY González Ponce, R Mares País, C González Baez, LÁ Pelayo Orozco, NG Barrera Lopez, A Ramírez Beas, MÁ Zaragoza Mendieta, MF Zarate casas , SL Trejo Ramos, P Salas Núñez, JA Gutiérrez Gómez (Guadalajara; Hospital de Especialidades, CMNO-IMSS); G Ambriz González, I Cabrera, HB Moya- Ambriz , FJ Silva Rivera, EM Torres De Anda, A Hernández, FJ León Frutos, VE Armenta Tapia, M Nieto Galvan, JM Alvarez Hernandez (Guadalajara; UMAE Hospital de Pediatria Centro Medico Nacional de Occidentes IMSS); AI Sánchez-Terán, N Muñoz Montes, AN Fuertes Muñoz, RL Smolinski kurek (León; Hospital Regional e Alta Especialidad del Bajio); K Bozada-Gutiérrez , A Nuñez Venzor, A Zubillaga-Mares, I Serrano (Mexico City; Hospital General Dr. Manuel Gea González); C Moreno-Licea, S Anaya Sanchez, A Trigos Díaz, CJ Pérez - Padrón, EY García-Villegas., RH Perez-Soto (Mexico City; Instituto Nacional de Ciencias Médicas y Nutrición ‘Salvador Zubirán’); MJ Rueda Medécigo, A Leon -del- Angel, HDJ Pérez Baca, C Chavarría Noya , L Castro (Pachuca; Sociedad Española de Beneficencia); D Herappe , MT Barrio Renteria (Querétaro; Hospital de especialidades del niño y la mujer); A Ramos-De la Medina, L Martinez, II Durán Sánchez , DS Gonzalez , MJ Martínez (Veracruz; Hospital Español Veracruz).

Morocco: M Berrakkouch, F Hourri, A Benmansour, R Ait Ben Addi , M Melouane, A Tariq, O Boujidi, I Zerrouq , M Katif, S Amahmid, S Errami, S Jamil, O Nouhail, H Essalim, A Nidali, N Ouachou (Marrakech; Centre Hospitalier Universitaire Mohammed VI, Marrakech); A Aboumedian , S Kessab, N Lahnaoui, O Arsalan, S Kassad, A Hrora (Rabat; Centre Hospitalier Universitaire Ibn Sina Rabat).

Namibia: JT Abebrese, M Van der Colf, FW Quayson , P Shimbulu, P Nambala (Windhoek; Windhoek Central Academic Hospital).

New Zealand: S Rennie, A Herewini (Masterton; Wairarapa Hospital); S Kosna, A Olsen, J Gillingham, R Campbell, A Lin (Wellington; Wellington Regional Hospital); T Uiyapat, S Beavis, D Bardsley, M Lill (Whanganui; Whanganui Hospital); J McNab-Hand, O Ray, C Harmston (Whangarei; Whangarei Hospital).

Nigeria: A Adeyeye, A Akinmade, E Afeikhena, AI Okunlola (Ado Ekiti; Afe Babalola University Multi-System Hospital); D Idowu, J Olorunfunmi, A Olabode (Ado-Ekiti; Ekiti State University Teaching Hospital); N Oloko, KJ Bwala, A Ningi (Bauchi; Abubakar Tafawa Balewa University Teaching Hospital Bauchi); P Agbonrofo, D Osifo , P Idjerhe , BO Izedomi, O Omoike, O Irowa, S Ideh, J Enaholo, C Agbonrofo , O Emuze , PV Odigie, M Ediale, RA Eghonghon, M Edena, A Ekpeti , M Momoh, C Osime, O Osagie, A Arekhandia, M Ibadin (Benin City; University of Benin Teaching Hospital); T AbdulRahman, R Ediru, J Abutu John, O Owolanke (Bida; Federal Medical Centre Bida); U Ezomike, N Agugua-Obianyo, J Ede, S Aliozor, EI Nwangwu, C Ilo, C Amah, L Onyebulu, I Ugwueke , I Obianyo, C Onwuzu, U Dilibe , I Orji, V Enemuo, N Celestine , N Ekwo (Enugu; University of Nigeria Teaching Hospital); SA Sani, S Olori, I Pius Ogolekwu , O Attawodi , R Hauwa SANI, P Chimezie Andrew (Gwagwalada; University of Abuja Teaching Hospital); A Ishola, O Ayandipo, N Akinbami, A Fakoya, TA Lawal, V Osoka, H Ogundipe (Ibadan; University College Hospital); A Ademuyiwa, F Alakaloko, O Oluseye , N Duru, M Ojo, T Olobatoke , AO Lawal, C Nwanmah , O Alaba, R Eloka, C Bode, O Elebute, J Seyi-Olajide, K Onyekachi, O Balogun, O Christianah, L Omomeji , F Akinwande, A Damola-Okesiji, J Okei (Idi Araba; Lagos University Teaching Hospital); H Abiyere, F Oluwafemi, M Babatunde, O Babatunde, AI Okunlola (Ido Ekiti; Federal Teaching Hospital, Ido Ekiti); OM Williams, O Faboya, C Ónyeka, F Oni, K Shodunke, G Eke, M Abdulsalam , O Oso, A Ayodele, M Okechukwu (Ikeja; Lagos State University Teaching Hospital); C Adumah, A Talabi, O Oyinloye, O Olajide , V Agbakwuru, A Aderounmu, A Agbaje, YL Balogun, MO Ameen, DO Komolafe, O Olasehinde, O Ajiboye, M Fagbayimu, G Aduroja, O Fasoro, A Adisa, AM Olugbami, H Oyinlola, E Adebunmi (Ile-Ife; Obafemi Awolowo University Teaching Hospitals Complex); T Mohammed, A Lawal, F Bello, P Adebayo, O Salako, A Akinkuolie, MA Adetoyi, A Akeem Aderogba, T Oyeyemi , O Ojo (Ilesa; Obafemi Awolowo University Teaching Hospitals Complex Wesley Guild Hospital Unit); E Osaze, H Ekwuazi , I Ogundele, P Elemile, A Ayeni, I Okoro , C Onuoha (Ilishan-Remo; Babcock University Teaching Hospital); M Mobolaji-Ojibara , J Mohammad mohammad (Ilorin; General Hospital); NT Abdulraheem, A Jimoh , A Lawal, OK Fasiku (Ilorin; University of Ilorin Teaching Hospital); B Aminu , S Kache, G Yohanna Abrak (Kaduna; Barau Dikko Teaching Hospital); AA Sheshe, L Anyanwu, AB Muhammad, A Abubakar Abdulkarim , TN Nagwamutse, IU Garzali, S Muhammad, C Nwachukwu, IE Suleiman, M Abdullahi , SA Aji, A Dahiru, LB Abdullahi, SA Yunusa , A Yahaya, M Bello, I Wasiu, U Mohammed Bello, B Yunusa, N Umar (Kano; Aminu Kano Teaching Hospital); RE Enejo, N Nwafulume, A Oke, J Taiwo (Lokoja; Federal Teaching Hospital Lokoja); OH Ekwunife, OA Egwuonwu, OA Okoye, N Nwanne, C Ugwunne, J Ugwu, U Ezidiegwu, CD Nwosu, K Oluchukwu , V Modekwe, C Uche, EA Obiesie, C Osuigwe (Nnewi; Nnamdi Azikiwe University Teaching Hospital); OH Ekwunife, J Aseme, U Edith, J Ezeh, J Nnoli, H Willy-Chidire, D Chimkaomasiri (Onitsha; Holy Rosary Specialist Hospital); A Ojewuyi (Osogbo; UNIOSUN Teaching Hospital); I Ogundele, A Adekoya, L Amosu, A Oyedele, A Ayoade, BA Ayoade, A Asekun , A Ajayi, O Popoola, M Yinusa, AAA Oyelekan, O Oluyemi, C Nwosu, M Okudero, M Agunloye, A Ojo, C Nwokoro, I Babajimi-Joseph , A Williams, S Ogunlade (Sagamu; Olabisi Onabanjo University Teaching Hospital); M Daniyan, TT Sholadoye, M Bashir, N Oyelowo, SE Nwabuoku , A Yakubu, O Ogunsua, M Abubakar , MA Tolani, MS Aliyu (Zaria; Ahmadu Bello University Teaching Hospital).

North Macedonia: T Risteski, V Naunova, L Jovcheski (Skopje; University Clinic for Pediatric Surgery).

Oman: PW Haque, A Albatanony, A Fajardo, M Omer, DP Talreja, AM Elsayed , H Habiba (Ibra; Ibra Hospital); F Ali, S Alsibai, B Dawud, J Albalushi , AM Al Balushi, A Alzadjali, T Al Barhi, A Alkharusi, F Alfarsi, K Al Hinai, H Al Qadhi, R Al Shehhi, H Al Miskry, O Al Hamdani, O Al Alyani, B Rehmani , D Ghosh, M Al-Attraqchi, Z Al Balushi (Muscat; Sultan Qaboos University Hospital); M Al Hinai, S Alwardi, L AlRiyami, A ALJamoudi, M Masaaod (Nizwa; Nizwa Hospital).

Pakistan: A Raza, A Hai, S Ahmed, DS Maqsood, S Chaudhary, M Farooq, M Tayyab, Z Qureshi, M Ali, S Maqbool, U Abdullah, M Aziz, A Irshad, S Said, A Zafar, U Akram, I Sadiq, A Abbas, M Siddique , I Shahbaz (Islamabad; Dr Akbar Niazi Teaching Hospital); SH Waqar, M Raheem, F Akhtar, DM Mehmood, DN Mahmood (Islamabad; The Pakistan Institute of Medical Sciences); M Shahid, A Ali, M Ahmed, M Mansoor Iqbal, Y Lakdawala, S Otho, M Khalid, M Masood, R Kumar, S Jabeen (Karachi; PAF Faisal Hospital); S Altaf (Karachi; Patel Hospital); M Abdullah, G Shamsi, I Ahmed , N Lodhi, G Awais, L Rai (Karachi; The Indus Hospital); S Khattak, A Janjua, A Liaquat, M Saleem (Lahore; Bahria International Hospital, Bahria Orchard); MN Rafique , MM Bin Khalid, H Ahmad, MS Khalid, MH Sadiq, A Hashmi , HH Shahid, MA Sadiq, MH Chishti, M Usama, M Kashif, AM Choudhary , H Basharat, K Khalid, MA Haider, MA Bashir, H Sabir, MF Tarar, M Usama (Lahore; Services Hospital Lahore); MB Mirza, WU Rehman, W Tahir, R Khalid, CE Azmat (Lahore; The Children’s Hospital & The Institute of Child Health Lahore); H Qayum, H Irfan Khan, A Mustafa (Peshawar; Mercy Teaching Hospital); HW Bhatti, MR Farooqui, F Rauf, NA Malik (Rawalpindi; Benazir Bhutto Hospital); M Usman Malik, S Hayat, D Riazhussain (Sargodha; District Headquarter & Teaching Hospital - Sargodha).

Palestine: J Najajra, N Al-Hroub, M Abu Daoud, R Jubran, M Srour, M Taamreh, A Alsalahat, H Masalma (Bethlehem, West Bank; Beit Jala Governmental Hospital (Al Hussein)); A Alwali, A Alwali, L Mohammed, M Al zebda, S Mahdi, A Shaheen, G Alrayyes, L Tafesh, T M. Abubasheer, M Abu Jayyab, H Jaber, M Abuwarda, B M. J. Alhaj, T Aldirawi, F Mahmoud, M Ali, A Albhaisi, M Obaid, WJN Almadhoun, A Alroobi (Gaza; Al-Shifa Hospital); M Abo Abdo, A Abuthaher, A AbuNemer, M Abu Al Amrain, I Nasser, A Awad, A AlAgha, R Madi, D AbuNemer (Gaza; Nasser Hospital); N Kullab, H Elhallaq, A Abu Tair (Gaza; Palestine Red Crescent Society - Al-amal Hospital); H Ayesh (Hebron, West Bank; Al-Ahli Hospital); H Abu-Arish, A Zamareh, M Ahmoud, M H. Oweidat, I AlJada, M Anati, S Halabi, W Alhroub, A Abuhammad, S M. Udwan, H Yaghmour (Hebron, West Bank; Governmental Hebron Hospital-Alia); D Houmran , NA Awwad, M Abed, I HajMohammed, A Hewari, A Alqerem, E Zidan, H Abbadi, S Abed (Jenin, West Bank; The Martyr Dr. Khalil Sulaiman Hospital (Jenin Governmental Hospital)); M Shakhshir, M HajHamad , M Saifi, S Abuzahra, A Khouli, Z Shabello, Z Khraim , S Ismail, MF Dwikat (Nablus, West Bank; Rafidia Hospital); R Bassam, A Sabbah, A Gharib, R Alzughayyar , R Issa, A Abuhantash, O Matar, Y A. Omar, O Khalil, A Awwad (Tulkarm, West Bank; Martyr Thabet Thabet Govermental Hospital).

Paraguay: A Rodriguez Gonzalez, ED Sosa Ferreira, R Ferreira Acosta , MN Martínez Bareiro, JE Giubi Bobeda, A Franco (Asuncion; Hospital de Clínicas, II Cátedra de Clínica Quirúrgica, Universidad Nacional de Asunción).

Peru: A Walfor (Huánuco; Hospital Regional Hermilio Valdizán Medrano); L Poggi, L Poggi, L Fuentes Rivera Lau, MA Moreno Gonzales, F Camacho, O Ibarra, G Arredondo (Lima; British American Hospital); K Nieto Yrigoin, D Chavez Fernandez, DC Juan Carlos (Lima; Clinica Internacional); G Mendiola, A Salazar, R Casma Bustamante (Lima; Hospital Santa Rosa de Lima); G Borda-Luque, Y Carpio Colmenares, MR Li Valencia, F Palomino Escalante, K Quispe de la Roca (Lima; SANNA - Clínica El Golf); MM Caramantin Obando (Paita; Hospital I Miguel Cruzado Vera EsSalud); R Polo, V Serna-Alarcon (Piura; Jose Cayetano Heredia III Regional Hospital).

Poland: R Mazurkiewicz, M Kołomańska, PJ Milewski (Kielce; Wojewódzki Szpital Zespolony w Kielcach); M Kisielewski, T Stefura, K Richter, W Wysocki, N Kłos , W Jabłoński, I Alsoubie, B Żaczek, T Wojewoda, J Bolanowski (Krakow; 5th Military Clinical Hospital); Z Orzeszko, M Wikar, R Solecki, B Markowska, M Szura, T Gach (Krakow; Brothers Hospitallers Hospital); M Matyja, B Habrat, W Serednicki (Krakow; Jagiellonian University Medical College); Z Lorenc, M Święch, M Mietła, W Krawczyk, M Nycz (Sosnowiec; Wojewódzki Szpiital Specjalistyczny nr 5 im. Św Barbary); K Urbańska, K Komorowska, P Kowalewski, M Walędziak (Warsaw; Military Institute Of Medicine); JK Zajac, M Zawadzki (Wroclaw; Regional Specialist Hospital in Wroclaw); M Kusiński, M Pryt, F Brzeszczyński , H Dąbrowski, M Redynk (Łódź; Copernicus Memorial Hospital).

Portugal: J Figueiredo, B Cismasiu, R Souto, S Henriques, AL Preto Barreira, J Vaz, JM Carlos, M Trindade, L Moreira, M Palas, J Simoes (Almada; Hospital Garcia de Orta); F Ramalho de Almeida, M Vasconcelos, A Neves, J Ribeiro, F Afonso , A Pita, R Miranda Pera, M Bernardo, C Rio Ferreira, T Branco, J Fontaínhas, S Pimentel Morais, B Pinto (Amadora; Hospital Prof. Doutor Fernando Fonseca, E.P.E.); S Patrocínio, L Moniz, C Rolo Santos, P Bernardo, F Nazareth (Barreiro; Centro Hospitalar Barreiro Montijo, EPE); C Silva, L Heeren, AR Mateus Loureiro, B Tinoco, A Abreu (Caldas da Rainha; Hospital das Caldas da Rainha - Centro Hospitalar do Oeste, E.P.E); DM Gonçalves Múrias Gomes, C Figueiredo, C Aguero, M Reia, M Guerrero, MA Fernandez Romero, J Dominguez (Elvas; Hospital Santa Luzia Elvas); I Colaço, M Nunes Luís, S Andrade, S Oliveira, D Pais (Figueira da Foz; Hospital Distrital da Figueira da Foz); DG Alves, F Castro, R Ribeiro, I Mogárrio, MDC Gama Caldeira (Funchal; Hospital Dr. Nélio Mendonça); B Gama, CS Rodrigues, A Cabral, A Silva (Horta; Hospital da Horta, E.P.E.); E Borges, J Cassiano Neves, R Bernardino, P David Santos, J Secchi (Lisbon; Centro Hospitalar Lisboa Norte); M Nunes, D Tavares, M Cruz , C Quintela, C Cardoso, IM Lourenço (Matosinhos; Unidade Local de Saude de Matosinhos - Hospital Pedro Hispano); D Vaz Acosta, P Rego Ponte, R Santos Pereira (Ponta Delgada; Hospital do Divino Espírito Santo); H Capote, MB Mourato, T Mogne, N Andrade, G Fialho, F Valente Costa Pinto, B Cordeiro, M Brito, G Santos, D Rosado, C Costa, N Pratas (Portalegre; Hospital Doutor José Maria Grande); J Dias-Ferreira, AL Carreira-Marques, R Ribeiro Dias, B Carvalho, M Gomes, C Soares-Aquino, F Gomes, S Barbosa Castelo Branco, C Coutinho, JP Vieira de Sousa, D Atouguia (Porto; Centro Hospitalar e Universitário de São João); L Cidade Costa (Porto; Hospital da Prelada); D Silva, P Correia, C Henriques, AM Pinheiro Pereira, J Marques Antunes (Santa Maria da Feira; Centro Hospitalar Entre o Douro e Vouga); H Devesa, R Barradas, S Fortuna Martins, N Marcos, A Jarimba, B Louro, L Rodrigues Madeira, AR Lourenço (Santarem; Hospital de Santarem); A Ferreira, A Abreu da Silva, D Stoian , M Ferreira (Santiago do Cacém; Hospital do Litoral Alentejano); R Branquinho, JC Domingues, MI Seixo, R Lalanda, C Bôto (Tomar; Centro Hospitalar Médio Tejo); J Fernandes, P Laranjo , M Reis, I Borges da Costa, C Assis, B Lopes Patrício, NM Freitas Oliveira (Vila Franca de Xira; Hospital Vila Franca de Xira); M Carvalho, J Mendes, C Macedo Cardoso de Oliveira, B Freire, R Pinheiro Duque (Vila Nova de Famalicao; Centro Hospitalar do Medio Ave); B Vieira, U Fernandes, A Dupont, J Ribeiro, R Vaz Pereira (Vila Real; Centro Hospitalar de Trás-os-Montes e Alto Douro, E.P.E.).

Qatar: G Sarp, E Soyer Güldoğan (Doha; Turkish Hospital).

Romania: É Gáspár (Brasov; Regina Maria); A Chitul, C Bezede, E Ciofic, D Cristian (Bucharest; Coltea Clinical Hospital); EA Toma, IM Matache, O Enciu, B Bogdan-Gabriel (Bucharest; Elias Emergency Hospital); I Negoi, C Ciubotaru, I Tanase, C Dina, VM Negoita, A Perja (Bucharest; Emergency Clinical Hospital Bucharest); R Drasovean, A Trif, D Misca, C Hossu, I Imihteev (Cluj-Napoca; Medicover Hospital Cluj).

Russian Federation: V Kakotkin, M Agapov, V Budyakova, S Dos Santos Rocha Ferreira, R Senin (Kaliningrad; Immanuel Kant Baltic Federal University, Regional Clinical Hospital); A Bedzhanyan, A Sumbaev, K Petrenko, E Bedzhanyan, E Tyurina, R Azimov, P Glushkov, K Shemyatovsky , S Husanov, A Sidorova (Moscow; Petrovsky National Research Centre of Surgery); G Yarovenko, E Shestakov, O Lisin, A Arustamyan, S Katorkin (Samara; Hospital Surgery Clinic of Samara State Medical University); J Sidorovskaia, K Cholah, I Cholah, D Kurochka (Simferopol; Municipal Emegency Hospital No.6); V Ten, Y Kudryavcev (Yuzhno-Sakhalinsk; Private healthcare institution ‘RZD-Medicine’).

Rwanda: JP Rugambwa, CN Nelly Rosine, N Jeannette, A Dusabimana (Huye, Gisagara; Butare university teaching hospital (CHUB)); C Seneza , C Uwakunda, L Mukamazera, G Ntwari, I Didier (Kigali; Kibagabaga Hospital); A Costas-Chavarri, M Eugene, C Nyampinga, R Munyaneza, D Muyenzi (Kigali; Rwanda Military Hospital).

Saudi Arabia: N Alzerwi, M Rayzah, A Almutairi, A Alsultan, B Ali (Al-Majmaah; King Khalid General Hospital); A Shabkah, O Ibrahim, H Said, A Alhebshi, A Mohsen, K Anaam, F Alnazawi, F Haddad, A Basalim, B Albaihani, S Al Athath, A Jowharji, A Aljahdali (Jeddah; International Medical Center); N Trabulsi, M Alharthi, A Farsi, M Ghunaim, A Nawawi, A Maghrabi (Jeddah; King Abdulaziz University Hospital); N Alzerwi, Y Aldebasi , F Al Abbood, A Elkhalifa, M Alshanwani , S Alshagrawi (Riyadh; King Salman Hospital in Riyadh); F Ahmad (Riyadh; King Saud Medical City); A Alayed, R AlQahtani, R Sugair, O Alruwaili, B Alsharari, H Albalawi, H Al SOHABI, A Alshahrani , S Asiri, M Alshehri, A Albalawi , R Alatawi , T Khewater, H Adi, J Akiely, N Musawa, F Alahmad, B Alqahtani, M Sersarah, Z Farraj, DY Alalawi (Tabuk; King Salman Armed Forces Hospital); A Alzahrani , N Al Amri, M AlThomali, D Elkafrawy (Taif City; King Faisal Medical Complex).

Serbia: J Juloski, V Cuk, V Cijan, L Milic (Belgrade; Zvezdara University Medical Center).

Slovenia: JA Košir, J Grosek, A Tomazic, T Košir Božič (Ljubljana; University Medical Centre).

South Africa: S Gumede, C Kloppers, K Booyse, S Dos Santos, M Flint (Cape Town; Groote Schuur Hospital); Z Johnson, JJ Jordaan, G Steenkamp (Cape Town; Karl Bremer Hospital); K Nieuwenhuys, J Uys, SS Verhage, A Goliath, S Gilbert (Cape Town; Khayelitsha District Hospital); M Kariem, N Karimbocus (Cape Town; Mitchell’s Plain District Hospital); C Lategan, T Mabogoane (Cape Town; Victoria Hospital Wynberg); S Mewa Kinoo, R Naidoo, N Ntanzi, S Sibiya, S Ebrahim (Durban; King Edward VIII Hospital); S Govender, E Naidoo, P Moodley, K Maharaj (Durban; Stanger Hospital); H Le Roux, J Van Niekerk (East London; Cecilia Makiwane Hospital); A Sparke, P Omwansa (East London; Frere Hospital); CA Baars, S Marawu, K Sevnaran, A Szpytko (Empangeni; Ngwelezana Hospital); G Charalambous, B Van Zyl, O Pheiffer, F Roodt (George; George Hospital); D Rattray, N Rasool, M Nkogatse , R Mackay, G Urdang (Johannesburg; Edenvale); V Manchev, D Clarke, S Naidu, V Govindasamy (Pietermaritzburg; Edendale Hospital); D Montwedi (Pretoria; Kalafong Academic Hospital); LC Kolongi , C Elliot-Wilson, S Kalenga , I Serfontein, M Goga (Upington; Dr Harry Surtie Hospital); S Burger, R Duvenage (Worcester; Worcester Provincial Hospital).

Spain: H Aguado López, F Ruescas, A García Marín, M Scortechini, M Jurado Román , A Sanchez Gallego (Albacete; Hellín Hospital); E González Marín, S De la Cruz Ahufinger, M Mateu, MJ Medina, MDM Martí-Ejarque, R Soliva Domínguez, L Ruiz-Villa, E Montalbán Martínez (BARCELONA; Hospital Universitari Sagrat Cor); A Torroella, C Ginesta, G Cárdenas Rivera, VE Gonzabay, JD Acevedo Parrales (Barcelona; HM Nou Delfos); M Canals Sin, A Lombardero, B Capdevila Vilaró, M Carbonell Pradas, ME Muñoz Fernández , RA Hernandez Rodriguez, I De Haro Jorge, M Riba Martínez, L Tapia Moral, M Coronas Soucheiron, P Palazon Bellver, L Ortega Lechuga, X Tarrado, A Domenech Plana, J Prat-Ortells, M Bejarano Serrano, M Cuesta Argos, MP Martin Gimenez, SG Laura, R Ripoll i Palmés (Barcelona; Hospital Sant Joan de Deu); A Sainz Lete, JC Zevallos-Quiroz, D Gómez, B Estraviz, JM De Francisco Rios, J Barrutia Leonardo, M González de Miguel (Bizkaia; Hospital Urduliz); RL Ferlini, M Ortega Escudero, Y Galvañ Félix, C Hernandez Diaz, J Montero García (Burgos; Hospital Universitario de Burgos); Á Fernández Camuñas, EP Garcia Santos, FJ Redondo Calvo (Ciudad Real; Hospital General Universitario de Ciudad Real); M Estaire Gómez, RJ Castro Lara, A Ramos Bonilla, L Rodríguez Gómez, M Marqueta De Salas, A Alvarez Cuiñas, FM Bujalance Cabrera, MD Cancelas, A García Domínguez, G Chamoso Mialdea, EP Cagigal Ortega, D Enjuto, I Cervera (Leganés; Severo Ochoa University Hospital); R Villalobos Mori, Y Maestre González, C Gas, L Codina Corrons, C Semeraro (Lleida; Hospital Universitari Arnau de Vilanova); J García-Quijada, TW Jorgensen, L Marquez (Madrid; Hospital Central de la Cruz Roja San Jose y Santa Adela); MJ Peña Soria, N Tabatabaian , JL Garcia galocha, D Fra Corral, L Sante Serna (Madrid; Hospital Clinico San Carlos); M Diez Alonso, C Vera Mansilla, L Casalduero, S Soto Schütte, Y Allaoua (Madrid; Hospital Universitario Principe de Asturias); E Gutierrez, C Zapata Syro, F Prieto La Noire, MDM Olmedo Reinoso, S Salido, N Chavarrias, M Vicario Bravo, L Asensio Gomez, R Abad, A Gegúndez Simón, PC Arteaga Asensio (Madrid; Hospital Universitario la Paz); AM Minaya Bravo, E González , A Galvan, C Guijarro Moreno, G De la Peña González, A Sánchez Gollarte, A Robin Valle de Lersundi, MÁ García Ureña (Madrid; Hospital del Henares); JL Rodicio Miravalles, AA Suárez Álvarez, DW Silva-Cano, G Martínez Izquierdo, P Del Val Ruiz, M Moreno Gijon, S Amoza Pais, GP Ibero Casadiego , E López-Negrete Cueto, J Carrizo, S Sanz, R Rodríguez-Uria, A Cembellin, G García-Santos, A Fraile (Oviedo; Hospital Universitario Central de Asturias (HUCA)); D Córdova García, L Jiménez, J Martin Fernandez, R Alvarado Hurtado, AM Minaya Bravo, R Díaz Pedrero, N Cobeño Tamayo, V Ongil Rodríguez (Rivas-Vaciamadrid, Madrid; Hospital Universitario HM Rivas); F Aguilar del Castillo, Á De Jesús Gil, S Borrego Canovaca (Sevilla; Hospital Universitario Virgen del Rocio); JR Naranjo Fernández, Z Valera Sanchez, R Perez, M Infantes Ormad, M Sánchez Ramirez (Seville; Hospital Universitario Virgen Macarena); C Leal Ferrandis, C Esteo Verdu, S García López, J Febré, B Cuneo (Valencia; Hospital Arnau de Vilanova); C León-Espinoza, E Martí Cuñat, G Pou (Valencia; Hospital Clínico Universitario de Valencia); C Jezieniecki, S Alonso Marcos, A Vazquez Fernandez, J Beltrán de Heredia, B De Andrés-Asenjo, D Baños Méndez, JC Garcia Vera, M Ruiz Soriano, E Redondo, R Martínez Díaz , T Gómez Sanz, P Artigot, C Infante, C Ferreras García, LR Cabezudo, G Cabezudo, M Lainez Escribano, M Rodriguez-Lopez, H Nuñez Del Barrio, A Romero de Diego (Valladolid; Hospital Clínico Universitario de Valladolid); A Vazquez Melero, M Camuera, I Herrero, D Garcia López de Goicoechea, M Sánchez-Rubio (Vitoria-Gasteiz; Hospital Universitario Araba); MDP Cebollero, JL Blas Laina (Zaragoza; Hospital Royo Villanova); V Duque Mallén, N Sánchez Fuentes, P Sancho Pardo , I Gascon Ferrer, MÁ Dobón Rascón, T Gimenez Maurel, J Chóliz, S Saudí-Moro, S Paterna -Lopez, A Martinez German, D Aparicio-López, MÁ Gascón Domínguez, P Royo Dachary (Zaragoza; Hospital Universitario Miguel Servet).

Sri Lanka: S Srishankar, SPB Thalgaspitiya, KJ Senanayake, D Wickramarathna (Anuradhapura; Teaching Hospital Anuradhapura); D Subasinghe, D Wickramasinghe (Colombo; National Hospital of Sri Lanka); M Nandasena, K Wijesinghe, H Miyasika, J Senavirathna, Y Chamara (Dehiwala; Colombo South Teaching Hospital); S Rajendra, SI Thuraisamy Sarma, B Balagobi, V Sutharshan , S Giridaran (Jaffna; Teaching Hospital, Jaffna); W Wijenayake, MT Ekanayake, S Jayatilleke, S Jayasekara, M Perera, R Perera, R Ellawala, WDD De Silva (Werahera; University Hospital, Kotelawala Defence University).

Sudan: S Alqurashi , N Rajab (Ed Dueim; Ed Dueim Teaching Hospital); TA Albushary , FA Mohammed Daoud , A Younis, MA Suliman, F Tahir Lwdie, S Ibrahim Tour Harakan, M Abdelhadi Suliman Adam , E Hegab, A Abdalla, T Almahdi , E Alabed (El Geneina; El Geneina teaching Hospital); M Ahmed, S Eldirdiri, M Salah, OA Eljizoly , A Mohammed, A Mohamed Ibrahim Mohamed (Gadarif city; Gadarif teaching hospital); A Albager, MA Ismael Alamin, M Alsalawi, MA Elgak (Kassala; Police hospital); EG Nubi Mohamed, A Eltahir, E Adel Hamdoun Aziz, I Adel, O Emadeldeen, OG Nubi, E Mohamed (Khartoum; Bashair Teaching Hospital); M Hamed, M Tageldin, E Elsheikh, U Omara, E ADAm, IMG Ahmed, GMG Ahmed, S Imam (Khartoum; Ibrahim Malik Teaching Hospital); AA Adam, S Amin Omar Alsiddig, A Ahmed, S Abdelrasoul Elnour Ismail, M Mohamedshafee, Y Mohamed (Khartoum; Omdurman Teaching Hospital); EE Abuobaida Banaga Hag El Tayeb, H Abuobaida , AS Ahmed (Khartoum; Ribat university hospital); A Elbalal, IMG Ahmed, GN El Hunjul, GMG Ahmed, A Mustafa, HA Fadlalmola (Khartoum; Soba University Hospital); A Mohammed, E Yousuf, E Hamed, S Ibrahim, O Morgan, N Omer (Wad Madani; University of Gezira Hospital).

Sweden: M Zaigham, A Al Mukhtar (Malmö; Skåne University Hospital); M Nikberg (Vasteras; Västmanlands Hospital Västerås).

Switzerland: D Fenner, D Salinovic (Frauenfeld; Spital Thurgau AG); X Papazarkadas, TV Pham, C Brasset, A Litchinko, F Ris, C Golliez, M Chevallay (Geneva; Geneva University Hospitals); J Gass, J Mühlhäusser, J Metzger, A Scheiwiller (Luzern; Luzerner Kantonsspital); A Tampakis, C Riboni, U Dietz, E Brolese, C Seiler, M Kalisvaart, JN Marx, L Eisner (Olten; Kantonsspital Olten); G Peros, F Solimene, M Gramellini, A Lareida, M Adamina, E Betz, L Dubs, K Geiger-Timm, L Gantner, N Seeger, K Richetti, K Hofmann (Winterthur; Kantonsspital Winterthur); MA Schneider, D Gero, K Lehmann, P Limani, S Hügli, S Gerdes, F Mazzola, A Hiller (Zurich; University Hospital of Zurich).

Syrian Arab Republic: MA Farho, M Mohammad, AY Arnaout, Y Nerabani, Y Maktabi, W Alsado, A Anadani (Aleppo; Abd Al Wahab Agha Hospital); M Morjan, M Nasani, W Mayo, S Kreid, M Arnaout, MN Sawas (Aleppo; Al-Shahbaa Private Hospital); M Aloulou, MK Marawy, A Kezze, I Arnaout, A Kelzia, A Ghazal (Aleppo; Aleppo Private Hospital); A Ghazal, E Dabbagh, R Masri, MH Nabhan, A Alniemi, A Alhaj, S Ward, Y Haido, N Dadoush, MK Abu albahrain, A Niazi, W Abbas, A Hasan, S Alshab, S Kamari, R Kalouk, Z Toutounji (Aleppo; Aleppo University Hospital); D Sharl Ajami, B Alsaid, A Alusef, K Abo zaal (Damascus; Al assad university hospital); AR Hammadieh, Z Klib, MR Mslmani, A Alhaj zain, M Klib, L Shammas, AJ Chekfa, Z Odeh, R Joumaa, H Al-zoubi (Damascus; Al-Mouwasat University Hospital); Q Mashlah, HO Odah Bashi̇, H Zwaraa (Damascus; Children’s University Hospital); I Adham, L Hasan, A Khatib, S Jomaa, A Alfarwan, A Torbey, A Rashid, A Hawarah, A Ali, M Alhimyar, L Al-Boukhari, MA Al-yusuf, AN Aldirani (Damascus; Damascus Hospital); Y Alhammoud , Y Al-Junaidi (Homs; Al-Basel Specialized Hospital in Karm El-Louz); M Daher, Z Asaad, A Abbas, K Khalil (Homs; The Military Hospital); J Khoury, L Hasan, J Jahjah, J Alaji, S Turkmani, S Mahfoud, A Ahmad, M Derattani, G Massarra , J Skaff, ZA Hannouneh, H Amoudi, ZA Zaher, G Zaza (Latakia; Al Saydeh Surgical Hospital); A Wassouf, A Alahmad Alismael , S Nofal, A Mansour, M Mansour, Z Alkhaier, J Suliman, M Sabboh, M Haj Hussein, M Ibrahim, ZA Abo alaros, G Alhadwah , N Kheyrbek, D Ibrahim, G Hamdan, Y Hasan, A Abo al Shamat, A Roumieh, B Khattab, H Alkhatib (Latakia; National Hospital); S Hassan, S Abdul Rahman, A Abdul Rahman, F Aliskander, J Alkharish, G Kafa, I Suleiman, A Bassma, A Alloush , N Ismaiel, J Deeb, M Alrantisi, E Salloum, A Al-Mouahhed , A Baydoun, H Yunes, S Alkadi, F Ali, D Abdulrahman, I Hussein (Latakia; Othaman Hospital); A Bakri, H Asaad, T Ashkar, H Daaboul, A Marouf, F Chahrour, B Ranjous, B Ibrahim , A Sinjab, M Alneasan, N Ali, A Alloush, J Fahed, R Attaf, S Kanaan (Latakia; Tishreen University Hospital).

Thailand: A Tansawet, W Kasetsermwiriya, I Laopeamthong, P Sukhvibul, T Techapongsatorn, N Techapongsatorn, P Kasetsermwiriya , P Leungon (Bangkok; Vajira hospital).

Togo: PS Tekam Wadje (Lomé; CHU Sylvanus Olympio).

Tunisia: MJ Kacem, R Elaifia, Y Ouadi, S Megdiche, Y Jedidi (Tunis; La Rabta Hospital).

Turkey: AÇ Bozkurt, H Tümer (Adana; Adana Seyhan State Hospital); MA Koç, A Çakmak, AF Kocaay, KY Türker, TB Türkmen (Ankara; Ankara University Medical School); O Yalkın, D Yigit (Bursa; Bursa City Hospital); B Yigit, A Aslan, S Yılmaz (Elazig; Elazig Fethi Sekin City Hospital); AN Sanli, Yİ Tandoğan, A Yildiz, A İsler, A Ozkomec (Gaziantep; Abdulkadir Yuksel State Hospital); ME Seker, E Ay, M Erkaya, YO Koyluoglu, A Develioğlu, GM Kurtoglu, GK Kurtoglu, AF Cetişli, E Tunçcan, A Aghayeva, Z Durna, B Baca (Istanbul; Acibadem Altunizade Hospital); AE Dönmez, B Togay, E Ada, IE Yavuz , IA Bilgin, EC Karabulut, AM Uysal, Y Karataş, B Ağca, MK Aktas, F Demiral, B Duman, K Kabulov, I Hamzaoglu, T Karahasanoğlu, M Tanal, E Tuzuner (Istanbul; Acibadem Maslak Hospital); S Meriç, M Tokocin, H Yigitbas, A Barcin, G Alıcı, E Yavuz, OB Gülcicek, N Bugdayci , K Özdoğan, I Çakır, YE Aktimur, Y Altinel (Istanbul; Bagcilar Research And Training Hospital); ÖP Zanbak Mutlu (Istanbul; Bahçelievler State Hospital); RE Sönmez, M Şermet, MS Ozsoy, H Baysal, F Buyuker (Istanbul; Istanbul Medeniyet University, School of Medicine); M Oncel, S Bektas, AE Askin, M Yashar, A İzgi̇ş (Istanbul; Istanbul Medipol University Hospital); SS Uludağ, MF Ozcelik, S Yumurtacilar (Istanbul; Istanbul universty - Cerrahpaşa Medical faculty); A Özcan, E Somuncu, S Yilmaz, A Sapmaz, H Bolukbasi, C Özkan, E Bozdağ, MC Kizilkaya, H Telci, Y Kara, AZ Kaan, M Acar, EO Yildirim (Istanbul; Kanuni Sultan Suleyman Training and Research Hospital); G Akcakoca, C Hacıalioğlu, Y Tosun, V Çalik, TE Yılmaz, H Alfakeer (Istanbul; Kartal Dr. Lutfi Kirdar Training and Research Hospital); MI Ateş, SN Karahan, M Kalender, AE Narin, D Yi̇ği̇t, O Agcaoglu, S Toprak, B Celik, E Ozoran, DS Uymaz, S Yigman, E Bozkurt, A Rencuzogullari, E Balik, S Sucu (Istanbul; Koç University Medical School); A Akmercan, K Oğur, A Hajali, QK Dolatzay (Istanbul; Marmara University, School of Medicine); E Unal (Istanbul; Sehit Prof.Dr. İlhan Varank Training and Research Hospital); N Kiziltoprak, MS Genç, B Özcan, Z Şenol, OF Ozkan, M Çuhadar, ED Terzi (Istanbul; Sultan 2. Abdulhamid Han Training and Research Hospital, University of Health Sciences); T Gülşen (Istanbul; Sultanbeyli State Hospital); MT Demirpolat (Istanbul; University of Health Science Umraniye Education and Research Hospital); B Citgez, H Ozsahin, C Ersavas (Istanbul; Uskudar University Faculty of Medicine, Memorial Hospital); C Bi̇li̇r, AE Boztaş Demi̇r, AD Hacioglu, G Ozyuksel, H Ulman (Izmir; Bakircay University Cigli Training and Research Hospital); AM Öztürk, B Calik, AC Yaşar (Izmir; University of Health Sciences Izmir Bozyaka Training and Research Hospital); E Colak, MA Avci, E Aybar, M Gün, AB Ciftci, MS Uyanik, ME Kara, C Akgün, AC Sarı, Ö Küpçüoğlu, GO Kucuk, S Polat (Samsun; Samsun University Samsun Training and Research Hospital); N Kavak, MA Kara, G Karadeniz Cakmak, B Kum (Zonguldak; Zonguldak Bulent Ecevit University School of Medicine Research and Training Hospital); S Öztürk, B Eyduran (İzmir; University of Health Sciences Tepecik Training and Research Hospital).

Uganda: B Kigwe, M Arafat (Iganga; Iganga district hospital); M Nnabagulanyi (Kigumba; Kiryandongo Hospital); S Stonelake (Luwero; Kiwoko Hospital).

United Kingdom: ZA Fozo, A Shoker, KR Rahman, MW Saqib, R Faisal, CS Ong, A Pillai, H Unwin, A Huws, M Maybury, H Ejaz, E Daketsey, AK Lala (Bangor, North Wales; Ysbyty Gwynedd); MAK Sarker, B Chkir, MR Peris, S Khan , MA Tahir, N Sharma, R Doherty, H Alhusaini, MF Butt, H Afzal, S Handa, N Maharjan , A Mostafa, G Lee, CK Lim, A Anand, A Krishna, WT Yew, Y Lu, R Hall (Barrow in Furness; Furness General Hospital); E Mohammed (Basildon; Basildon University Hospital); F Georgiades, S Karim, K Rajaratnam (Bedford; Bedford Hospital); J Abbasy, A Bibi, S Karandikar, L Johnstone , N Fazili (Birmingham; Heartlands Hospital); A Singh, A Athanasiou, H Lidbetter, J Siby, M Kaur, A Fatima (Brighton; Royal Sussex County Hospital); SM Reddy, A Campbell, A Cardoso Almeida, K Smith, CJ Bradshaw, K Tambudze, H Delacave, I Norman (Bristol; Bristol Royal Hospital for Children); M San, S Babu, S Midya, H Bradly, S Tontus, H Chauhan, R Jurdon, M Corcos , E Jose (Camberley; Frimley Health NHS FT - Frimley Park); N Eardley, B Davies, M Ransome, S Ahmed (Chester; Countess of Chester Hospital); S Suresh, A Bavaharan, R Batir, R Sato (Colchester; Colchester Hospital University); N Chidumije, M Ahmed, TY Kwan, F Olaniru, I Parwaiz, LP Cheng (Coventry; University Hospitals Coventry and Warwickshire NHS Trust); N Gokhare Viswanath, K Nanayakkara, A Tibude (Derby; Royal Derby Hospital); O Olajumoke, M Shams, D George, A Amin, M Kausar, S Sellahewa (Dudley; Russell’s Hall Hospital); H Kamal, A Kamal, M Kamal (Dundee; Ninewells Hospital); O Pryer, H Sagar, B Lulham-Robinson, S Dawo, A Nada (Durham; University Hospital North Durham); R Bethune, G Chillarge, AM Myintmo, M Horga, L Andreski, E Ruiz-Daum , T Finlay, I Rakshit (Exeter; Royal Devon and Exeter Hospital); J Bryan, D Joshi, R Marlin, M Battili , YL Aung, L Zeng, S Mathew, I Njere, S Gopaul, A Abbas (Great Yarmouth; James Paget Univeristy NHS Foundation Trust Hospital); G Singh, M Asarbakhsh, S Staight (Huddersfield; Huddersfield Royal Infirmary); R Govindaraju, M Quaunine, S Yassin, A Wilkins, J Walshaw, L Chang, A Mahendran, F Hammett, D Fairbrass, UA Kalu, E Dexter, T Nadeem, N Karunaratne, T Lo, M Pellen (Hull; Hull University Teaching Hospitals NHS Trust); SH Sarwary, J Otote, D Anbu, H Islam, N Morricone (Ilford; King George Hospital); K Lee, E Gimson, M Wilson, C Chiam (Larbert; Forth Valley Royal Hospital); M Solkar, M Bautista, NS Blencowe, A Gupta, J Sutcliffe, A Ahmad, A Peckham-Cooper (Leeds; Leeds Teaching Hospitals); E O’Connell, KE Dey, R Lunevicius, MM Barakat, GR Goodwin, B Devkaran, IC Nzenwa, A Pilavas (Liverpool; Aintree University Hospital); K Bananis, A Alamin, S Bennett , MD Barcelona, A Sharp, F Soggiu (London; Ealing Hospital); E Baili, H Ebied, A Botha, M Haghighat Ghahfarokhi, MMT Youssef (London; Guy’s and St Thomas’ Hospitals); K Theodoropoulou, A Quddus, R Hegy, M Mahran, A Ghanbari (London; Homerton University Hospital); P Kapsampelis, T Chouari, J Saunders, C Boven, I Gerogiannis (London; Kingston); N Karthikeyan, C Karagianni , E Spanoudakis (London; Queen Elizabeth Hospital, Woolwich); H Younus, R Ben Hmida, D Eaton (London; The Whittington Hospital); C Seet, R Bradley, R Roberts (London; University Hospital Lewisham); FN Amir, M Durrani, S Khan, A Tahir (Macclesfield; Macclesfield District General Hospital); A Khalil, E O’Neill, S Ingley, V Bill, P Wilson, M Elmousili, E Chin , C Alphonse, P Suresh, L Devi, C Shelton (Manchester; Wythenshawe Hospital); S Bugren, M Abdelreheem, MMR Azzuz, F Gareb (Margate; Queen Elizabeth the Queen Mother Hospital Margate); DJ Dhillon, MF Khan, B Peter, I Fagiri , T Harris , P Thambi (Middlesbrough; James Cook University Hospital); M Tomlinson, C Hidalgo Salinas, A Mwanjoka , M Catterall, B Ali (Morecambe; Royal Lancaster Infirmary); S Tingle, K Waddell, F Peters, T Akharaekpanya, S Robinson (North Shields; Northumbria NHS Hospital Trust); NA Binti Yusri, A Ashiru, SA Chowdhury , J Reilly, S Malek, S Kumaran, A Doghaim, ABA Al-Hajjaj, LR Chieng, ZY Wong, R Olatunji (Nottingham; Queens Medical Centre); J Bundred, B Down, A Ang, KA Shamiyah, G Bond-Smith (Oxford; John Radcliffe Hospital); M Elmesalmi (Portsmouth; Queen Alexandra Hospital); S Monkhouse , O Mohamed, B Robertson-Jones, P Patel, BZ Hao (Redhill; East Surrey Hospital); S Ghattas, N Beharry, A Maraqa, N Uttam, S Rafiq, A Abdelhamid, E Mazumdar, M Dyer, MEEA Abdelsalam, B Johnson, M Abdelkarim, A Murtada, MMS Tora, Z Azhar (Rhyl; Glan Clwyd Hospital); O Whitehurst, K Bhatti, S Silvestre, LN Bin Aizan, A Patel (Salford; Salford Royal Hospital); Z Khan, A Maqsood-Shah, O Webster, H Reilly, S Boyes (Sheffield; Sheffield Teaching Hospital NHS Foundation Trust); S Bandyopadhyay, B McDermott, H Kynaston, B Neall, M West, G Hart, R Titcombe , H Kaur, A Ekerin, A Demetriou, N Harrison, JJQ Chen (Southampton; Southampton General Hospital); M Hammoda, L Robine-Durnell, E Mansour, A Evans, E Baker (Swansea; Morriston Hospital Swansea); I Abdullah, O Osunlusi (Walsall; Walsall Manor Hospital).

United States: S Soelling, R Askari (Boston; Brigham and Women’s Hospital); M Asaad , J Leong, M Perkins, D Ozal, SS Budhwani (Chicago, IL; Mount Sinai Hospital); A Maxwell, A Shah, S Schimpke (Chicago, IL; Rush University Medical Centre); D Moris, C Nicholson Jr., HE Rice (Durham, NC; Duke University Medical Center); D Ridder, M Tsuruta, K Noguchi, D Mikami, R Kitamura, J Ng-Kamstra (Honolulu, Hawaii; The Queen’s Medical Center); RJ Robitsek, K Fretwell, J Chan, R Laskowski (Jamaica; Jamaica Hospital); R Zerna Encalada (Syracuse; SUNY Upstate University Hospital).

Yemen, Rep.: R Ghaleb, N Alnamari, A Al-Bahla, B Al soudi (Hajjah; Kowaydina hospital); B Alshaikh, M Al-Shehari, M Al-Dhaheri, YSSM Ghaleb, MI Issa (Sana’a; Al-Thawra Modern General Hospital).

**Appendix 2: Supplementary tables and materials**

| **Supplementary table title** | **Pages** |
| --- | --- |
| Supplementary table 1: Sample size calculation | 37 |
| Supplementary table 2: Hospitals included characteristics | 38 |
| Supplementary table 3: Detailed surgical technique variation across income groups | 39 |
| Supplementary table 4: Surgical variation according to surgical approach as intention to treat | 40 |
| Supplementary table 5: Pre- and intra-operative characteristics according to mesh use in open surgery. | 41 |
| Supplementary table 5: Postoperative complications according to Clavien-Dindo classification | 42 |
| **Supplementary figure title** |  |
| Supplementary figure 1: Global distribution of included patients. | 43 |

| **Prevalence** | **C-statistic** | **Parameters** | **Minimum sample size** |
| --- | --- | --- | --- |
| 0.70 | 0.7 | 7 | 569 |
|  | 0.8 | 7 | 240 |
|  | 0.9 | 7 | 120 |
| 0.75 | 0.7 | 7 | 636 |
|  | 0.8 | 7 | 266 |
|  | 0.9 | 7 | 133 |
| 0.80 | 0.7 | 7 | 739 |
|  | 0.8 | 7 | 307 |
|  | 0.9 | 7 | 152 |
| 0.85 | 0.7 | 7 | 925 |
|  | 0.8 | 7 | 381 |
|  | 0.9 | 7 | 186 |
| 0.90 | 0.7 | 7 | 1294 |
|  | 0.8 | 7 | 527 |
|  | 0.9 | 7 | 254 |
| 0.95 | 0.7 | 7 | 2466 |
|  | 0.8 | 7 | 979 |
|  | 0.9 | 7 | 456 |

**Supplementary table 1: Sample size calculation**

**Supplementary table 2: Hospitals included characteristics.**

|  | **HIC**  **(n=303)** | **UMIC**  **(n=145)** | **LMIC**  **(n=146)** | **LIC**  **(n=46)** | **Total**  **(n=640)** |
| --- | --- | --- | --- | --- | --- |
| **Type of hospital** | | | | | |
| Primary level | 946 (10.7%) | 112 (4.4%) | 151 (5.7%) | 105 (16.9%) | 1314 (9.0%) |
| Secondary level | 3332 (37.7%) | 455 (18.1%) | 329 (12.5%) | 69 (11.1%) | 4185 (28.6%) |
| Tertiary level | 4567 (51.6%) | 1950 (77.5%) | 2162 (81.8%) | 447 (72.0%) | 9126 (62.4%) |
| (Missing) | 71 | 58 | 0 | 14 | 143 |
| **Hospital funding** | | | | | |
| Public | 7715 (87.2%) | 1941 (77.1%) | 1912 (72.4%) | 584 (94.0%) | 12152 (83.1%) |
| Private | 552 (6.2%) | 462 (18.4%) | 625 (23.7%) | 37 (6.0%) | 1676 (11.5%) |
| Public-Private | 578 (6.5%) | 114 (4.5%) | 105 (4.0%) | 0 (0.0%) | 797 (5.4%) |
| (Missing) | 71 | 58 | 0 | 14 | 143 |
| **Availability of day-case surgical unit** | | | | | |
| Yes | 8247 (93.2%) | 1968 (78.2%) | 1919 (72.6%) | 528 (85.0%) | 12662 (86.6%) |
| No | 598 (6.8%) | 549 (21.8%) | 723 (27.4%) | 93 (15.0%) | 1963 (13.4%) |
| (Missing) | 71 | 58 | 0 | 14 | 143 |

Data are n (%) unless stated otherwise.

HIC: high-income country, UMIC: upper-middle-income country, LMIC: lower-middle-income country, LIC: low-income country.

**Supplementary table 3: Detailed surgical technique variation across income groups**

|  | **HIC**  **(n=8,916)** | **UMIC**  **(n=2,575)** | **LMIC**  **(n=2,642)** | **LIC**  **(n=635)** | **Total**  **(n=14,768)** |
| --- | --- | --- | --- | --- | --- |
| **Type of hernia repair** | | | | | |
| Lichtenstein | 5176 (58.1%) | 1700 (66%) | 1807 (68.4%) | 434 (68.3%) | 9117 (61.7%) |
| Transinguinal pre-peritonal | 71 (0.8%) | 24 (0.9%) | 55 (2.1%) | 6 (0.9%) | 156 (1.1%) |
| Transrectal pre-peritoneal | 17 (0.2%) | 4 (0.2%) | 10 (0.4%) | 0 (0%) | 31 (0.2%) |
| Plug and patch | 885 (9.9%) | 24 (0.9%) | 38 (1.4%) | 13 (2%) | 960 (6.5%) |
| PHS (bilayer) | 34 (0.4%) | 6 (0.2%) | 3 (0.1%) | 0 (0%) | 43 (0.3%) |
| TAPP | 1650 (18.5%) | 507 (19.7%) | 180 (6.8%) | 2 (0.3%) | 2339 (15.8%) |
| TEP | 848 (9.5%) | 180 (7%) | 126 (4.8%) | 1 (0.2%) | 1155 (7.8%) |
| Other mesh-based | 161 (1.8%) | 20 (0.8%) | 10 (0.4%) | 1 (0.2%) | 192 (1.3%) |
| Primary herniotomy | 20 (0.2%) | 78 (3%) | 32 (1.2%) | 34 (5.4%) | 164 (1.1%) |
| Bassini | 16 (0.2%) | 22 (0.9%) | 153 (5.8%) | 85 (13.4%) | 276 (1.9%) |
| Shouldice | 18 (0.2%) | 5 (0.2%) | 12 (0.5%) | 19 (3%) | 54 (0.4%) |
| Desarda | 15 (0.2%) | 0 (0%) | 32 (1.2%) | 15 (2.4%) | 62 (0.4%) |
| Other non-mesh based | 5 (0.1%) | 6 (0.2%) | 183 (6.9%) | 25 (3.9%) | 219 (1.5%) |
| (Missing) | 0 | 0 | 2 | 0 | 2 |

Data are n (%) unless stated otherwise.

HIC: high-income country, UMIC: upper-middle-income country, LMIC: lower-middle-income country, LIC: low-income country. TAPP: Transabdominal preperitoneal repair; TEP: Totally extra-peritoneal repair.

**Supplementary table 4: Surgical variation according to surgical approach as intention to treat**

|  | **Open**  **(n=11,107)** | **Laparoendoscopic**  **(n=3,508)** | **Robotic**  **(n=153)** | **Total**  **(n=14,768)** |
| --- | --- | --- | --- | --- |
| **Primary operator** | | | | |
| Senior Surgeon | 7270 (65.5%) | 3074 (87.6%) | 143 (93.5%) | 10487 (71.0%) |
| Trainee surgeon | 3744 (33.7%) | 432 (12.3%) | 10 (6.5%) | 4186 (28.3%) |
| Non-surgeon | 93 (0.8%) | 2 (0.1%) | 0 (0.0%) | 95 (0.6%) |
| (Missing) | 0 | 0 | 0 | 0 |
| **Previous experience (nr of repaired hernias)** | | | | |
| 0-50 | 2400 (21.6%) | 337 (9.6%) | 15 (9.8%) | 2752 (18.7%) |
| 51-200 | 3164 (28.5%) | 970 (27.7%) | 33 (21.6%) | 4167 (28.2%) |
| >200 | 5531 (49.9%) | 2197 (62.7%) | 105 (68.6%) | 7833 (53.1%) |
| (Missing) | 12 | 4 | 0 | 16 |
| **Mesh use** | | | | |
| Yes | 10347 (93.2%) | 3495 (99.6%) | 153 (100.0%) | 13995 (94.8%) |
| No | 760 (6.8%) | 13 (0.4%) | 0 (0.0%) | 773 (5.2%) |
| (Missing) | 0 | 0 | 0 | 0 |
| **Type of mesh used*** | | | | |
| Permanent synthetic | 9397 (90.8%) | 3078 (88.1%) | 145 (94.8%) | 12620 (90.2%) |
| Absorbable synthetic | 708 (6.8%) | 281 (8.0%) | 6 (3.9%) | 995 (7.1%) |
| Biological | 12 (0.1%) | 6 (0.2%) | 0 (0.0%) | 18 (0.1%) |
| Composite | 227 (2.2%) | 129 (3.7%) | 2 (1.3%) | 358 (2.6%) |
| (Missing) | 3 | 0 | 0 | 3 |
| **Suture used to fix the mesh*** | | | | |
| Absorbable | 2674 (25.9%) | 372 (10.6%) | 30 (19.6%) | 3076 (22.0%) |
| Non-absorbable | 6835 (66.1%) | 459 (13.1%) | 49 (32.0%) | 7343 (52.5%) |
| Glue | 203 (2.0%) | 418 (12.0%) | 29 (19.0%) | 650 (4.6%) |
| Tackers | 85 (0.8%) | 1270 (36.3%) | 8 (5.2%) | 1363 (9.7%) |
| Not fixed | 547 (5.3%) | 975 (27.9%) | 37 (24.2%) | 1559 (11.1%) |
| (Missing) | 3 | 1 | 0 | 4 |

Data are n (%) unless stated otherwise. *Evaluated only in patients undergoing inguinal hernia repair with mesh (n=13,995).

**Supplementary table 5: Pre- and intra-operative characteristics according to mesh use in open surgery.**

Data are n (%) unless stated otherwise. ASA: American Society of Anaesthesiologists Physical Status Classification System grade.

|  | **No mesh**  **(n=760)** | **Mesh**  **(n=10,347)** | **Total**  **(n=11,107)** |
| --- | --- | --- | --- |
| **Age, years** | | | |
| Median | 49.0 | 61.0 | 61.0 |
| IQR | 32.0 to 63.0 | 49.0 to 72.0 | 48.0 to 71.0 |
| **Sex** | | | |
| Female | 70 (9.2%) | 840 (8.1%) | 910 (8.2%) |
| Male | 690 (90.8%) | 9507 (91.9%) | 10197 (91.8%) |
| (Missing) | 0 | 0 | 0 |
| **ASA grade** | | | |
| ASA I-II | 716 (94.2%) | 8720 (84.3%) | 9436 (85.0%) |
| ASA III-V | 36 (4.7%) | 1575 (15.2%) | 1611 (14.5%) |
| Not recorded | 8 (1.1%) | 52 (0.5%) | 60 (0.5%) |
| (Missing) | 0 | 0 | 0 |
| **Co-morbidities** | | | |
| None | 673 (88.6%) | 7681 (74.3%) | 8354 (75.3%) |
| One | 73 (9.6%) | 2002 (19.4%) | 2075 (18.7%) |
| Two | 10 (1.3%) | 518 (5.0%) | 528 (4.8%) |
| Three or more | 4 (0.5%) | 135 (1.3%) | 139 (1.3%) |
| (Missing) | 0 | 10 | 10 |
| **Symptoms** | | | |
| Asymptomatic | 116 (15.3%) | 1422 (13.7%) | 1538 (13.8%) |
| Symptomatic | 644 (84.7%) | 8922 (86.2%) | 9566 (86.1%) |
| (Missing) | 0 | 3 | 0 |
| **Hernia size** | | | |
| Limited to inguinal region | 494 (65.0%) | 7973 (77.1%) | 8467 (76.2%) |
| Limited to scrotum | 251 (33.0%) | 2249 (21.7%) | 2500 (22.5%) |
| Extend to mid-thigh or beyond | 15 (2.0%) | 125 (1.2%) | 140 (1.3%) |
| (Missing) | 0 | 0 | 0 |
| **Hernia site** | | | |
| Bilateral | 43 (5.7%) | 909 (8.8%) | 952 (8.6%) |
| Unilateral | 717 (94.3%) | 9438 (91.2%) | 10155 (91.4%) |
| (Missing) | 0 | 0 | 0 |
| **Hernia defect size** | | | |
| < 1.5cm | 81 (10.7%) | 1994 (19.3%) | 2075 (18.7%) |
| 1.5cm to 3cm | 336 (44.2%) | 4093 (39.6%) | 4429 (39.9%) |
| >3cm | 280 (36.8%) | 2886 (27.9%) | 3166 (28.5%) |
| Not known | 63 (8.3%) | 1374 (13.3%) | 1437 (12.9%) |
| (Missing) | 0 | 0 | 0 |
| **Contamination** | | | |
| Clean | 734 (96.6%) | 10088 (97.5%) | 10822 (97.4%) |
| Clean-contaminated | 24 (3.2%) | 252 (2.4%) | 276 (2.5%) |
| Contaminated | 1 (0.1%) | 6 (0.1%) | 7 (0.1%) |
| Dirty | 1 (0.1%) | 1 (0.0%) | 2 (0.0%) |
| (Missing) | 0 | 0 | 0 |

**Supplementary table 6: Postoperative complications according to Clavien-Dindo classification**

|  | **No complications** | **Clavien Dindo I-II** | **Clavien-Dindo III-V** | **All complications** |
| --- | --- | --- | --- | --- |
| **Surgical approach*** | | | | |
| Open surgery | 9573 (86.3%) | 1429 (12.9%) | 89 (0.8%) | 1518 (13.7%) |
| Minimally invasive surgery | 3242 (88.7%) | 379 (10.4%) | 36 (1.0%) | 415 (11.3%) |
| (Missing) | 0 | 0 | 0 | 0 |
| **Use of mesh** | | | | |
| Yes | 12148 (86.9%) | 1714 (12.3%) | 114 (0.8%) | 1828 (13.1%) |
| No | 667 (86.4%) | 94 (12.2%) | 11 (1.4%) | 105 (13.6%) |
| (Missing) | 0 | 0 | 0 | 0 |

**Supplementary figure 1: Global distribution of included patients.**


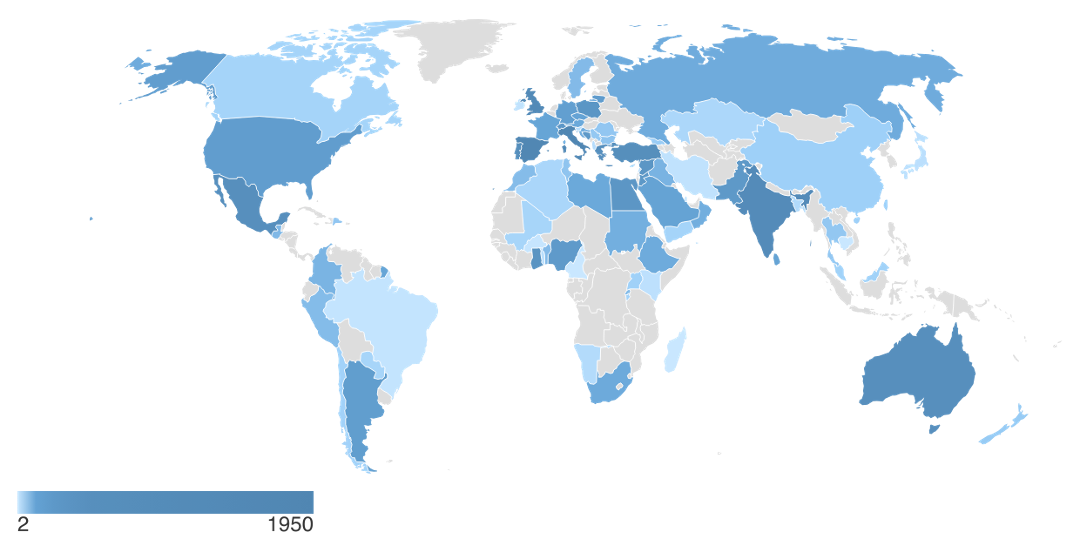

Supplement: znae164_Supplementary_Data [file znae164_supplementary_data.docx]
